# Thermo-responsive emission induced by different delocalized excited-states in isomorphous Pd(ii) and Pt(ii) one-dimensional chains†

**DOI:** 10.1039/d4sc04497e

**Published:** 2024-08-13

**Authors:** Tomoya Saito, Masaki Yoshida, Kaito Segawa, Daisuke Saito, Junichi Takayama, Satoshi Hiura, Akihiro Murayama, Nishshanka M. Lakshan, W. M. C. Sameera, Atsushi Kobayashi, Masako Kato

**Affiliations:** a Department of Chemistry, Faculty of Science, Hokkaido University North-10 West-8, Kita-ku Sapporo Hokkaido 060-0810 Japan; b Department of Applied Chemistry for Environment, School of Biological and Environmental Sciences, Kwansei Gakuin University 1 Gakuen-Uegahara Sanda Hyogo 669-1330 Japan masaki.yoshida@kwansei.ac.jp katom@kwansei.ac.jp; c Faculty of Information Science and Technology, Hokkaido University North-14 West-9, Kita-ku Sapporo Hokkaido 060-0814 Japan; d Department of Chemistry, University of Colombo Kumaratunga Munidasa Mawatha Colombo 00700 Sri Lanka; e Department of Chemistry and Molecular Biology, University of Gothenburg SE-41390 Gothenburg Sweden

## Abstract

The self-assembly of d^8^ transition metal complexes is essential for the development of optoelectronic and sensing materials with superior photofunctional properties. However, detailed insight into the electronic delocalization of excited states across multiple molecules, particularly in comparing 5d^8^ (Pt(ii)) and 4d^8^ (Pd(ii)) systems, remains ambiguous but important. In this study, we have successfully evaluated the differences in the excited-state delocalization and thermal responses of self-assembled Pt(ii) and Pd(ii) complexes. Although the complexes presented herein, K[M(CN)_2_(dFppy)]·H_2_O (M = Pt or Pd, dFppy = 2-(4,6-difluorophenyl)pyridinate), are crystallographically isomorphous with similarly short metal⋯metal contacts, only the Pt(ii) complex exhibited thermal equilibria between delocalized excited states, resulting in a drastic thermochromic luminescence with a red-shift of greater than 100 nm. In contrast, the dimeric localized emission from the Pd(ii) complex showed a significant increase in the quantum yield upon cooling, approaching almost unity.

## Abstract 2

The emissions of isomorphous Pt(ii) and Pd(ii) one-dimensional chains exhibited distinct thermal responses, revealing the limiting factor for extending the excited-state delocalization across multiple molecules through metallophilic interactions.

## Graphical abstract

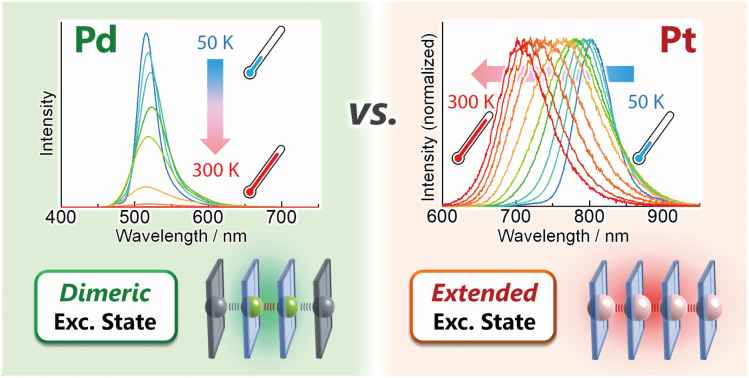

## Introduction

Non-covalent interactions between d^8^ or d^10^ metal ions, referred to as “metallophilic interactions,” play an essential role in a wide range of fields, including supramolecular chemistry, materials chemistry, and photochemistry.^1^ In particular, self-assembled Pt(ii) complexes with Pt⋯Pt interactions have garnered significant interest for a long time^2,3^ following the discovery of a paradigmatic one-dimensional Pt⋯Pt chain, K_2_[Pt(CN)_4_].^4^ Their most notable and unique feature is their coloration and luminescence depending on the strength of the Pt⋯Pt interactions, enabling their application in stimuli-sensing systems.^2^ Such features are attributed to the delocalization of molecular orbitals and excited states over multiple molecules within the Pt⋯Pt chains.^5^ Furthermore, exciton delocalization across multiple Pt(ii) complexes within the self-assembled chains has recently been highlighted as the key to achieving highly efficient near-infrared (NIR) luminescence by suppressing structural displacement during excitation.^3^ Thus, a better understanding of the electronically delocalized excited states upon assembly of Pt(ii) complexes with metallophilic interactions is central to designing highly photofunctional materials for optoelectronic and sensing applications.

**Fig. 1:**
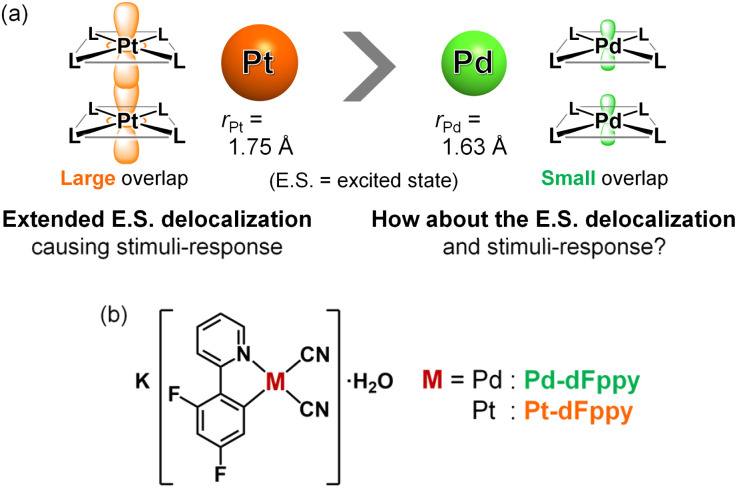
(a) Schematic illustration depicting metallophilic interactions in Pt(ii) and Pd(ii) complexes. (b) Molecular structures of the complexes investigated in this study.

To gain a precise understanding of the intermolecularly delocalized excited states within a one-dimensional metal chain, exploring the relationship between the characters of metal ions and their excited states based on metallophilic interactions is crucial. For square-planar d^8^ metal ions (*i.e.*, Pt(ii) and Pd(ii)), close contacts between metal ions typically results in the formation of bonding dσ(M⋯M) and antibonding dσ*(M⋯M) orbitals *via* the intermolecular overlap of occupied d_*z*_^2^ orbitals (Scheme S1;† M = Pt, Pd). If the metal ion is coordinated by ligands with low-energy π* orbitals, charge transfer transitions occur between dσ*(M⋯M) and π* orbitals, which is named as the metal–metal-to-ligand charge transfer (MMLCT) transitions. As mentioned above, the ^1^MMLCT absorption and ^3^MMLCT emission in Pt(ii) complexes has been widely studied due to facile overlap between the large 5d_*z*_^2^ orbitals of Pt (Fig. 1(a)).^2,3^ In contrast, despite having the same d^8^ electronic configuration, surprisingly few examples of ^3^MMLCT emissions from self-assembled Pd(ii) complexes exist,^6–9^ and thus, their ^3^MMLCT excited-state photophysics remain largely unexplored. This is attributed to the smaller and less diffuse 4d_*z*_^2^ orbital of Pd relative to the 5d_*z*_^2^ orbital of Pt, rendering intermolecular interactions between Pd centers unfavorable (Fig. 1(a)), which is consistent with the smaller van der Waals radius of Pd (*r*_Pd_ = 1.63 Å) relative to Pt (*r*_Pt_ = 1.75 Å).^10^ Here, one fundamental point of interest arises regarding the differences in excited state electronic delocalization between the smaller 4d_*z*_^2^ orbitals and the larger 5d_*z*_^2^ orbitals. Such comparisons between Pd(ii) and Pt(ii) should provide crucial, fundamental insight into the metallophilic interactions in coordination complexes and their intermolecularly delocalized ^3^MMLCT excited states. However, to the best of our knowledge, detailed investigations into the differences in the extension of delocalized excited states between Pd(ii) and Pt(ii) complexes have yet to be reported.

Here we have succeeded in revealing the differences in electronic delocalization of the ^3^MMLCT excited state in Pd⋯Pd and Pt⋯Pt interactions in self-assembled Pd(ii) and Pt(ii) complexes, resulting in differences in their thermal responses. To compare the MMLCT excited states between Pd(ii) and Pt(ii), we have investigated the photophysical properties of isomorphous Pd(ii)/Pt(ii) complexes, K[M(CN)_2_(dFppy)]·H_2_O (M = Pd (Pd-dFppy) or Pt (Pt-dFppy) in Fig. 1(b); dFppy = 2-(4,6-difluorophenyl)pyridinate) which exhibit one-dimensional metallophilic interactions. Although both Pd-dFppy and Pt-dFppy display ^3^MMLCT emissions based on metallophilic interactions, only Pt-dFppy exhibits a drastic thermochromic shift in the emission spectrum. In contrast, the ^3^MMLCT emission of Pd-dFppy shows a significant increase in the quantum yield upon cooling, approaching almost unity, at nearly the same emission wavelength. Detailed variable temperature studies revealed that these differences in emission properties stem from disparities in the delocalization of electron density in ^3^MMLCT excited states through metallophilic interactions.

## Results and discussion

### Crystal structures

Single crystals of Pd-dFppy and Pt-dFppy suitable for X-ray crystallography were obtained through recrystallization from non-dehydrated MeCN/^*t*^BuOMe. Pd-dFppy was isolated as yellow needle-like crystals, whereas Pt-dFppy was obtained as dark green needle-like crystals. The phase purity of bulk polycrystalline samples after recrystallization was confirmed by elemental analysis, powder X-ray diffraction, and thermogravimetric analysis (Fig. S1 and S2 and Experimental details in the ESI†).

**Fig. 2:**
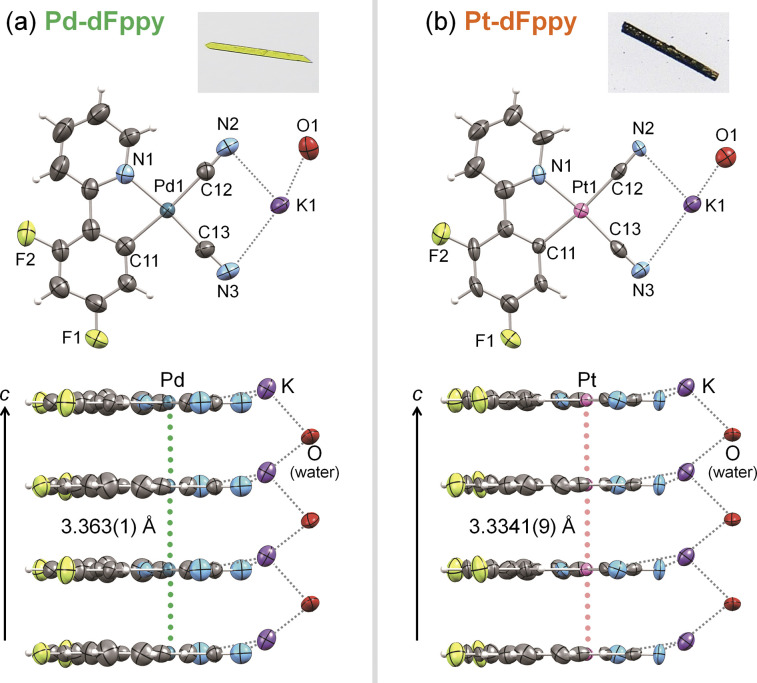
Photographs of crystals (top) and ORTEP drawings of molecular (middle) and stacking structures (bottom) of (a) Pd-dFppy and (b) Pt-dFppy at 240 K. Thermal ellipsoids are displayed at the 50% probability level.

The crystal structures of Pd-dFppy and Pt-dFppy were investigated by single crystal X-ray diffraction at 240 K, as shown in Fig. 2 and S3.†Pd-dFppy and Pt-dFppy are isomorphous and comprise a crystallographically independent [M(CN)_2_(dFppy)]^−^ (M = Pd or Pt) ion, one K^+^ ion, and one water molecule coordinated to K^+^. In both complexes, the Pd and Pt centers adopt square-planar geometry coordinated by a dFppy ligand and two CN^−^ ligands. The [M(CN)_2_(dFppy)]^−^ anions are stacked one-dimensionally along the *c*-axis in a parallel manner in both crystals (Fig. S3(ii)†), where the electrostatic repulsion between [M(CN)_2_(dFppy)]^−^ anions is neutralized by K^+^. This packing arrangement resembles that of K[Pt(CN)_2_(ppy)]·H_2_O (ppy = 2-phenylpyridinate).^11^ Importantly, both Pd-dFppy and Pt-dFppy exhibit short intermolecular metal···metal contacts between the stacked molecules. For Pd-dFppy, the Pd⋯Pd distance (*d*_Pd⋯Pd_ = 3.363(1) Å) was found to be slightly longer than twice the van der Waals radius of Pd (2*r*_Pd_ = 3.26 Å). However, according to previous reports, this Pd⋯Pd distance is short enough to form weak Pd⋯Pd interactions.^7,9^ On the other hand, the intermolecular Pt⋯Pt distance in Pt-dFppy (*d*_Pt⋯Pt_ = 3.3341(9) Å) is significantly shorter than 2*r*_Pt_ (3.50 Å), revealing the presence of strong intermolecular Pt⋯Pt interactions. Given the proximity of M⋯M distances in the solid-state structures of these complexes, assembly-induced MMLCT absorptions and emissions based on metallophilic interactions were expected.

### Absorption and emission properties

**Fig. 3:**
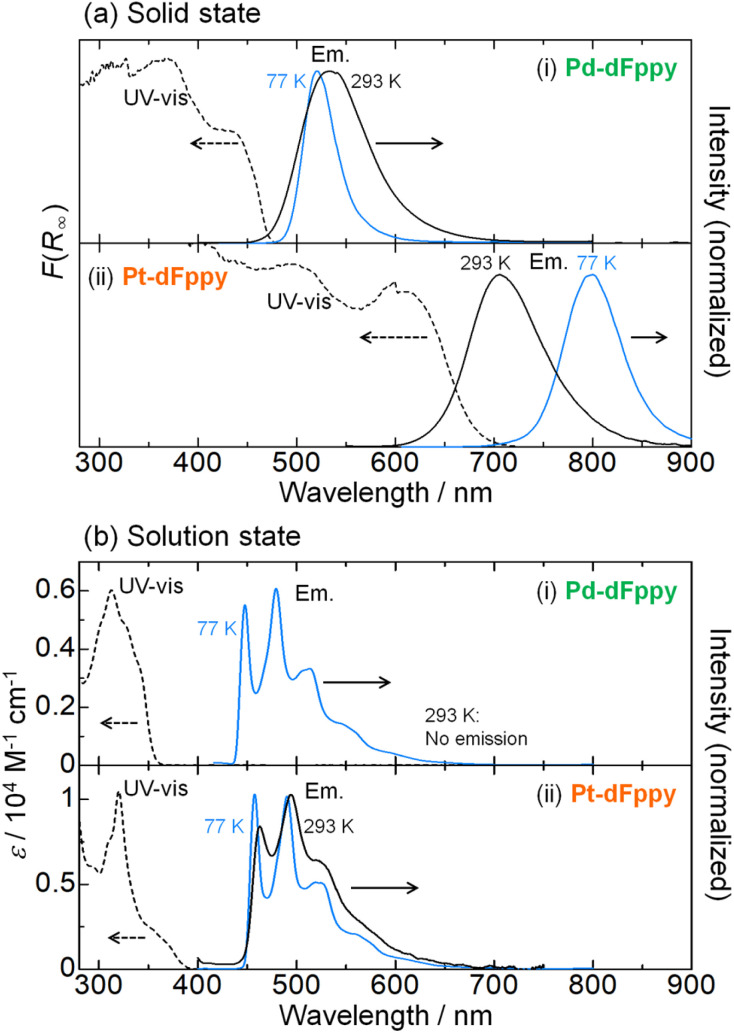
UV-vis absorption (dashed lines) and emission (solid lines) spectra of (i) Pd-dFppy and (ii) Pt-dFppy in (a) the solid state and (b) the solution state (MeOH/EtOH (v/v = 1/1); 5.0 × 10^−5^ M) at 293 K (black) and 77 K (blue) (*λ*_ex_ = 350 nm for solution, 400 nm for Pd-dFppy in the solid state, and 500 nm for Pt-dFppy in the solid state).

The UV-vis absorption spectra of Pd-dFppy and Pt-dFppy were measured both in the solid state and in solution to evaluate the influence of M⋯M interactions on their electronic structures. Consistent with the colors of the crystals, Pd-dFppy and Pt-dFppy exhibit broad absorption bands at approximately 430 and 610 nm in the solid state, respectively (dashed lines in Fig. 3(a)). These absorption bands are assigned to the ^1^MMLCT transition arising from metallophilic interactions, as neither complex shows any intense absorption bands in the visible region in the discrete state (*i.e.*, in solution; dashed lines in Fig. 3(b)). The absorption bands observed below 400 nm in the solution state can be assigned to the singlet metal-to-ligand charge transfer (^1^MLCT) transition without metallophilic interactions for both complexes (Fig. S4–S6†).^12^ In addition, no new absorption bands are observed for either complex at concentrations up to 100 μM (Fig. S4(b)†), indicating that self-assembly of the complexes in solution is negligible in this concentration range. On the other hand, the addition of a poor solvent to the solutions of complexes resulted in the appearance of characteristic ^1^MMLCT absorption bands (Fig. S7†), further confirming the importance of the assembly of complexes to exhibit MMLCT transitions.

Both Pd-dFppy and Pt-dFppy exhibit characteristic ^3^MMLCT emissions in the solid state (black solid lines in Fig. 3(a)) corresponding to the observed absorptions. As expected, Pd-dFppy exhibits weak green emission and displays a broad structureless emission band (*λ*_max_ = 534 nm) at 293 K. Conversely, Pt-dFppy displays a similarly broad structureless emission band, but its emission maximum (*λ*_max_ = 705 nm) is observed at a significantly longer wavelength than that of Pd-dFppy, resulting in a deep red emission color at 293 K. An almost identical ^3^MMLCT emission band was similarly observed for the aggregate prepared by adding a poor solvent to the solution (Fig. S7(b)†). The red-shifted ^3^MMLCT emission of Pt-dFppy compared to that of Pd-dFppy suggests the energy of the dσ*(Pt⋯Pt) orbital in Pt-dFppy is much higher than that of the dσ*(Pd⋯Pd) orbital in Pd-dFppy. In addition, the large radiative rate constant (*k*_r_) of Pt-dFppy (6.1 × 10^5^ s^−1^; Table 1) is typical of the ^3^MMLCT emission of Pt(ii) complexes.^13,14^ In contrast to the solid state data, both Pd-dFppy and Pt-dFppy exhibit approximately the same emission spectra in dilute solutions due to the negligible metallophilic interactions (Fig. 3(b)). The vibronically structured emission bands in the solution state indicate that the emissions originate from a ligand-centered ^3^ππ* excited state of discrete monomer complexes, consistent with the previous report on (Bu_4_N)[Pt(CN)_2_(dFppy)].^12^ Computational studies further support this assignment (Fig. S8†). Overall, the significant differences in electronic transitions between the solid and solution states undoubtedly support the presence of assembly-induced ^3^MMLCT excited states arising from metallophilic interactions in both Pt-dFppy and Pd-dFppy. The differences in electronic transitions between the solid and solution states have also been confirmed by the corresponding excitation spectra (Fig. S9†).

**Table 1:** Photophysical data of Pd-dFppy and Pt-dFppy in (a) the solid state and (b) the solution state (MeOH/EtOH (v/v = 1/1); 5.0 × 10^−5^ M; N_2_-saturated)

Complex	(a) Solid state							(b) Solution state		
	*T*/K	*λ*_max_ a/nm	*Φ* b	*τ* c/μs (*A*^d^)	*τ*_av_ e/μs	*k*_r_ f/s^−1^	*k*_nr_ g/s^−1^	*T*/K	*λ*_max_ a/nm	*Φ* b
Pd-dFppy	293	534	<0.01	0.0300 (0.668), 0.168 (0.332)	0.132	—	—	293	—h	—h
	77	520	0.92	8.61 (0.476), 16.5 (0.524)	14.0	6.6 × 10^4^	5.7 × 10^3^	77	448, 479, 513	0.46
Pt-dFppy	293	705	0.01	0.00819 (0.658), 0.0222 (0.342)	0.0163	6.1 × 10^5^	6.1 × 10^7^	293	463, 494, 521^i^	<0.01^i^
	77	800	0.16	0.166 (0.473), 1.03 (0.526)	0.916	1.8 × 10^5^	9.2 × 10^5^	77	458, 490, 519^i^	0.72

a Emission maxima.

b Emission quantum yields.

c Emission lifetimes.

d Pre-exponential factors.

e Averaged emission lifetimes.

f Radiative rate constants, *k*_r_ = *Φ*/*τ*_av_.

g Nonradiative rate constants, *k*_nr_ = *k*_r_(1 − *Φ*)/*Φ*.

h Non-emissive.

i Consistent with literature values for (Bu_4_N)[Pt(CN)_2_(dFppy)].^12^

The most notable difference between Pd-dFppy and Pt-dFppy is their temperature-dependent behavior (blue lines in Fig. 3(a)). The ^3^MMLCT emission energies of typical one-dimensional Pt(ii) complexes have been reported to exhibit a red-shift with decreasing temperature,^15^ and indeed, the emission band of Pt-dFppy shifted into the NIR region (*λ*_max_ = 800 nm) upon cooling to 77 K. Conversely, the shift of the emission band of Pd-dFppy at 77 K was not significant (*λ*_max_ = 520 nm), indicating the non-thermochromic nature of Pd-dFppy. Instead, the emission intensity of Pd-dFppy was drastically enhanced to approximate unity upon cooling (*Φ* < 0.01 at 293 K to *Φ* = 0.92 at 77 K; Table 1).

### Computational studies

For an in-depth investigation into the nature of the intermolecular interactions and MMLCT transitions, computational studies were performed for cluster model systems M2, M4, and M6 (M*n* = stacked clusters of the Pd or Pt complexes with *n* layers) based on the structures of Pd-dFppy and Pt-dFppy. The ground-state structures of the cluster models (Fig. S10†) were fully optimized using the two-layer ONIOM method.^16^

We used the optimized Pd2 and Pt2 systems for energy decomposition analyses (EDA).^17^ Numerical data from the EDA are summarized in Table 2. The computed interaction energies (Δ*E*_int_) in the Pd2 and Pt2 systems are −102.0 and −107.6 kcal mol^−1^, respectively. Hence, the Pt2 system exhibits relatively strong interactions. The Δ*E*_int_ term was then decomposed into Pauli repulsion (Δ*E*_Pauli_), electrostatic attraction (Δ*E*_elstat_), orbital interactions (Δ*E*_orb_), and dispersion interactions (Δ*E*_disp_). Notably, the computed Δ*E*_Pauli_ for the Pd2 system (47.8 kcal mol^−1^) is relatively weak compared to the Pt2 system (53.7 kcal mol^−1^), reflecting a less overlap between the 4d_*z*_^2^ orbitals in Pd2 than the 5d_*z*_^2^ orbitals in Pt2. The computed Δ*E*_orb_ for the Pd2 system (−83.6 kcal mol^−1^) is similar to that of the Pt2 system (−82.7 kcal mol^−1^). In addition, Δ*E*_elstat_ for the Pt2 system (−40.1 kcal mol^−1^) is significantly stronger than that of the Pd2 system (−29.5 kcal mol^−1^), which could be a crucial factor in describing the attractive intermolecular interaction between Pt(ii) centers leading to slightly short M⋯M distances compared to the Pd systems.

**Table 2:** Numerical data of EDA (the energies are in kcal mol^−1^)

	Pd2	Pt2
Δ*E*_int_	−102.0	−107.6
Δ*E*_Pauli_	47.8	53.7
Δ*E*_elstat_	−29.5	−40.1
Δ*E*_orb_	−83.6	−82.7
Δ*E*_disp_	−36.8	−38.5

The vertical excitations of the M2, M4, and M6 systems were calculated to evaluate the excited states. The computed excitation wavelengths and the corresponding natural transition orbitals are summarized in Table S1 and Fig. S11.† The highest occupied natural transition orbitals for all systems are located on the M⋯M chain, whereas the lowest unoccupied natural transition orbitals are localized on the dFppy ligand (Fig. S11†), clearly indicating the ^1^MMLCT origin of the absorption bands for both Pd-dFppy and Pt-dFppy. The ^1^MMLCT excitations in the Pd systems followed the order: Pd2 (389 nm) < Pd4 (419 nm) < Pd6 (458 nm), and those of the Pt systems followed the order: Pt2 (426 nm) < Pt4 (468 nm) < Pt6 (568 nm). Thus, compared with the Pd clusters, the computed ^1^MMLCT vertical excitations of the Pt clusters occurred at relatively long wavelengths. Even in the Pd6 system (according to previous reports, this would not be favorable),^5^ the excitation energy (458 nm) was considerably higher than that of Pt6 (568 nm). This tendency is consistent with the experimental observations.

### Thermochromic response

**Fig. 4:**
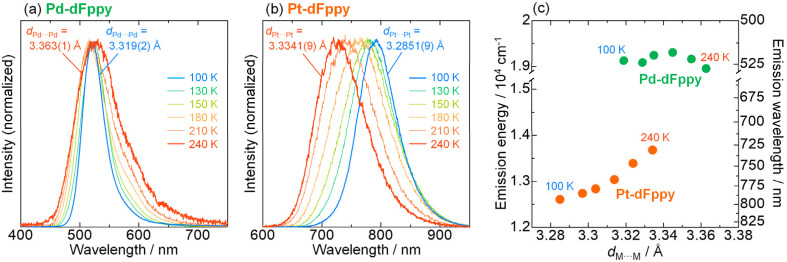
Temperature dependence of the emission spectra of (a) Pd-dFppy (*λ*_ex_ = 400 nm) and (b) Pt-dFppy (*λ*_ex_ = 500 nm) in the solid state, displayed alongside *d*_M⋯M_ lengths at 240 and 100 K. (c) Correlation between the emission maxima and the M⋯M distances (*d*_M⋯M_) of Pd-dFppy (green) and Pt-dFppy (orange).

As mentioned earlier, the most significant distinction between Pd-dFppy and Pt-dFppy is the temperature-dependent emission shift (*i.e.*, thermochromic luminescence). To gain further insight, variable-temperature X-ray structural analyses and emission measurements were performed (Fig. 4 and S12†). No phase transition was observed in the temperature range of 100–240 K for either Pt-dFppy or Pd-dFppy, however, when the temperature was decreased, anisotropic contraction of the crystals in the direction of the *c*-axis was observed (Fig. S12(a)†). Since the [M(CN)_2_(dFppy)]^−^ anions are stacked in the direction of the *c*-axis, both Pt-dFppy and Pd-dFppy showed similar anisotropic contraction in *d*_M⋯M_ lengths (∼0.05 Å; Fig. S12(b)†) when the temperature was decreased from 240 K to 100 K. Such shortening in Pt⋯Pt distance upon cooling has been widely considered to be the cause of the thermochromic shift of the ^3^MMLCT emissions,^5*a*,15^ as observed in Pt-dFppy (Fig. 4(b)). In contrast, despite the similar degree of shortening in the Pd⋯Pd distance, only a small shift in the emission maxima was observed for Pd-dFppy upon cooling (Fig. 4(a)), except for the decrease of the spectral width. Although the ^3^MMLCT emission of Pd-dFppy originates from intermolecular Pd⋯Pd interactions, these results indicate the ^3^MMLCT emission energy in Pd-dFppy is not sensitive to changes in Pd⋯Pd distance (*d*_Pd⋯Pd_), unlike Pt-dFppy (Fig. 4(c)). Similar behavior was observed in the excitation spectra (Fig. S13†), indicating that not only the emission from the ^3^MMLCT state, but also the vertical excitation to the ^1^MMLCT state reflect differences in metallophilic interactions involving Pd and Pt.

**Fig. 5:**
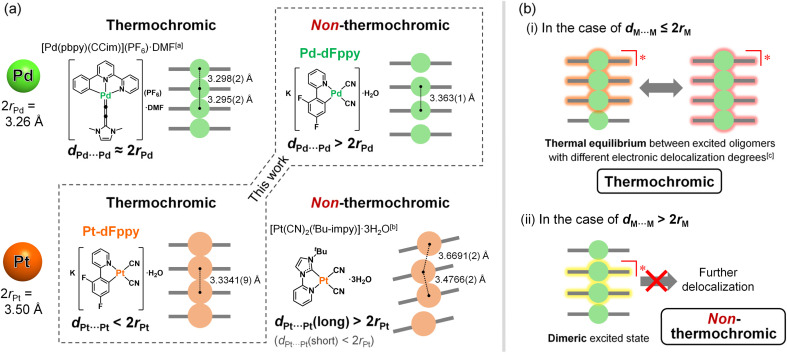
(a) Examples of thermochromic and non-thermochromic Pt(ii) and Pd(ii) complexes with effective metallophilic interactions. (b) Suggested mechanisms of (i) thermochromic and (ii) non-thermochromic ^3^MMCLT emission. [a] Ref. 8a. [b] Ref. 13a. [c] Ref. 5a.

To discuss the relationship between metallophilic interactions and thermochromism, the results reported herein are compared with those of previous reports. Lu *et al.* reported that [Pd(pbpy)(CCim)](PF_6_) (pbpyH = 6-phenyl-2,2′-bipyridine, CCimH^+^ = 2-ethynyl-1,3-dimethyl-1*H*-imidazolium), a complex that exhibits short Pd⋯Pd distances (*d*_Pd⋯Pd_ = 3.295(2) and 3.298(2) Å) comparable to 2*r*_Pd_ (3.26 Å), demonstrates obvious thermochromic ^3^MMLCT emission (Fig. 5(a)).^8*a*^ On the other hand, we recently reported a unique case in which [Pt(CN)_2_(^*t*^Bu-impy)] (^*t*^Bu-impyH^+^ = 1-*tert*-butyl-3-(2-pyridyl)-1*H*-imidazolium; see Fig. 5(a)),^13*a*^ which is a one-dimensional Pt(ii) complex with slightly longer *d*_Pt⋯Pt_ than 2*r*_Pt_, exhibits non-thermochromic ^3^MMLCT emission.^13^ Moreno *et al.* also recently reported that Pt(ii) complex [Pt(dFppy)Cl(CN^*t*^Bu)] with longer Pt⋯Pt distances (3.6876(5) and 3.8176(5) Å) exhibits a smaller thermochromic shift of the ^3^MMLCT emission.^18^ These suggest that a drastic thermochromic shift of the ^3^MMLCT emission occurs if *d*_M⋯M_ is shorter than 2*r*_M_ by a certain margin.

Sakaki *et al.* discussed the thermochromic mechanism for [Pt(CN)_2_(bpy)] (bpy = 2,2′-bipyridine), which forms a typical one-dimensional stacked arrangement with short *d*_Pt⋯Pt_ (3.35 Å at 293 K).^5*a*^ According to their explanation, thermochromic luminescence is mainly caused by thermal equilibrium between the trimerically and tetramerically delocalized ^3^MMLCT excited states, whereby the equilibrium shifts due to changes in *d*_Pt⋯Pt_ caused by changes in temperature (as illustrated in (i) in Fig. 5(b)). Indeed, the full width at half maximum (FWHM) of the emission band of Pt-dFppy showed a two-step temperature dependence: it increased until a temperature of approximately 210 K was reached, then decreased with temperature (Fig. S14(a)†), consistent with the presence of multiple competitive emission components. Based on this explanation, the absence of thermochromism in Pd-dFppy is indicative of a dimeric ^3^MMLCT excited state without thermal equilibrium with further extended oligomeric excited states (as illustrated in (ii) in Fig. 5(b)), as suggested for [Pt(CN)_2_(^*t*^Bu-impy)].^13*a*^ Certainly, the emission band of Pd-dFppy displayed typical thermal broadening without any decrease in FWHM (Fig. S14(b)†), indicating an absence of any thermal change of the emission components. Thus, the above facts suggest that thermal equilibria between delocalized excited states are achieved when *d*_M⋯M_ is sufficiently shorter than 2*r*_M_. Notably, the van der Waals radii of Pd and Pt (*r*_Pd_ and *r*_Pt_) were originally proposed based on the change in absorption of crystals of [M(Hdmg)_2_] (H_2_dmg = dimethylglyoxime) and related derivatives,^10^ but in fact, this absorption band can be assigned to the one-dimensionally delocalized dσ*(M⋯M) → pσ(M⋯M)-type transitions of the M⋯M chain.^19^ In other words, traditional 2*r*_Pd_ and 2*r*_Pt_ distances mean the M⋯M distances sufficient to facilitate the extension of the electronically delocalized ^1^MMLCT excited state (as well as the ^3^MMLCT state) across multiple molecules in a one-dimensional chain.^20^

The difference in the electronic delocalization of the ^3^MMLCT excited state between the Pd(ii) and Pt(ii) complexes has been further supported by the energy difference in their emissions. Recently, Strassert *et al.* compared the photophysical properties of crystallographically isomorphous Pd(ii) and Pt(ii) complexes PdL and PtL (H_2_L = *N*,*N*-di-[6-(2,6-difluoropyridin-3-yl)-4-methoxypyridin-2-yl]-4-hexylaniline) exhibiting dimeric structures with similar M⋯M distance (3.5025(8) Å for Pd, 3.4748(7) Å for Pt).^9*a*^ The shift in the dimeric ^3^MMLCT emission of PtL relative to that of PdL was found to be approximately 2.0 × 10^3^ cm^−1^ (Scheme S2†). For the present complexes, the calculated vertical excitation energy of the dimeric model of Pt-dFppy (*i.e.*, Pt2) is similarly red-shifted by 2.2 × 10^3^ cm^−1^ compared to that of the dimeric model of Pd-dFppy (*i.e.*, Pd2) (see “Computational studies”; Scheme S2†), if both Pd-dFppy and Pt-dFppy form a dimeric excited state. However, the experimental ^3^MMLCT emission of Pt-dFppy was red-shifted by 4.5 × 10^3^ cm^−1^ compared to Pd-dFppy (Fig. 3(a)). These results reveal that Pt-dFppy likely forms excited oligomers with more delocalized electron density than Pd-dFppy due shorter *d*_Pt⋯Pt_ than 2*r*_Pt_, resulting in thermochromism *via* a thermal equilibrium between excited oligomers ((i) in Fig. 5(b)).^5^ In contrast, when *d*_M⋯M_ is slightly longer than 2*r*_M_, such an extension in electron density delocalization is negligible. Therefore, the observed ^3^MMCLT emission for Pd-dFppy is attributed to a dimeric excited state, leading to a high-energy emission with non-thermochromism ((ii) in Fig. 5(b)).^13*a*^

### Thermal quenching behavior

**Fig. 6:**
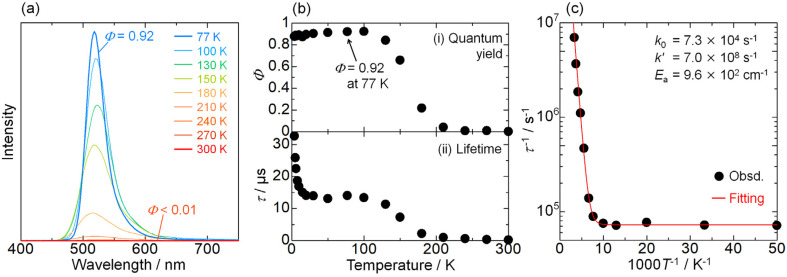
(a) Temperature dependence of the non-normalized emission spectrum; (b) (i) emission quantum yield, and (ii) average emission lifetime of Pd-dFppy (*λ*_ex_ = 400 nm) in the solid state. The emission quantum yield values at temperatures other than 77 K were determined by measuring the relative peak areas of the emission bands and were set in relation to the value for absolute emission quantum yield at 77 K (*Φ* = 0.92). (c) Plot of the decay rate constant (*τ*^−1^) as a function of temperature (20–300 K). The red line indicates the fitted curves based on eqn (1), and the resulting fitting parameters are shown in the inset.

Instead of the non-thermochromism, Pd-dFppy shows a drastic increase in emission intensity to almost unity (*Φ* = 0.92 at 77 K) upon cooling (Fig. 6(a)). As shown in Fig. 6(b), the emission quantum yield for Pd-dFppy increased rapidly from 210 to 100 K, and the emission lifetime increased similarly in this temperature range. Therefore, the increase in emission lifetime is caused by the suppression of thermally activated nonradiative decay. This behavior could be characteristic of the ^3^MMLCT emission of Pd-dFppy, since such drastic and rapid thermal quenching has not been observed in Pt-dFppy (Fig. S15†). To validate this thermal quenching behavior, the emission lifetime data in the range of 20–300 K was analyzed using the following Arrhenius-type eqn (1) (Fig. 6(c)):1
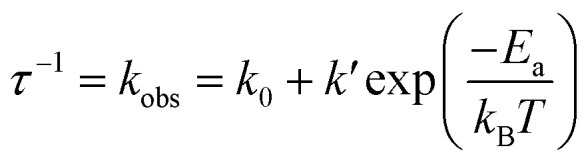
where *k*_obs_ is the apparent decay rate constant, *k*_0_ is the temperature-independent decay rate constant in this temperature region (*i.e.*, 20–300 K), *k*′ is the frequency factor for the thermally activated nonradiative decay, *E*_a_ is the activation barrier, and *k*_B_ is the Boltzmann constant. The optimized fitting parameters are summarized in the inset of Fig. 6(c). The optimized frequency factor for thermal deactivation (*k*′ = 7.0 × 10^8^ s^−1^) is significantly smaller than the typical value for nonradiative decay from the metal-centered ^3^dd excited state (above 10^10^ s^−1^),^21^ suggestive of deactivation through a non-emissive triplet state with large structural distortion.^22,23^ The small activation barrier (*E*_a_ = 9.6 × 10^2^ cm^−1^) clearly indicates substantial ease of thermal deactivation of the ^3^MMLCT state. Although the details of the nonradiative process have not been fully clarified, deactivation could be attributed to insufficient delocalization of the excited state. As highlighted by Chi and Chou *et al.*, molecular displacement during excitation is drastically suppressed by exciton delocalization across multiple molecules.^3*a*,*d*,*e*^ Because the ^3^MMLCT excited state of Pd-dFppy is only delocalized over two adjacent molecules, it undergoes a relatively large thermal deformation to a more stable and highly distorted non-emissive conformation. Furthermore, because of the relatively smaller *k*_r_ value for Pd-dFppy compared to Pt-dFppy, the luminescence of Pd-dFppy is affected significantly by this structural distortion, resulting in drastic thermal quenching behavior.

In contrast, the change in emission lifetime below 20 K was not accompanied by a change in the emission quantum yield for Pd-dFppy (Fig. 6(b)). Therefore, the temperature dependence observed below 20 K was not due to thermal deactivation, but mainly due to the zero-field splitting (ZFS) of the T_1_ (*i.e.*, ^3^MMLCT) state, as described below.

### Temperature dependence at very low temperatures

**Fig. 7:**
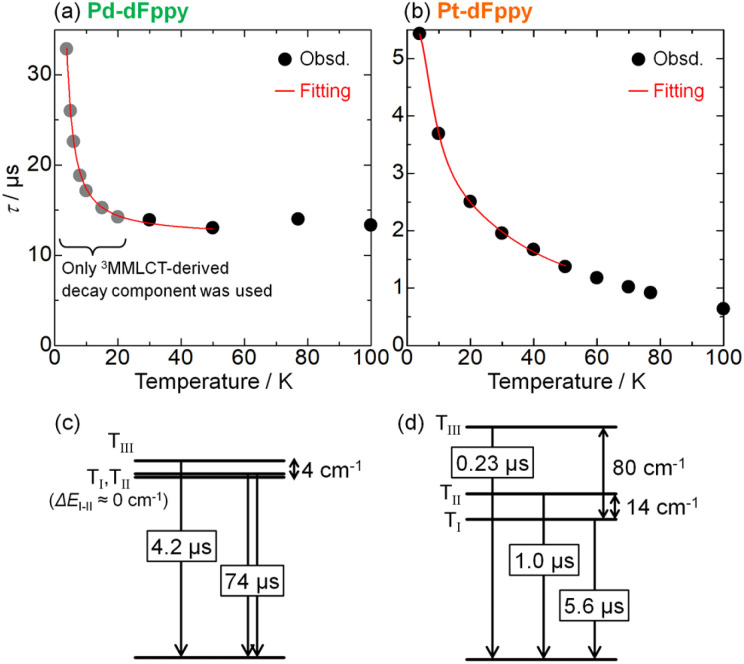
Temperature dependence of emission lifetimes of (a) Pd-dFppy and (b) Pt-dFppy. Red lines represent the fitted curves based on eqn (2). Schematic energy diagrams showing the optimized fitting parameters for (c) Pd-dFppy and (d) Pt-dFppy (see also Table S2†).

Finally, we evaluated the ZFS values of the T_1_ states of Pd-dFppy and Pt-dFppy, which reflect the degree of spin–orbit coupling.^24^ For this purpose, the emission lifetime data at very low temperatures (<50 K) were analyzed using eqn (2) (ref. 25) (Fig. 7).2
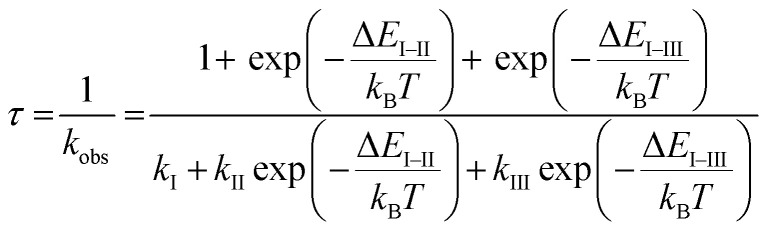
where *k*_*n*_ (*n* = I, II, and III) are the decay rate constants of individual sublevels of the T_1_ state, and Δ*E*_I–II_ and Δ*E*_I–III_ are the energy separations between the sublevels. For Pt-dFppy (Fig. 7(b)), the data between 4 and 50 K were used for fitting because the emission energies became almost constant in this temperature regime, whereas the emission band was shifted in the high temperature range (Fig. 4(b and c) and S14†). As a result, the optimized ZFS value (Δ*E*_I–III_) of 80 cm^−1^ (Fig. 7(d)) is nearly comparable to those of other ^3^MMLCT states of self-assembled Pt(ii) complexes (Scheme S3†)^13*a*^ and slightly less than that of a discrete dinuclear Pt(ii) complex.^26^

The analysis for Pd-dFppy was slightly more complicated compared to Pt-dFppy. Below 20 K, Pd-dFppy exhibited competitive emission between ^3^MMLCT and slightly higher-lying ^3^ππ* states, as indicated by the global fitting analysis of emission decays (Fig. S16†). Therefore, at temperatures below 20 K, only the ^3^MMLCT-derived decay component was used for fitting the data (Fig. 7(a)). During the fitting, the Δ*E*_I–II_ value was found to be very small (<1 cm^−1^); thus, this value was approximated as 0 to optimize the other fitting parameters as shown in Fig. 7(c). The optimized ZFS value (Δ*E*_I–III_) of the T_1_ state of Pd-dFppy was found to be only ∼4 cm^−1^, indicating weaker spin–orbit coupling for Pd compared to Pt. This ZFS value is comparable to those of the triplet metal-to-ligand charge-transfer (^3^MLCT) states of Cu(i) complexes, which are known to exhibit weaker spin–orbit coupling.^27^ Such a small ZFS for Pd-dFppy is reasonable considering a previous report indicating the ZFS of a ^3^ππ*/^3^MLCT state of [Pd(thpy)_2_] (0.0962 cm^−1^; thpy = 2-thyenylpyridinate), a discrete Pd(ii) complex, is much smaller than that of [Pt(thpy)_2_] (16 cm^−1^).^28^ However, this is the first example of ZFS of the ^3^MMLCT state of a self-assembled Pd(ii) complex.

## Conclusions

**Fig. 8:**
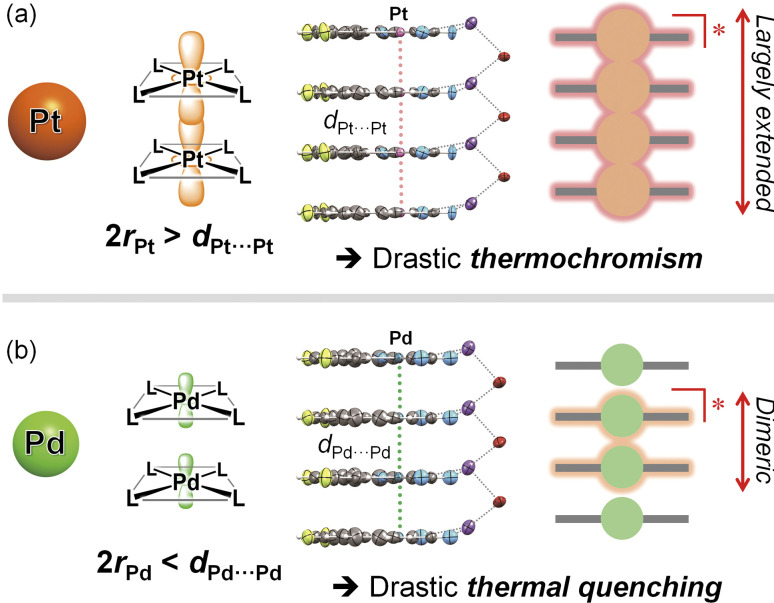
Schematic illustrations of the differences in electronic delocalization in the excited state, and the resulting thermal response for (a) Pt-dFppy and (b) Pd-dFppy.

In conclusion, we have successfully evaluated the differences in the electronic delocalization of the ^3^MMLCT excited state, which drastically governs thermal responsiveness (Fig. 8). Isomorphous Pd(ii) and Pt(ii) complexes that exhibit a one-dimensional stacking arrangement with similar M⋯M distances, Pd-dFppy and Pt-dFppy, show green and deep red ^3^MMLCT emissions, respectively, which depend on the strength of metallophilic interactions. Despite their structural similarity, only Pt-dFppy displayed obvious thermochromic luminescence, whereas Pd-dFppy demonstrated drastic thermal quenching behavior. A comprehensive comparison with present and previous studies indicated that the ^3^MMLCT excited state should be oligomerically delocalized through the metallophilic interactions (Fig. 8(a)) if the M⋯M distance (*d*_M⋯M_) is sufficiently shorter than twice the van der Waals radii (2*r*_M_). Thus, Pt-dFppy, which has a sufficiently short *d*_Pt⋯Pt_ relative to 2*r*_Pt_, showed a large thermochromic shift due to thermal equilibrium between excited oligomers. Although *d*_Pd⋯Pd_ is slightly longer than 2*r*_Pd_, Pd-dFppy still showed ^3^MMLCT emission, given the van der Waals radii of Pt and Pd are still ambiguous.^10,29^ However, the electron density of the ^3^MMLCT excited state of Pd-dFppy is localized between two neighboring molecules without thermal equilibrium (Fig. 8(b)), resulting in an emission that shows drastic thermal quenching owing to structural deformation during excitation. Despite the recent revelation of the solution-state equilibria of excited oligomers of Pt(ii) complexes,^30^ this work provides a crucial experimental evidence for the thermal equilibria of intermolecularly delocalized ^3^MMLCT excited states within one-dimensional metal chains in the solid state, resulting in a thermochromic response. While ^3^MMLCT-emissive materials have been widely studied, the results presented herein shed light on the limiting factor for extending the degree of delocalization of ^3^MMLCT excited states over multiple molecules through metallophilic interactions for the first time, providing insight for controlling the functionalities of molecular-based materials.

## Data availability

All the data supporting this study are included in the main text and the ESI.†

## Author contributions

M. Y. and M. K. conceived and directed the project. T. S. and K. S. performed the synthesis, characterizations, and all measurements. D. S. and A. K. supported experiments, and evaluated and validated the research. J. T., S. H., and A. M. contributed to the variable-temperature emission measurements. N. M. L. and W. M. C. S. conducted the computational studies. M. Y., W. M. C. S., and M. K. co-wrote the manuscript. All authors discussed the results, and reviewed and approved the final version of the manuscript.

## Conflicts of interest

There are no conflicts of interest to declare.

## Supplementary Material

SC-OLF-D4SC04497E-s001

SC-OLF-D4SC04497E-s002
