# Supplementary material for: Thermo-responsive emission induced by different delocalized excited-states in isomorphous Pd(ii) and Pt(ii) one-dimensional chains
Source: Chem Sci. 2024 Aug 13;15(35):14497–505. doi: 10.1039/d4sc04497e (PMC11333949; doi:10.1039/d4sc04497e)
Supplement: SC-015-D4SC04497E-s001 [file SC-015-D4SC04497E-s001.pdf]

Supplementary Information (SI) for Chemical Science.
This journal is © The Royal Society of Chemistry 2024

Supporting Information for Thermo-responsive emission induced by different delocalized excited-states in isomorphous Pd(II) and Pt(II) one-dimensional chains

Tomoya Saito,[a] Masaki Yoshida,*[b] Kaito Segawa,[b] Daisuke Saito,[a,b] Junichi Takayama,[c] Satoshi Hiura,[c] Akihiro Murayama,[c] Nishshanka M. Lakshan,[d] W. M. C. Sameera,[d,e] Atsushi Kobayashi [a] and Masako Kato *[b]

[a] Department of Chemistry, Faculty of Science, Hokkaido University,
North-10 West-8, Kita-ku, Sapporo, Hokkaido 060-0810, Japan.

[b] Department of Applied Chemistry for Environment, School of Biological and Environmental Sciences, Kwansei Gakuin University,
1 Gakuen-Uegahara, Sanda, Hyogo 669-1330, Japan.

[c] Faculty of Information Science and Technology, Hokkaido University,
North-14 West-9, Kita-ku, Sapporo, Hokkaido 060-0814 Japan.

[d] Department of Chemistry, University of Colombo,
Cumarathunga Munidasa Mawatha, Colombo 00300, Sri Lanka.

[e] Department of Chemistry and Molecular Biology, University of Gothenburg,
SE-412 96 Gothenburg, Sweden.

E-mail: masaki.yoshida@kwansei.ac.jp, katom@kwansei.ac.jp

Table of Contents

Experimental Details

**Scheme S1**	Schematic MO diagram for Pt(II) or Pd(II) complexes with the M···M metallophilic interaction.
**Scheme S2**	Stacking modes and emission maxima of isostructural Pd(II) and Pt(II) complexes in this study and the previous report.
**Scheme S3**	ZFS values of ^{3}MMLCT states of self-assembled Pt(II) complexes.
**Figs. S1-S2**	PXRD and TG data of **Pd-dFppy** and **Pt-dFppy**.
**Fig. S3**	Packing structures of **Pd-dFppy** and **Pt-dFppy**.
**Fig. S4**	UV-vis absorption spectra of complexes and the ligand in MeOH.
**Figs. S5-S6**	Kohn-Sham frontier orbitals and NTOs of the optimized monomers in the S_0 states.
**Fig. S7**	Aggregation behavior of **Pd-dFppy** and **Pt-dFppy**.
**Fig. S8**	Spin density plots of the optimized monomers in the T_1 states.
**Fig. S9**	Excitation spectra of **Pd-dFppy** and **Pt-dFppy** in the solid state and in solution.
**Fig. S10**	Cluster models of M2, M4, and M6 systems.
**Fig. S11**	NTOs for the ^{1}MMLCT vertical transitions of the optimized M2, M4, and M6 systems.
**Fig. S12**	Temperature dependence of the cell lengths and the M···M distances.
**Fig. S13**	Temperature dependence of the excitation spectra of **Pd-dFppy** and **Pt-dFppy**.
**Fig. S14**	Temperature dependence of the full width at half maxima and the emission maxima of **Pd-dFppy** and **Pt-dFppy**.
**Fig. S15**	Temperature dependence of the emission quantum yield of **Pt-dFppy**.
**Fig. S16**	Emission behavior of **Pd-dFppy** at 3.8 K.
**Figs. S17-S19**	^{1}H NMR, ^{13}C NMR, and ESI-MS spectra of **Pd-dFppy** and **Pt-dFppy**.
**Figs. S20-S25**	Kohn-Sham frontier orbitals of the S_0-optimized M2, M4, and M6 systems.
**Table S1**	Important parameters of the DFT/TDDFT results for the M2, M4, and M6 systems.
**Table S2**	Photophysical data of **Pd-dFppy** and **Pt-dFppy** in the solid state.
**Tables S3-S4**	Fitting results for emission decay curves of **Pd-dFppy** and **Pt-dFppy**.
**Table S5**	Crystal parameters and refinement data for **Pd-dFppy** and **Pt-dFppy** at 240 K.
**Table S6**	Selected interatomic distances and angles.
**Tables S7-S8**	Crystal parameters and refinement data at various temperatures during 100-210 K.
**Tables S9-S20**	Cartesian coordinates for the optimized structures.

References

Experimental Details

Materials.

Caution! *Although we experienced no difficulties, all the chemicals used in this study are potentially harmful and should be used in small quantities and handled with care in a fume hood.*

$Na_2[PdCl_4]\cdot 3H_2O$, $AgClO_4$, KCN, methanol, and ^{t}BuOMe were purchased from FUJIFILM Wako Pure Chemical Co. 2-(2,4-Difluorophenyl)pyridine (HdFppy) was purchased from Tokyo Chemical Industry Co., Ltd. Acetonitrile was purchased from Kanto Chemical Co., Inc. Et_2O was purchased from JUNSEI CHEMICAL Co., Ltd. $K[Pt(CN)_2(dFppy)]\cdot H_2O$ (**Pt-dFppy**) was synthesized according to the literature method.[S1]

Synthesis of $[Pd(dFppy)(MeCN)_2](ClO_4)$. A mixture of $Na_2[PdCl_4]\cdot 3H_2O$ (349 mg, 1.00 mmol) and HdFppy (182 µL, 1.20 mmol) in methanol (4 mL) was stirred at 293 K under N_2 atmosphere for 24 h. The yellow precipitate was collected by filtration, washed with methanol and Et_2O, and dried in vacuo to afford the precursor $[Pd_2(\mu\text{-}Cl)_2(dFppy)_2]$. Yield, 244 mg (0.368 mmol, 73%). Then, a mixture of $[Pd_2(\mu\text{-}Cl)_2(dFppy)_2]$ (175 mg, 0.2640 mmol) and $AgClO_4$ (113 mg, 0.590 mmol) in acetonitrile (50 mL) was stirred at 293 K in the dark for 2 h followed by filtration for the removal of AgCl. The filtrate was concentrated by evaporation under reduced pressure followed by the addition of excess Et_2O. The resulting pale-yellow precipitate was collected by filtration, washed with Et_2O, and dried in vacuo. Yield, 219 mg (0.458 mmol, 86%); ^{1}H NMR (400 MHz, DMSO-d_6): δ = 8.45 (d, J = 5.5 Hz, 1H), 8.21-8.14 (m, 1H), 8.01 (d, J = 8.2 Hz, 1H), 7.49 (t, J = 6.6 Hz, 1H), 7.19-7.10 (m, 1H), 6.8 (d, J = 7.7 Hz, 1H). This complex can be used in the next synthesis step without further purification.

Synthesis of $K[Pd(CN)_2(dFppy)]\cdot H_2O$ (Pd-dFppy). A solution of $[Pd(dFppy)(MeCN)_2](ClO_4)$ (120 mg, 0.251 mmol) and KCN (38.1 mg, 0.585 mmol) in methanol (6 mL) was stirred at 293 K for 1 h followed by filtration for the removal of $KClO_4$. The filtrate was concentrated by evaporation under reduced pressure followed by the addition of excess Et_2O. The resulting pale-yellow precipitate was recrystallized from acetonitrile/Et_2O, collected by filtration, washed with Et_2O, and dried in vacuo. Yield, 80.3 mg (0.214 mmol, 85%). Single crystals of **Pd-dFppy** were prepared by the slow vapor diffusion of ^{t}BuOMe into a solution of **Pd-dFppy** in non-dehydrated acetonitrile at 4 °C. ^{1}H NMR (400 MHz, CD_3CN): δ = 9.12 (dd, J = 5.5 and 0.9 Hz, 1H), 8.10 (d, J = 8.2 Hz, 1H), 7.95 (t, J = 8.3 Hz, 1H), 7.50 (dd, J = 8.2 and 2.3 Hz, 1H), 7.32 (t, J = 6.7 Hz, 1H), 6.64 (ddd, J = 13.0, 9.1, and 2.5 Hz, 1H); ^{13}C NMR (126 MHz, DMSO-d_6): δ = 166.7, 162.0 (d, $J_{C\text{-}F}$ = 8.6 Hz), 161.3 (dd, $J_{C\text{-}F}$ = 255.3 and 9.7 Hz), 158.9 (dd, $J_{C\text{-}F}$ = 261.3 and 11.0 Hz), 152.6, 142.1, 140.2, 130.1, 129.6, 123.4, 122.2 (d, $J_{C\text{-}F}$ = 20.5 Hz), 121.0 (dd, $J_{C\text{-}F}$ = 16.9 and 2.4 Hz), 100.2 (t, $J_{C\text{-}F}$ = 27.2 Hz); ESI-MS (MeOH, negative): m/z calcd. for $C_{13}H_6F_2N_3Pd^-$ ($[Pd(CN)_2(dFppy)]^-$): 347.96; found: 347.95; elemental analysis calcd. (%) for $C_{13}H_8F_2KN_3OPd$ ($K[Pd(CN)_2(dFppy)]\cdot H_2O$): C 38.48, H 1.99, N 10.36; found: C 38.44, H 1.97, N 10.21. The purity of the bulk polycrystalline sample of **Pd-dFppy** was also confirmed by PXRD and TG analyses (Figs. S1-S2).

Crystallization of $K[Pt(CN)_2(dFppy)]\cdot H_2O$ (Pt-dFppy). Single crystals of **Pt-dFppy** were prepared by the slow vapor diffusion of ^{t}BuOMe into a solution of **Pt-dFppy** in non-dehydrated acetonitrile at 4 °C. ^{1}H NMR (400 MHz, CD_3CN): δ = 9.41 (dd, J = 8.9 and 1.8 Hz, 1H), 8.13 (dd, J = 8.4 and 2.4 Hz, 1H), 8.01 (td, J = 8.0 and 1.8 Hz, 1H), 7.58 (dd, J = 8.7 and 2.3 Hz, 1H), 7.32 (td, J = 6.6 and 2.0 Hz, 1H), 6.64 (ddd, J = 12.9, 9.3, and 2.6 Hz, 1H); ^{13}C NMR (126 MHz, DMSO-d_6): δ = 163.1 (dd, $J_{C\text{-}F}$ = 254.0 and 10.8 Hz), 163.1 (d, $J_{C\text{-}F}$ = 7.2 Hz), 162.7, 159.9 (dd, $J_{C\text{-}F}$ = 260.1 and 12.1 Hz), 152.7, 141.7, 140.3, 129.6, 124.0, 122.4 (d, $J_{C\text{-}F}$ = 20.5 Hz), 119.5 (dd, $J_{C\text{-}F}$ = 16.9 and 2.4 Hz), 114.7, 99.6 (t, $J_{C\text{-}F}$ = 27.2 Hz); ESI-MS (MeOH, negative): m/z calcd. for $C_{13}H_6F_2N_3Pt^-$ ($[Pt(CN)_2(dFppy)]^-$): 437.01; found: 437.02; elemental analysis calcd. (%) for $C_{13}H_8F_2KN_3OPt$ ($K[Pt(CN)_2(dFppy)]\cdot H_2O$): C 31.58, H 1.63, N 8.50; found: C 31.50, H 1.69, N 8.55. The purity of the bulk polycrystalline sample of **Pt-dFppy** was also confirmed by PXRD and TG analyses (Figs. S1-S2).

Apparatus.

Single-crystal X-ray structural analysis. All single crystal X-ray diffraction measurements were performed using a Rigaku XtaLAB-Synergy diffractometer with a HyPix-6000HE area detector and a multilayer mirror-monochromated Cu K_α radiation (λ = 1.54184 Å). Each crystal was mounted on a MicroMount using Paratone-N oil, and cooled using a N_2-flow type temperature controller. Diffraction data were collected and processed using *CrysAlisPro*.[S2] The structures were solved using *SHELXT-2018*[S3] by the intrinsic phasing method. Structure refinements were conducted by the full-matrix least squares techniques with *SHELXL-2018*.[S4] All non-hydrogen atoms were refined anisotropically, and hydrogen atoms were refined using the riding model. The crystallographic data and the selected interatomic distances and angles of complexes are listed in Tables S5-S8. Full crystallographic data have been deposited with the Cambridge Crystallographic Data Centre (CCDC 2357833-2357844).

Luminescence measurements. Emission spectra were acquired using a JASCO FP-8600 spectrometer. The typical slit widths of the excitation and emission lights were 5 nm. The MeOH/EtOH (v/v = 1/1) mixture was used for a transparent glass matrix at 77 K. Emission quantum yields were recorded with a Hamamatsu Photonics C9920-02 absolute photoluminescence quantum yield measurement system equipped with an integrating sphere apparatus and 150 W CW xenon light source. The accuracy of the instrument was confirmed based on a measurement of the quantum yield of anthracene in ethanol (Φ = 0.27).[S5] Emission lifetime measurements at 293 K were conducted using a Hamamatsu Photonics Quantaurus-Tau C11367 fluorescence lifetime spectrometer excited by an internal light-emitting diode (LED) (λ_{ex} = 470 nm). For variable-temperature measurements of emission lifetimes and emission spectra, a streak camera (Hamamatsu Photonics, C10910-05) system combined with a spectrometer (Hamamatsu Photonics, C11119-04) and a thermoelectrically cooled charge-coupled device (Princeton Instruments, PIXIS-256E) were used equipped with a closed-cycle refrigerator cryostat (Nagase Techno-Engineering, PS25-SS and Cryogenic Control Systems, Model 22C cryogenic temperature

controller). To prevent the dehydration of the sample in the cryostat, each sample was sealed in a glass capillary (0.5 mmφ, Hilgenberg GmbH.) and subsequently fixed to a copper-made sample holder prior to introduction into the cryostat. Widely wavelength-tunable pulsed laser (Spectra-Physics InSight X3) with a repetition rate of 80 MHz and a pulse width of < 120 fs was used as the excitation source. Before laser irradiation of the samples, the excitation wavelength and the repetition rate were tuned to 400 or 500 nm and 10 kHz using a pulse selector with harmonic generation (Spectra-Physics, UHG), respectively. The laser power was adjusted to 200 nW and further weakened with an ND filter, as necessary. The accuracy of the emission spectral shapes at 77 K was further confirmed by a comparison with those measured with a multichannel photodetector (Hamamatsu Photonics, PMA-11 C7473) and a nitrogen laser (Usho, KEN-X) for 337 nm excitation, where a liquid nitrogen cryostat (Oxford instruments, Optistat-DN optical Dewer and ITC-503 temperature controller) was used for temperature control. The emission decays were analyzed using two exponentials: $I = A_1\exp(-t/\tau_1) + A_2\exp(-t/\tau_2)$, where A_i (i = 1, 2) denotes the pre-exponential factors for lifetimes τ_i. The average emission lifetimes (τ_{av}) were calculated using following equation: $\tau_{av} = (A_1\tau_1^2 + A_2\tau_2^2) / (A_1\tau_1 + A_2\tau_2)$.[S6]

Other measurements. The UV-vis absorption spectra were recorded on a Shimadzu UV-2500PC spectrophotometer. The UV-vis diffuse-reflectance spectra were recorded on the same spectrometer equipped with an integrating sphere apparatus. The reflectivity of the solid samples was converted using the Kubelka-Munk function. The ^{1}H and ^{13}C NMR spectra were measured using a JEOL JNM-ECZ400S and a JEOL JNM-ECZ500R NMR spectrometers, respectively, where chemical shifts were referenced to the internal tetramethylsilane. Powder X-ray diffraction (PXRD) measurements were conducted using Cu K_α radiation (λ = 1.5418 Å) on a Bruker D8 Advance diffractometer equipped with a graphite monochromator and a one-dimensional LynxEye detector using a nonreflective plate. Thermogravimetric (TG) analyses were conducted by Rigaku Thermoplus EVO TG 8120 under Ar flow (0.3 L min^{-1}). Electrospray-ionization mass spectrometry (ESI-MS) were performed on the JEOL JMS-T100LP spectrometer at the Analysis Center at Hokkaido University. Elemental analyses were carried out on the Exeter Analytical CE440 elemental analyzer at the Analysis Center at Hokkaido University and the J-SCIENCE LAB JM-11 elemental analyzer at the Organic Microanalysis Laboratory at Kyoto University.

Computational methods.

Ground state structure optimizations were performed using the density functional theory (DFT), as implemented in the Gaussian16 program.[S7] Starting from the ground state optimized structures, vertical excitations were computed using the time-dependent density functional theory (TD-DFT). For the simple models, $[M(CN)_2(dFppy)][K(H_2O)]$ (M =Pd or Pt), DFT or TDDFT were used for the full system. The positions of K, O, and N atoms of the CN^- groups were frozen during the structure optimizations. The PBE1PBE functional,[S8] SDD[S9] basis set and the associated effective core potential

for Pt or Pd, and def2-SVP[S10] basis sets were applied. For the solid-state model systems, the two-layer ONIOM method[S11] was used. The PBE1PBE functional[S8] and the basis sets mentioned above were applied for the ONIOM-high layer (Fig. S10). The universal force field (UFF)[S12] was used for the ONIOM low-layer (Fig. S10). Vibrational frequency calculations were performed to confirm the nature of the optimized ground-state local minima (i.e., no imaginary frequencies).

An energy decomposition analysis (EDA)[S13] was performed together with the natural orbitals for chemical valence (NOCV)[S14] for the Pt2 and Pd2 systems (Fig. S10). Only the atoms in the ONIOM high-layer were taken for the EDA-NOCV. The interaction energy (ΔE_{int}) between the Pt (or Pd) units were separated into Pauli repulsion ($\Delta E_{\mathrm{Pauli}}$), electrostatic attraction ($\Delta E_{\mathrm{elstat}}$), orbital interactions (ΔE_{orb}), and dispersion energy (ΔE_{disp}). The EDA-NOCV was performed using the BP86[S15] functional, including empirical dispersion corrections,[S16] as it was used for transition metal complexes.[S17] The TZP[S10] basis sets were applied for all atoms. The relativistic Scalar ZORA[S18] approach was applied for Pt and Pd.

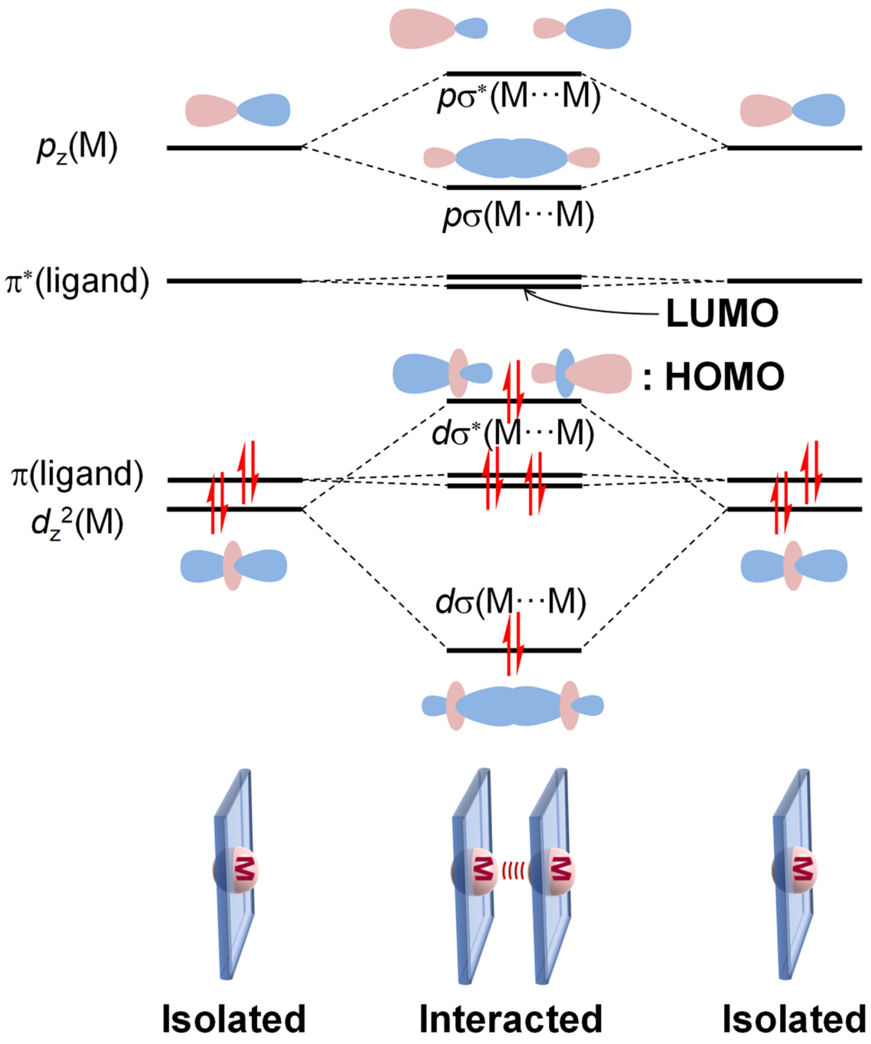

Scheme S1 Schematic MO energy level diagram for square-planar d^8 metal complexes (M = Pt, Pd) with appropriate aromatic ligands showing effective M···M interaction by stacking.

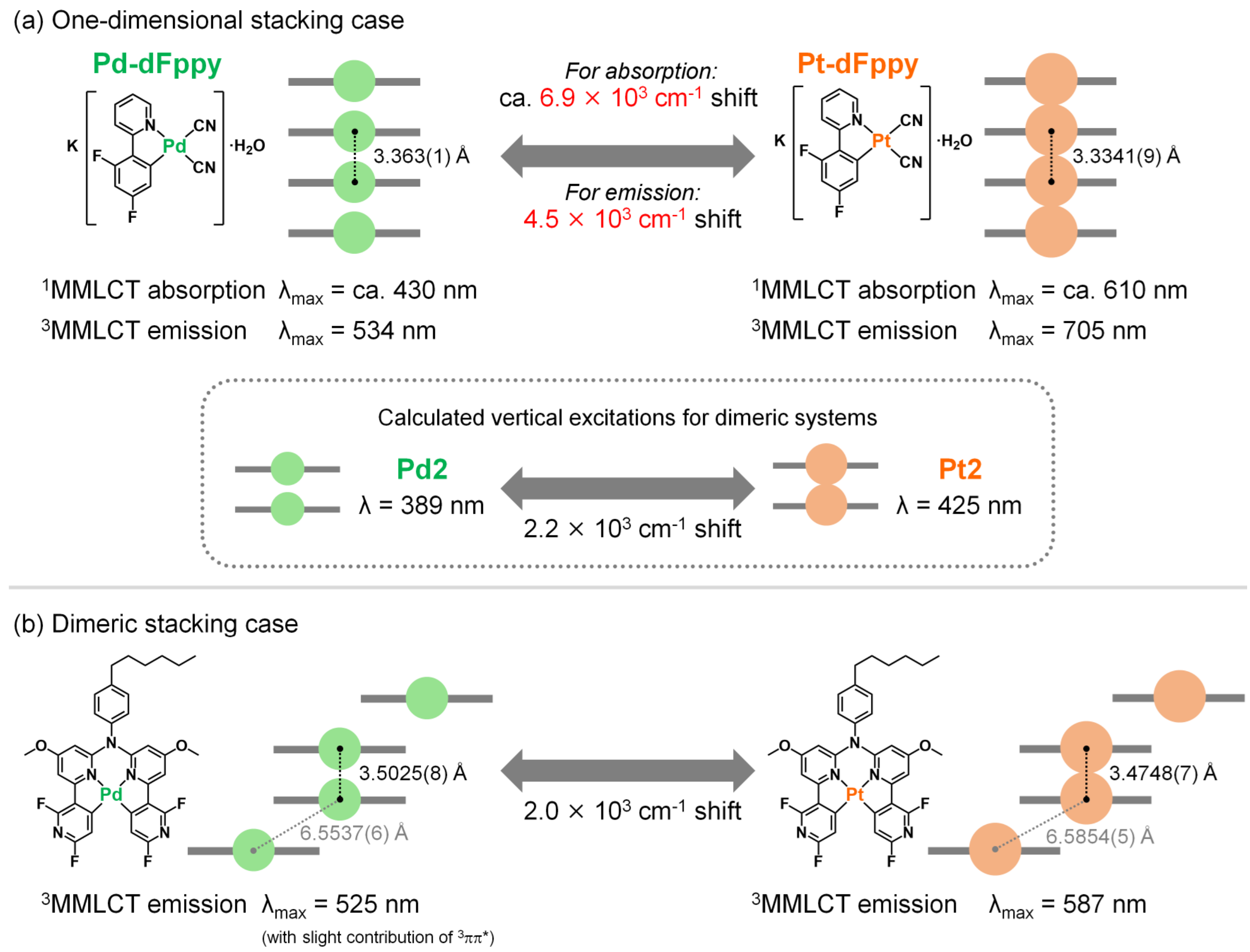

Scheme S2 Stacking modes and emission maxima of isostructural Pd(II) and Pt(II) complexes in (a) this study and (b) the Strassert's previous report.[S19] In the Strassert's case, the shift in the dimeric ^{3}MMLCT emission of Pt(II) complex relative to Pd(II) complex was about 2.0×10^3 cm^{-1}. This shift is almost the same as the shift of the vertical transition energies for the dimeric unit of **Pd-dFppy** (Pd2) and **Pt-dFppy** (Pt2) obtained by TDDFT (2.2×10^3 cm^{-1}; see "Computational Studies"). However, the ^{3}MMLCT emission of **Pt-dFppy** was more largely red-shifted by 4.5×10^3 cm^{-1} compared to **Pd-dFppy**, suggesting that **Pt-dFppy** formed more delocalized excited oligomers than **Pd-dFppy**.

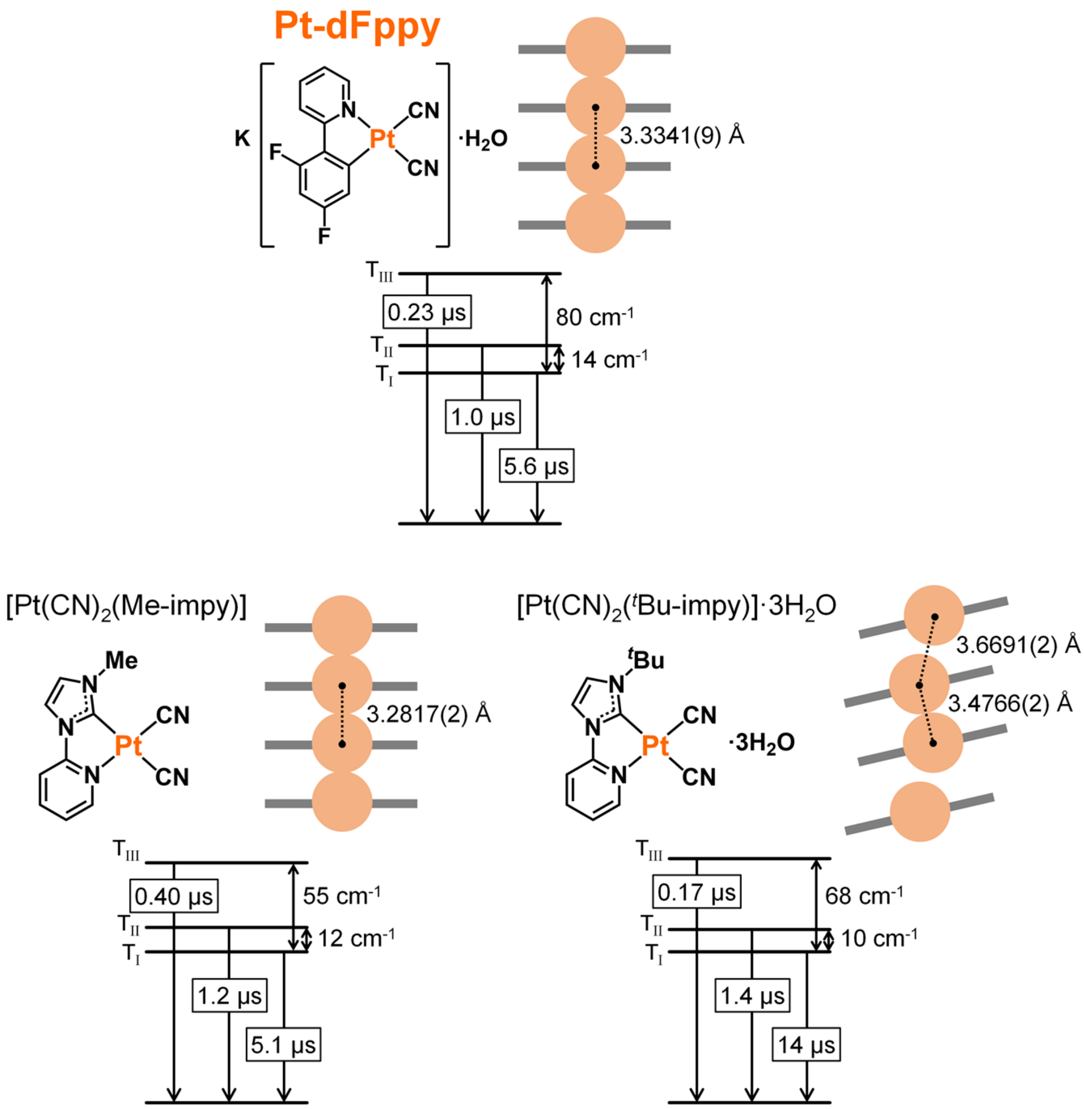

Scheme S3 Structures and schematic energy diagrams of the spin sublevels of ^{3}MMLCT states of present and previously reported[S20] self-assembled Pt(II) complexes.

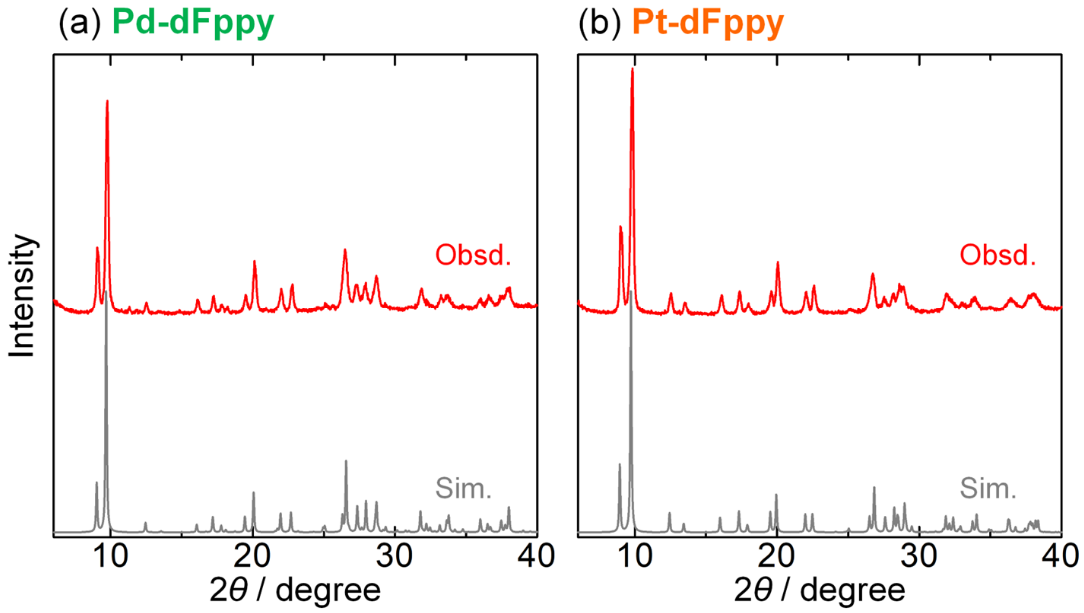

Fig. S1 PXRD patterns of (a) **Pd-dFppy** and (b) **Pt-dFppy**. Gray lines indicate the simulated patterns based on the crystal structures of **Pd-dFppy** and **Pt-dFppy**.

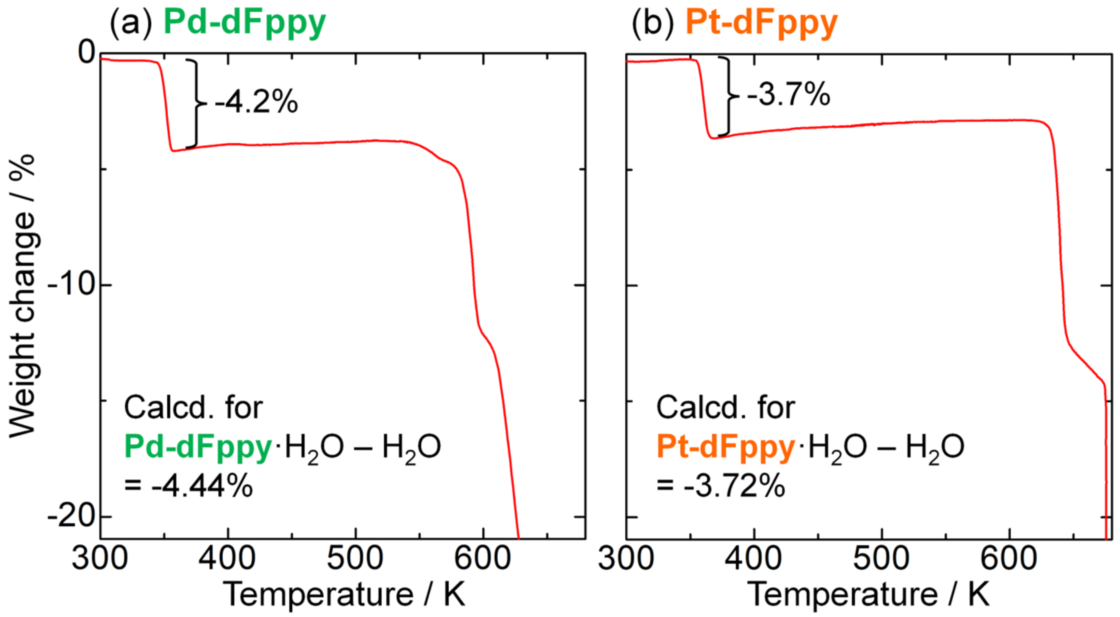

Fig. S2 TG curves of (a) **Pd-dFppy** and (b) **Pt-dFppy** (scan rate = 1 K min^{-1}). The observed weight losses were well consistent with the calculated values, indicating the phase purities of the polycrystalline samples.

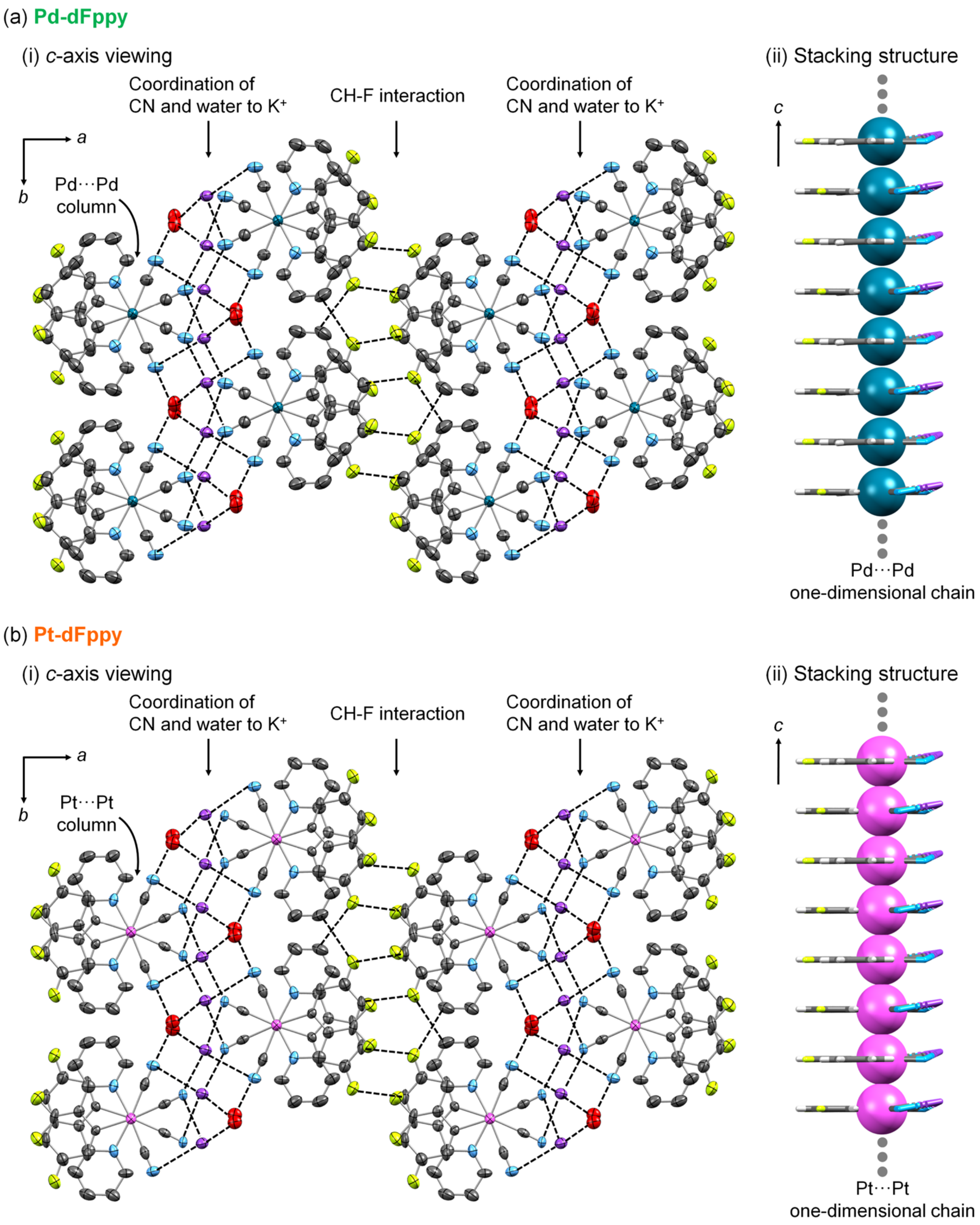

Fig. S3 (i) Packing and (ii) stacking structures of (a) **Pd-dFppy** and (b) **Pt-dFppy** at 240 K. For the packing diagram, the H atoms are omitted for clarity and thermal ellipsoids are displayed at the 50% probability level. For the stacking diagram, the Pd and Pt atoms are shown with a space-filling model, while the other parts are shown in a capped stick model. Color codes for atoms: pink: Pt, green: Pd, gray: C, cyan: N, red: O, yellow-green: F, purple: K. For **Pd-dFppy**, only one of the two disordered parts of dFppy ligands, K^+ ions, and water molecules are shown for clarity.

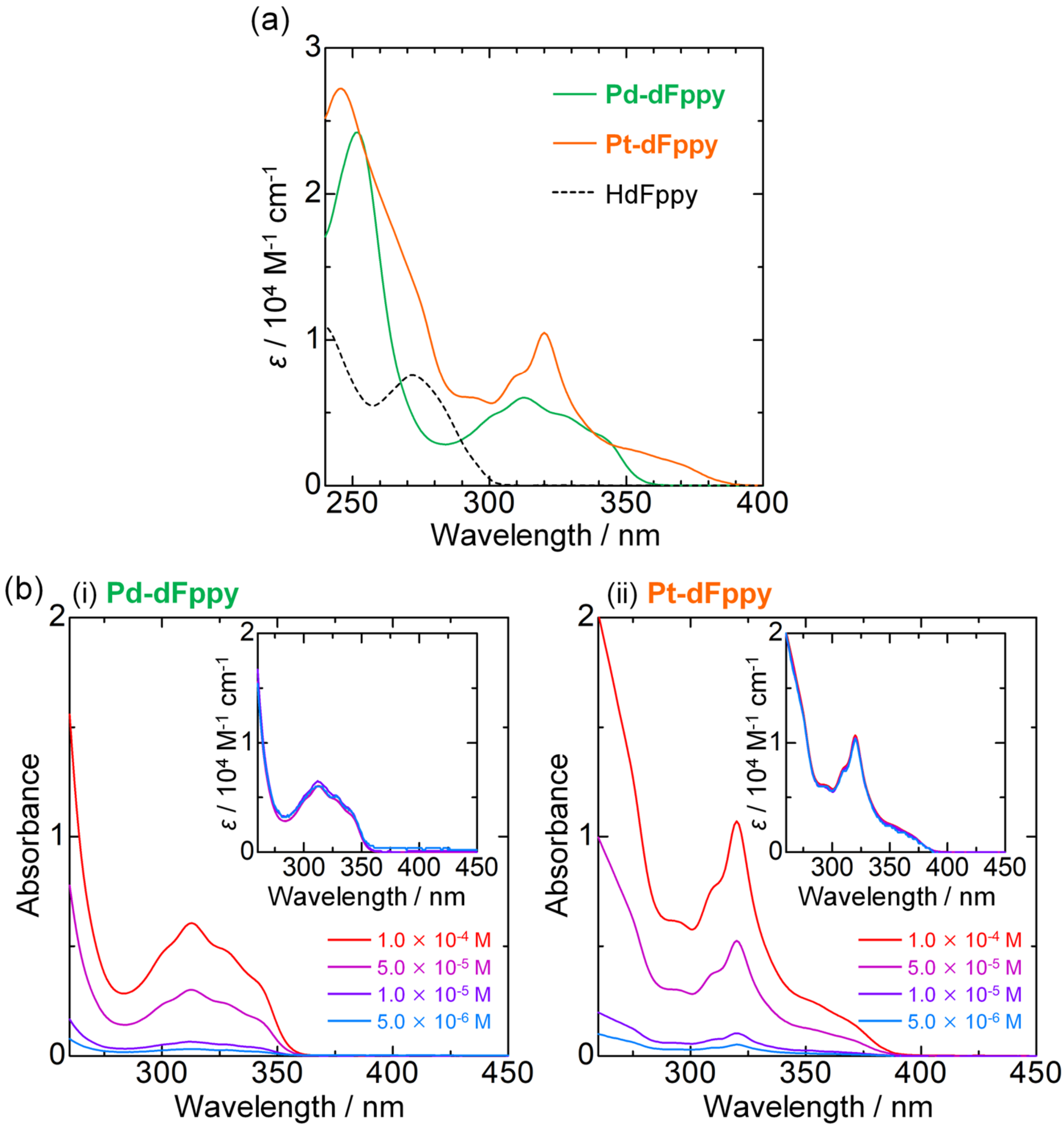

Fig. S4 (a) UV-vis absorption spectra of **Pd-dFppy** (green line), **Pt-dFppy** (orange line), and HdFppy (black dashed line) in MeOH at 293 K (5.0×10^{-5} M). (b) The concentration dependence of UV-vis absorption spectra of (i) **Pd-dFppy** and (ii) **Pt-dFppy** in MeOH at 293 K (optical path length = 1 cm). These results demonstrate that the self-assembly of the complexes in solution is negligible in this concentration range, probably due to the electrostatic repulsion between anionic complexes.

(a)

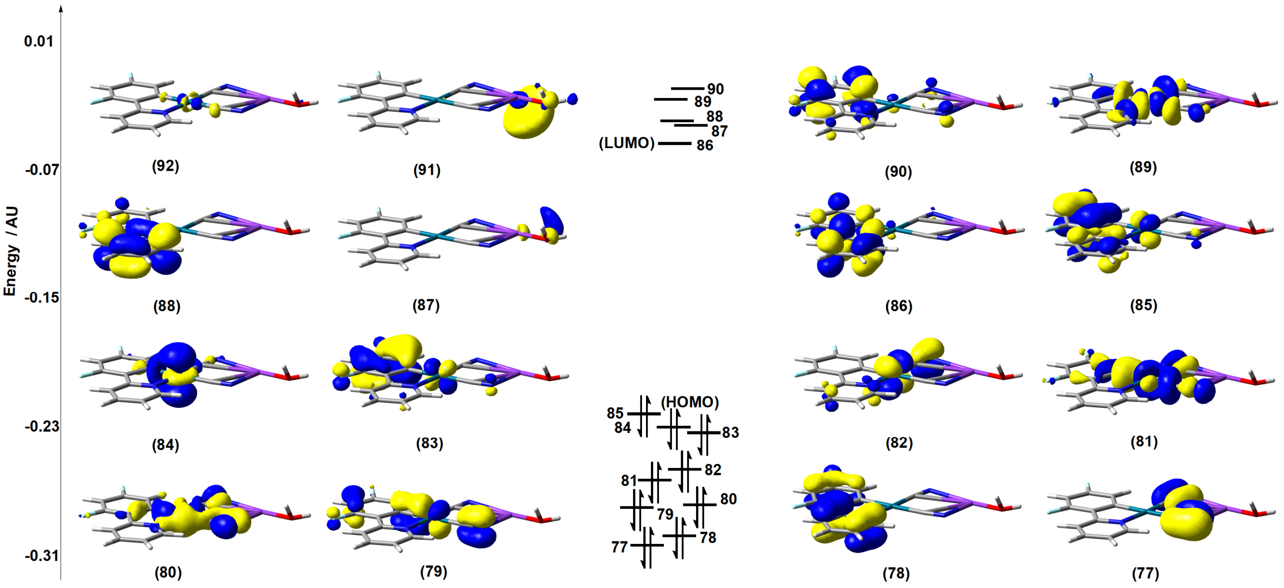

(b)

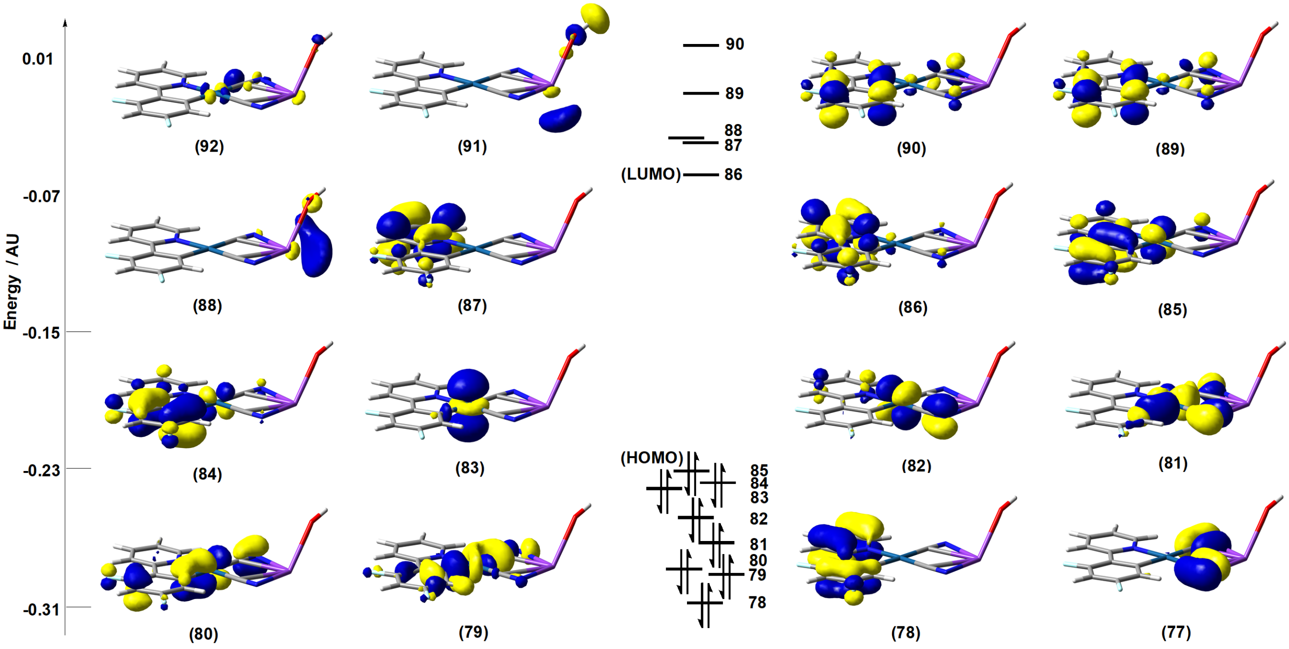

Fig. S5 Kohn-Sham frontier orbitals of the (a) [K(H_2O)][Pd($CN)_2$(dFppy)] and (b) [K(H_2O)][Pd($CN)_2$(dFppy)] systems (isovalue = 0.035).

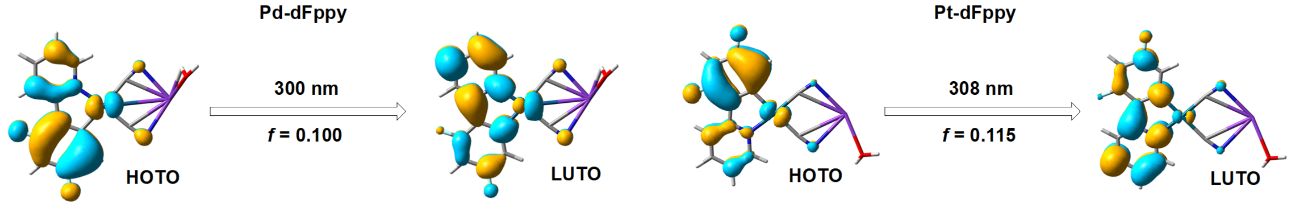

Fig. S6 Natural transition orbitals (NTOs) of the optimized S_0 states of monomers for the key vertical excitations. The highest occupied transition orbital (HOTO) and the lowest unoccupied transition orbital (LUTO) indicate the occupied "hole" and the unoccupied "electron", respectively. These results suggest that the lowest singlet excited states of **Pd-dFppy** and **Pt-dFppy** in the monomer states (i.e., [K(H_2O)][Pd($CN)_2$(dFppy)] and [K(H_2O)][Pt($CN)_2$(dFppy)] model systems, respectively) could be assignable to the ^{1}MLCT transition mixed with the ligand-centered $^1\pi\pi^*$ transition (300 nm for the Pd system and 308 nm or Pt system are in agreement with the experimental data).

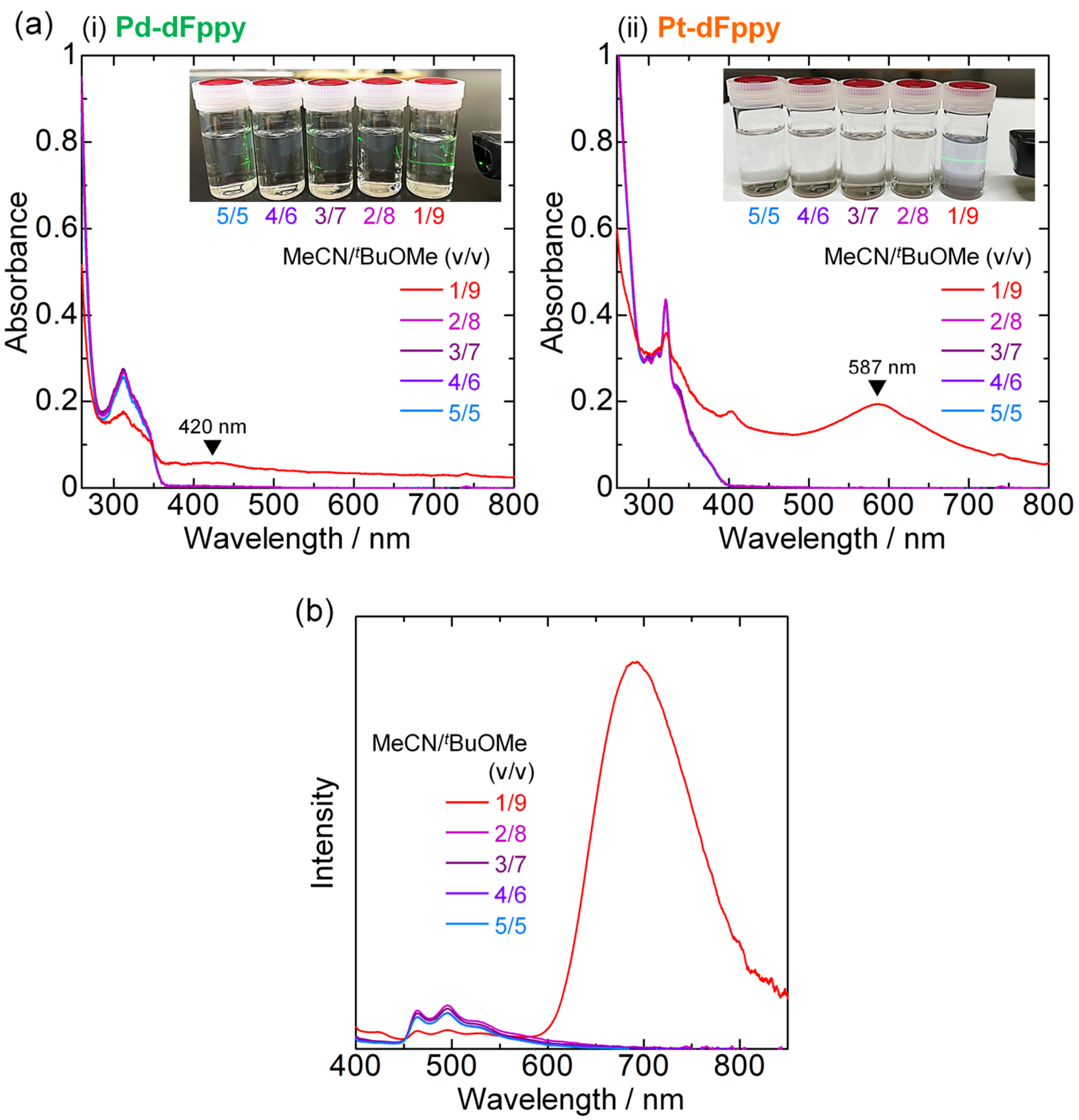

Fig. S7 (a) UV-vis absorption spectra of solutions (5.0×10^{-5} M) of (i) **Pd-dFppy** and (ii) **Pt-dFppy** in the MeCN/^{t}BuOMe mixture with different volume ratios at 293 K (insets: photographs of solutions; the green laser from the right shows the Tyndall effect). (b) Emission spectra of the **Pt-dFppy** solutions (5.0×10^{-5} M; air-saturated; λ_{ex} = 350 nm) in the MeCN/^{t}BuOMe mixture with different volume ratios at 293 K.

In both solutions, the absorption spectra remained almost unchanged up to a solvent ratio of 2/8. However, new absorption bands appeared in the visible region at a ratio of 1/9. The observed increase in the baseline and the Tyndall effect indicate that the complexes formed aggregates in the MeCN/^{t}BuOMe (v/v = 1/9) mixture. These new absorption bands are consistent with those observed in the solid state and are attributed to the ^{1}MMLCT absorption bands. Although the emission of the **Pd-dFppy** aggregate was very weak and undetectable by the spectrometer, the **Pt-dFppy** aggregate exhibited ^{3}MMLCT emission (λ_{max} = 692 nm) similar to that in the solid state.

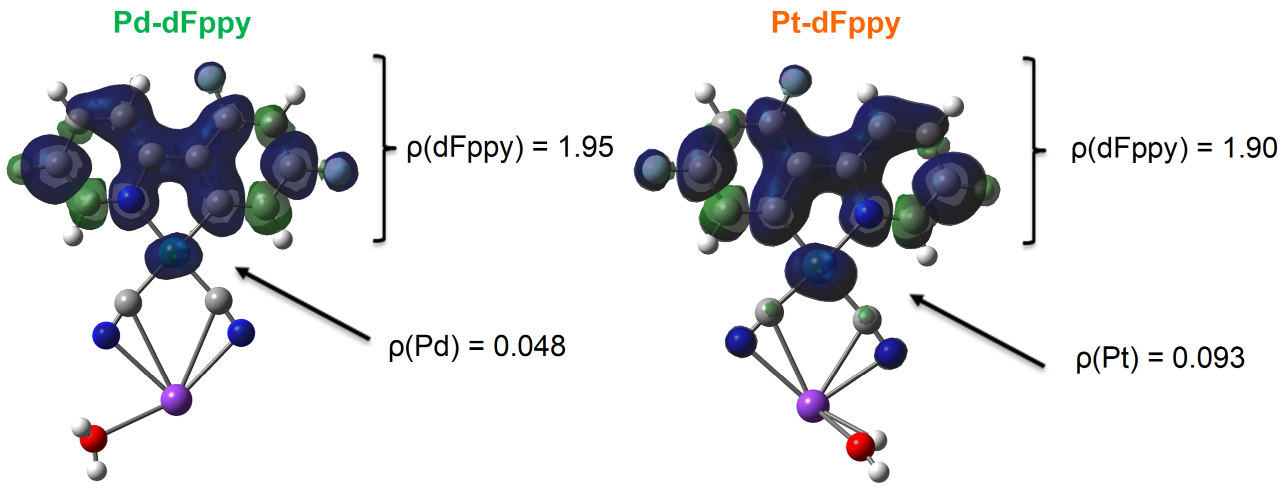

Fig. S8 The spin density plots (isovalue = 0.002) for the optimized T_1 states of monomers, obtained by DFT single-point calculations of T_1 state optimized structures. The spin densities are localized mainly on the dFppy ligand, indicating that the emission origins of discrete **Pd-dFppy** and **Pt-dFppy** can be assigned as the ligand-centered $^3\pi\pi^*$ transition, as previously assigned for $(Bu_4N)[Pt(CN)_2(dFppy)]$.[S1]

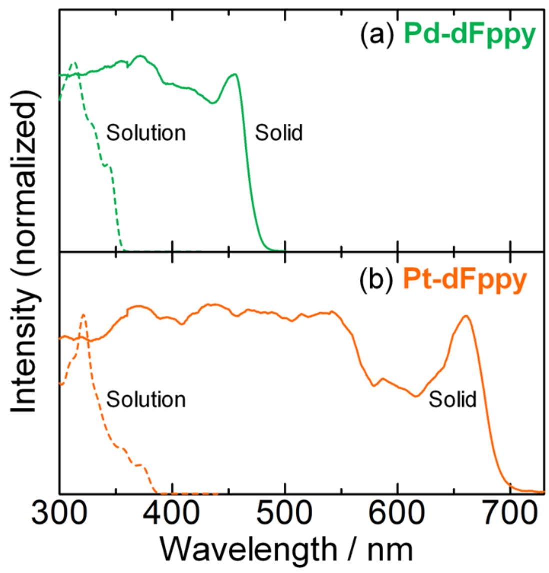

Fig. S9 Excitation spectra of (a) **Pd-dFppy** and (b) **Pt-dFppy** at 77 K in the solid state (solid lines) or in the MeOH/EtOH (v/v = 1/1) solution (5.0×10^{-5} M; dashed lines). Detection wavelengths were 520 nm (**Pd-dFppy**, solid), 448 nm (**Pd-dFppy**, solution), 800 nm (**Pt-dFppy**. solid), and 458 nm (**Pt-dFppy**, solution). Shapes of the excitation spectra are well consistent with those of the corresponding absorption spectra.

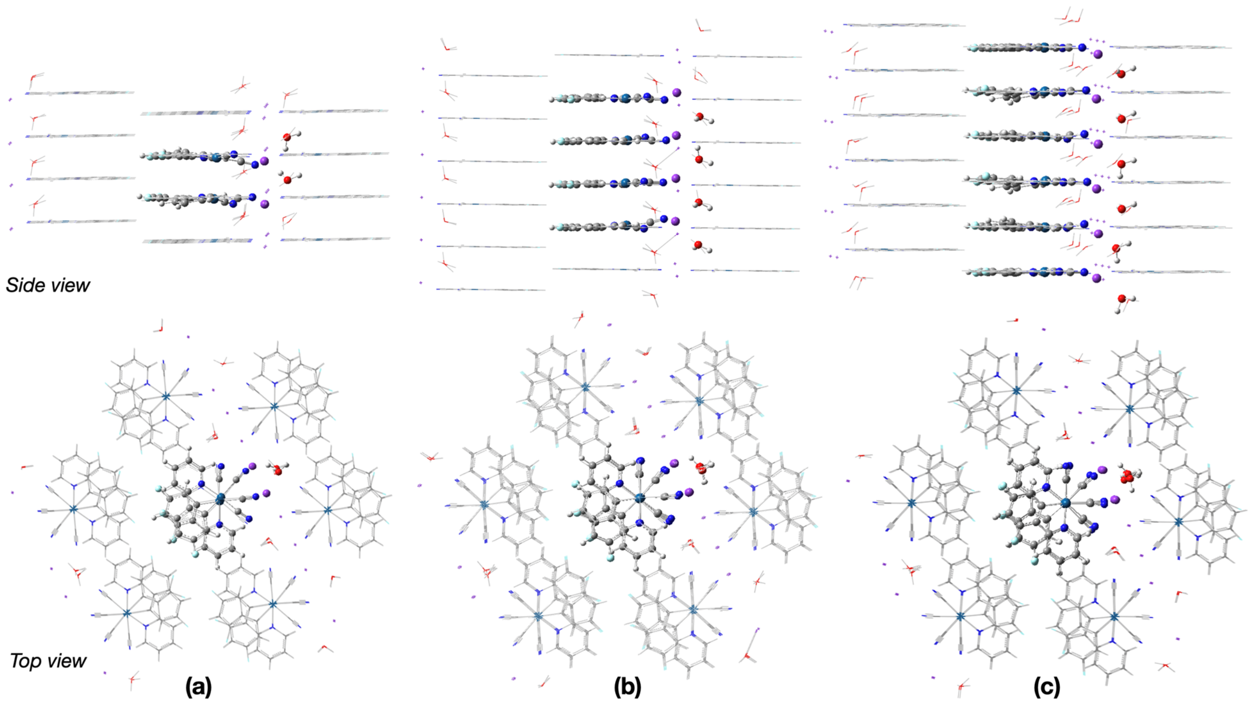

**M2**	**M4**	**M6**
**ONIOM high-layer**	**ONIOM high-layer**	**ONIOM high-layer**
(M-dFppy.H_2O.K^+)$_2$	(M-dFppy.H_2O.K^+)$_4$	(M-dFppy.H_2O.K^+)$_8$
**ONIOM low-layer**	**ONIOM low-layer**	**ONIOM low-layer**
(M-dFppy.H_2O.K^+)$_{20}$	(M-dFppy.H_2O.K^+)$_{38}$	(M-dFppy.H_2O.K^+)$_{36}$

Fig. S10 Top and side views of the cluster models employed in this study. The ONIOM high-layer is shown in *ball-and-stick* model, and ONIOM low-layer is shown in *wireframe* model. (a) M2, M4, and M6 systems, where M = Pd or Pt. In M6 systems, the outermost (M-dFppy.H_2O.K^+) units of the ONIOM-high layer were frozen during the structure optimizations.

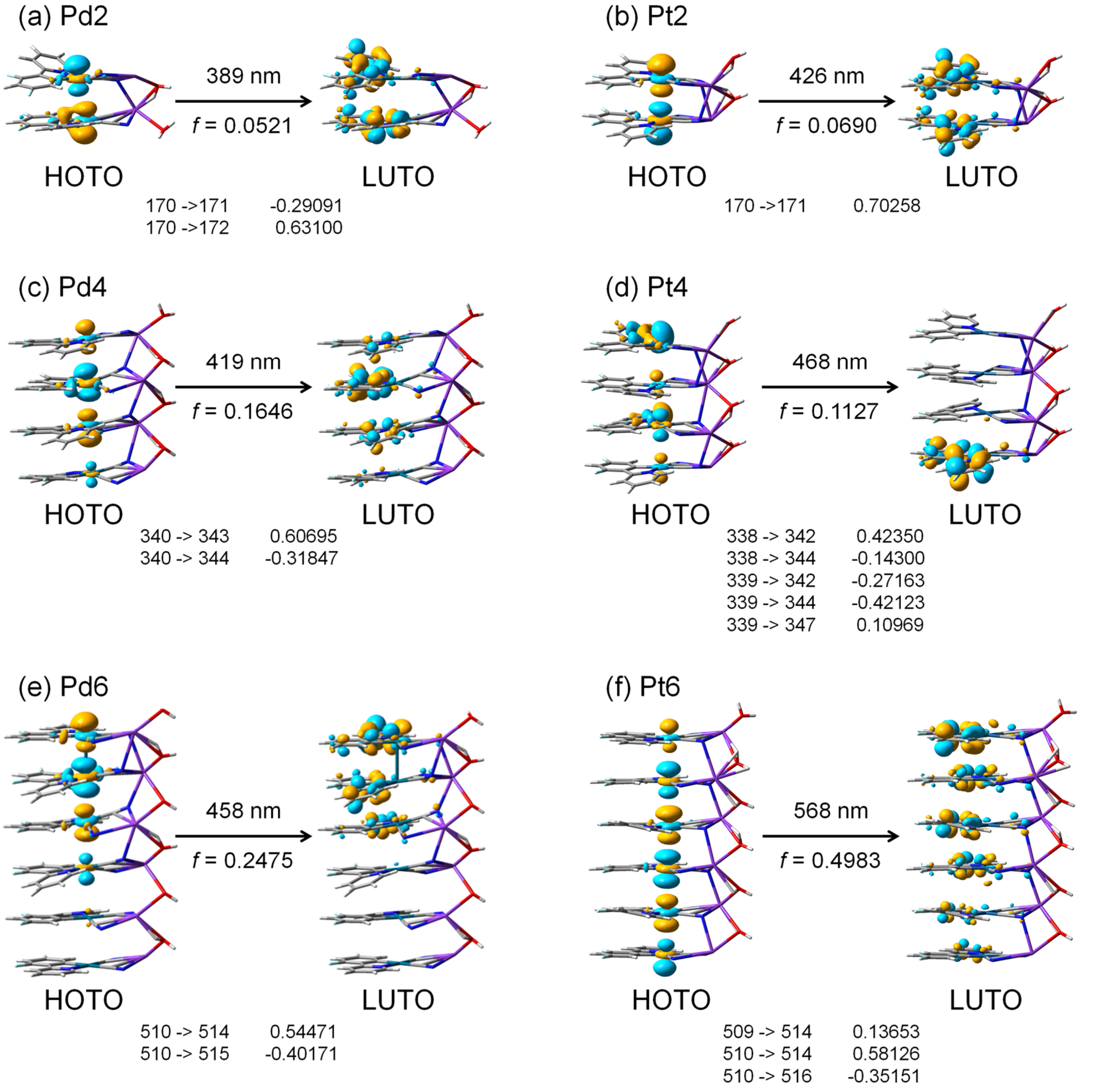

Fig. S11 Natural transition orbitals (NTOs) for the vertical ^{1}MMLCT excitations of (a) Pd2, (b) Pt2, (c) Pd4, (d) Pt4, (e) Pd6, and (f) Pt6 systems. The highest occupied transition orbital (HOTO) and the lowest unoccupied transition orbital (LUTO) indicate the occupied "hole" and the unoccupied "electron", respectively.

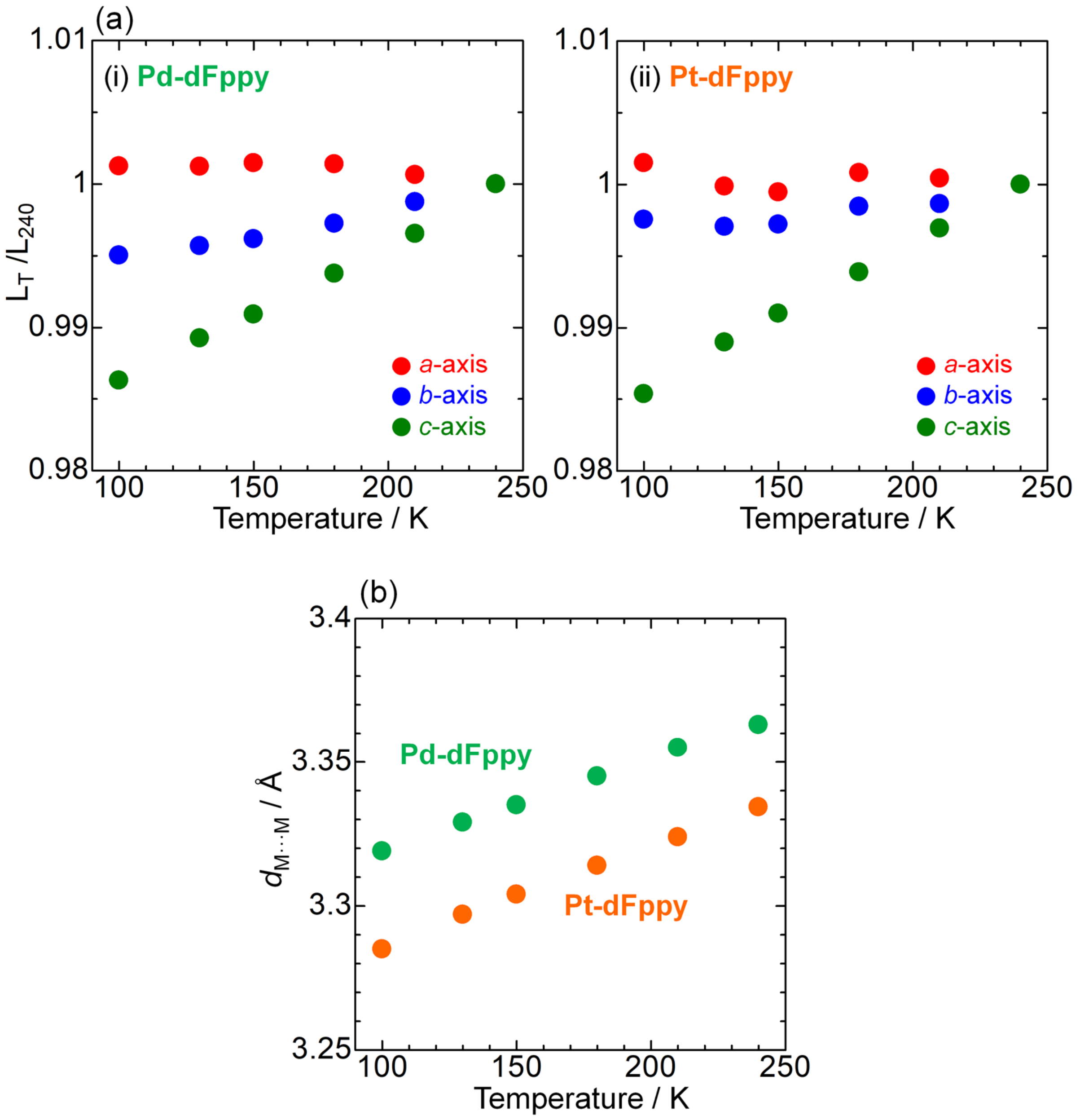

Fig. S12 (a) Ratio of the cell lengths (*a*, *b*, and *c*) of (i) **Pd-dFppy** and (ii) **Pt-dFppy** relative to those at 240 K. Among the three cell axes, *c*-axis showed the significant thermal expansion behavior. (b) Temperature dependence of the M···M distances ($d_{M\cdots M}$) of **Pd-dFppy** (green) and **Pt-dFppy** (orange).

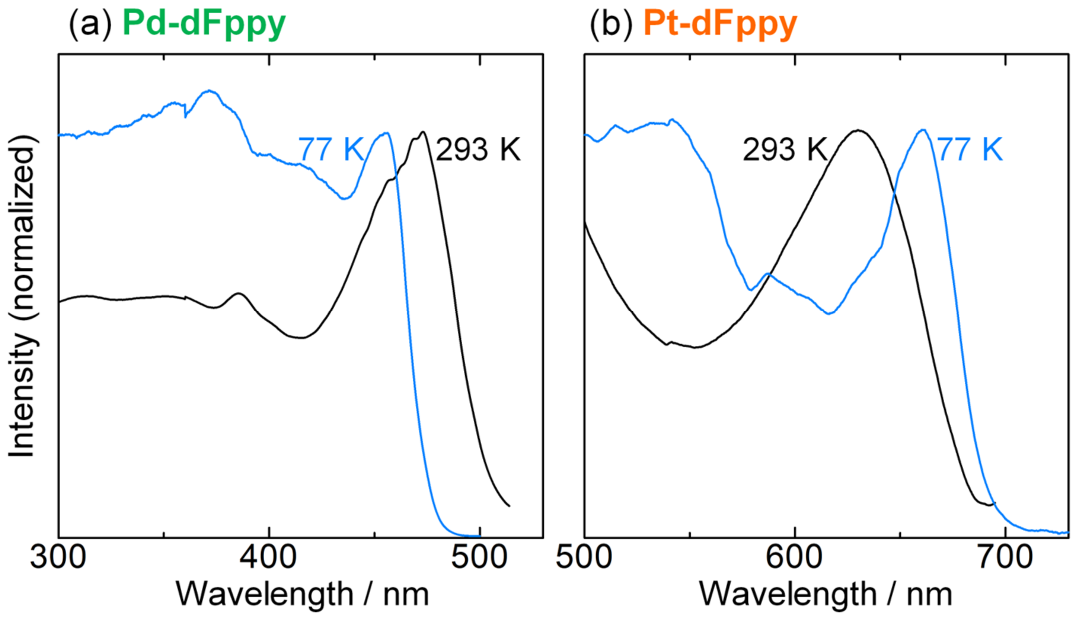

Fig. S13 Excitation spectra of (a) **Pd-dFppy** and (b) **Pt-dFppy** at 293 K (black) and 77 K (blue) in the solid state. The spectra were recorded at each emission maximum wavelength. The excitation edge of **Pd-dFppy** exhibited a slight blue-shift at 77 K due to the suppression of molecular vibrations, as is typically observed in such cases. On the other hand, the excitation edge of **Pt-dFppy** exhibited a red-shift at 77 K, indicating a temperature-dependent change in the Franck-Condon ^{1}MMLCT excited state.

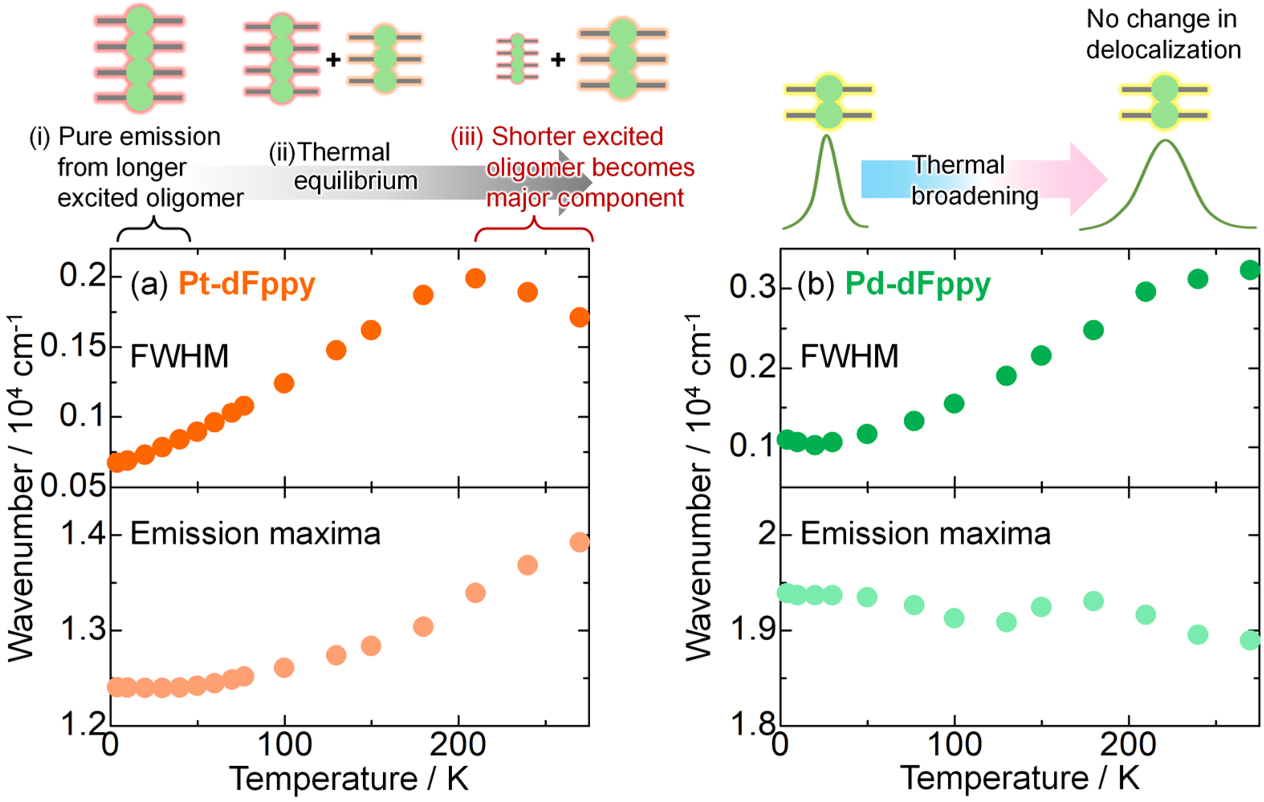

Fig. S14 Plots showing the temperature dependence of full width at half maximum (FWHM; top) and emission maximum energy (bottom) of the emission band of (a) **Pt-dFppy** (λ_{ex} = 500 nm) and (b) **Pd-dFppy** (λ_{ex} = 400 nm). The FWHM of **Pd-dFppy** increased continuously with increasing temperature, whereas that of **Pt-dFppy** turned to decrease above around 210 K.

The temperature dependence of FWHM of **Pt-dFppy** could be explained as follows: At very low temperatures (region (i)), ^{3}MMLCT emission was derived mainly from the longer excited oligomer (e.g., excited tetramer), as indicated by the almost constant emission maximum energy. At higher temperatures (region (ii)), the thermal equilibrium between the excited oligomers resulted in a significant high-energy shift and an increase in the FWHM. On the other hand, above 210 K (region (iii)), the FWHM decreased with increasing temperature, suggesting that the shorter excited oligomer (i.e., excited trimer) would become the major component of the emission band.

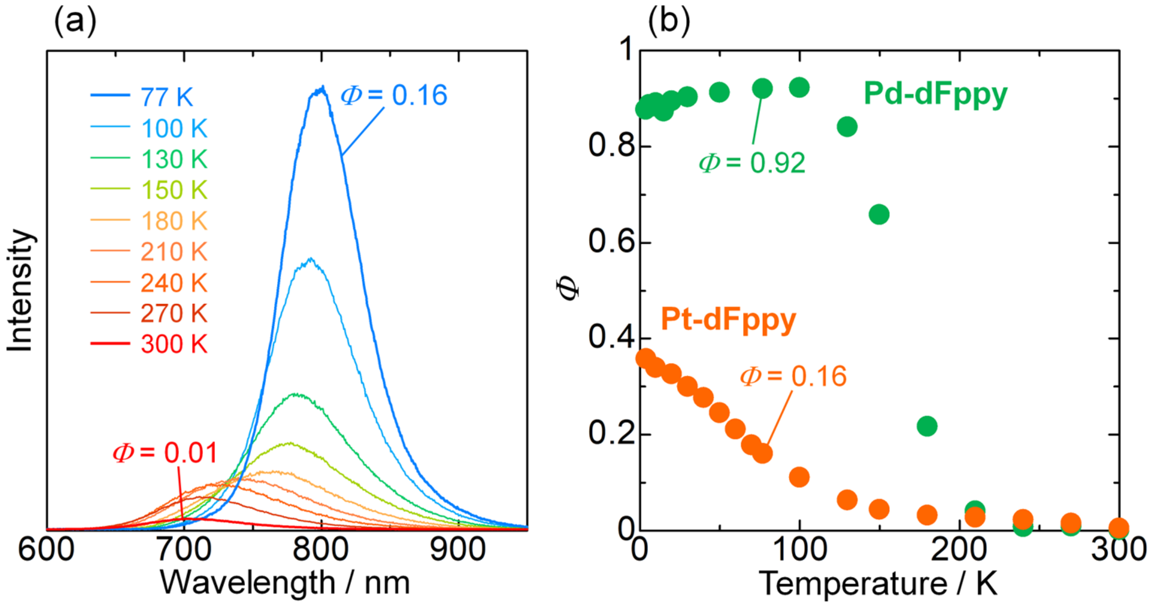

Fig. S15 Temperature dependence of the (a) non-normalized emission spectra of **Pt-dFppy** (λ_{ex} = 500 nm), and (b) emission quantum yield of **Pd-dFppy** (green; λ_{ex} = 400 nm) and **Pt-dFppy** (orange; λ_{ex} = 500 nm) in the solid state. The emission quantum yield values at temperatures other than 77 K were determined by measuring the relative peak areas of the emission bands and were set in relation to the value for absolute emission quantum yield at 77 K. Compared to the **Pd-dFppy** data, the temperature dependence of the emission quantum yield of **Pt-dFppy** is less drastic and shows a gradual change.

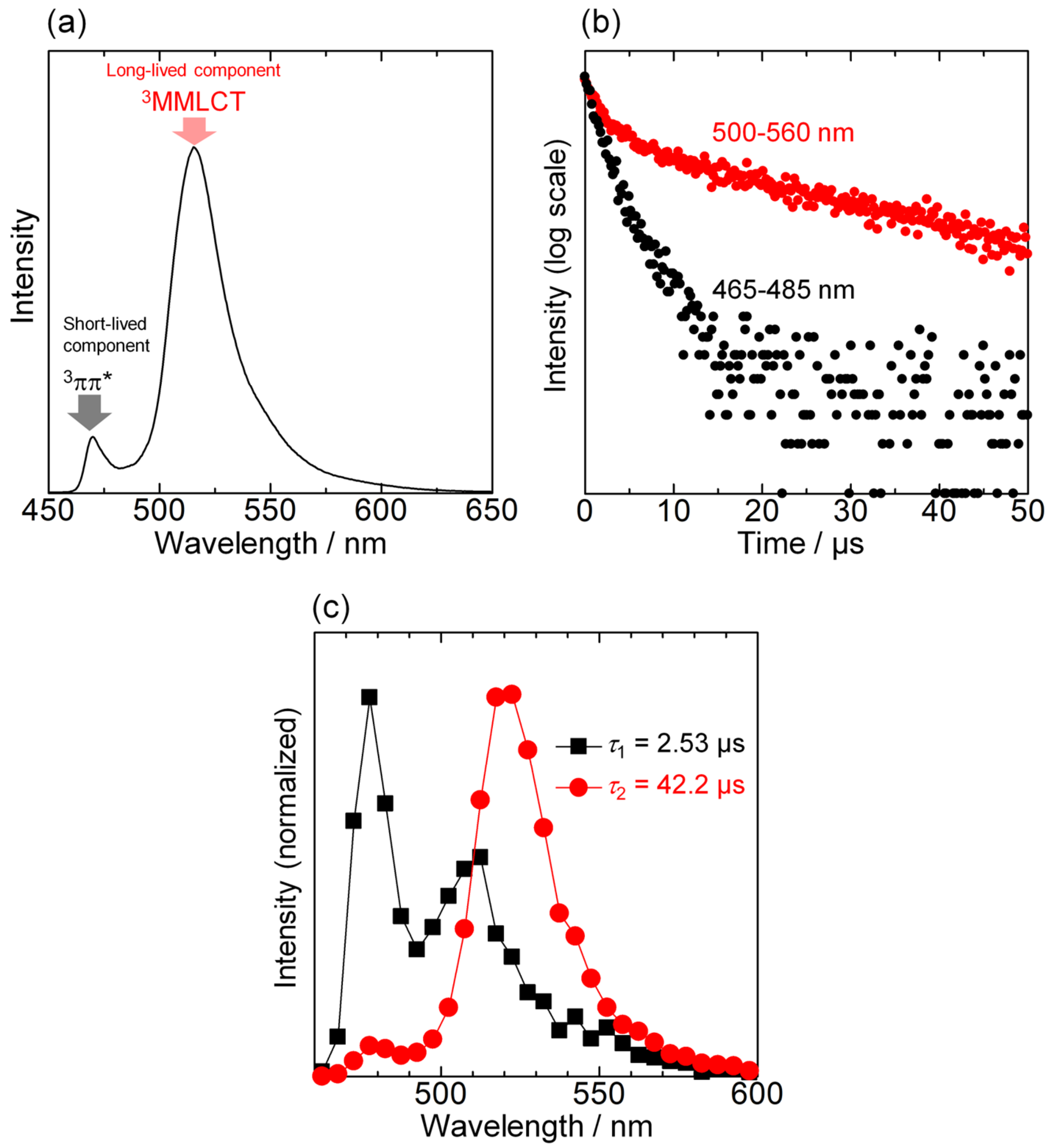

Fig. S16 (a) Steady-state emission spectrum, (b) emission decay curves detected at 465-485 nm (black) and 500-560 nm (red), and (c) spectra of short-lived (black) and long-lived (red) components obtained by the global fitting of emission decays (at 460-600 nm) of **Pd-dFppy** at 3.8 K (λ_{ex} = 400 nm).

A steady-state emission spectrum at 3.8 K showed the appearance of new emission band at 470 nm. The emission decay curve detected at 465-485 nm contained a minimal ^{3}MMLCT-derived long-lived component compared to that detected at 500-560 nm, suggesting the presence of a short-lived component derived from a distinct emission origin than ^{3}MMLCT. In fact, the emission spectrum of the short-lived component obtained by the global fitting clearly showed the vibrational progression, revealing that this short-lived emission band can be assignable to ligand-centered $^3\pi\pi^*$ emission. These results indicate a competition between $^3\pi\pi^*$ and ^{3}MMLCT emissions at very low temperatures, as similarly observed for [Pt(CN)$_2$(*i*-biq)] (*i*-biq = 3,3'-biisoquinoline).[S21] Therefore, for the analysis of ZFS (shown in Fig. 7(a)), only the long-lived component assignable to ^{3}MMLCT emission was used for the fitting below 20 K, since the $^3\pi\pi^*$ emission band clearly appeared in this temperature range.

Supplementary figures for characterization and DFT calculation

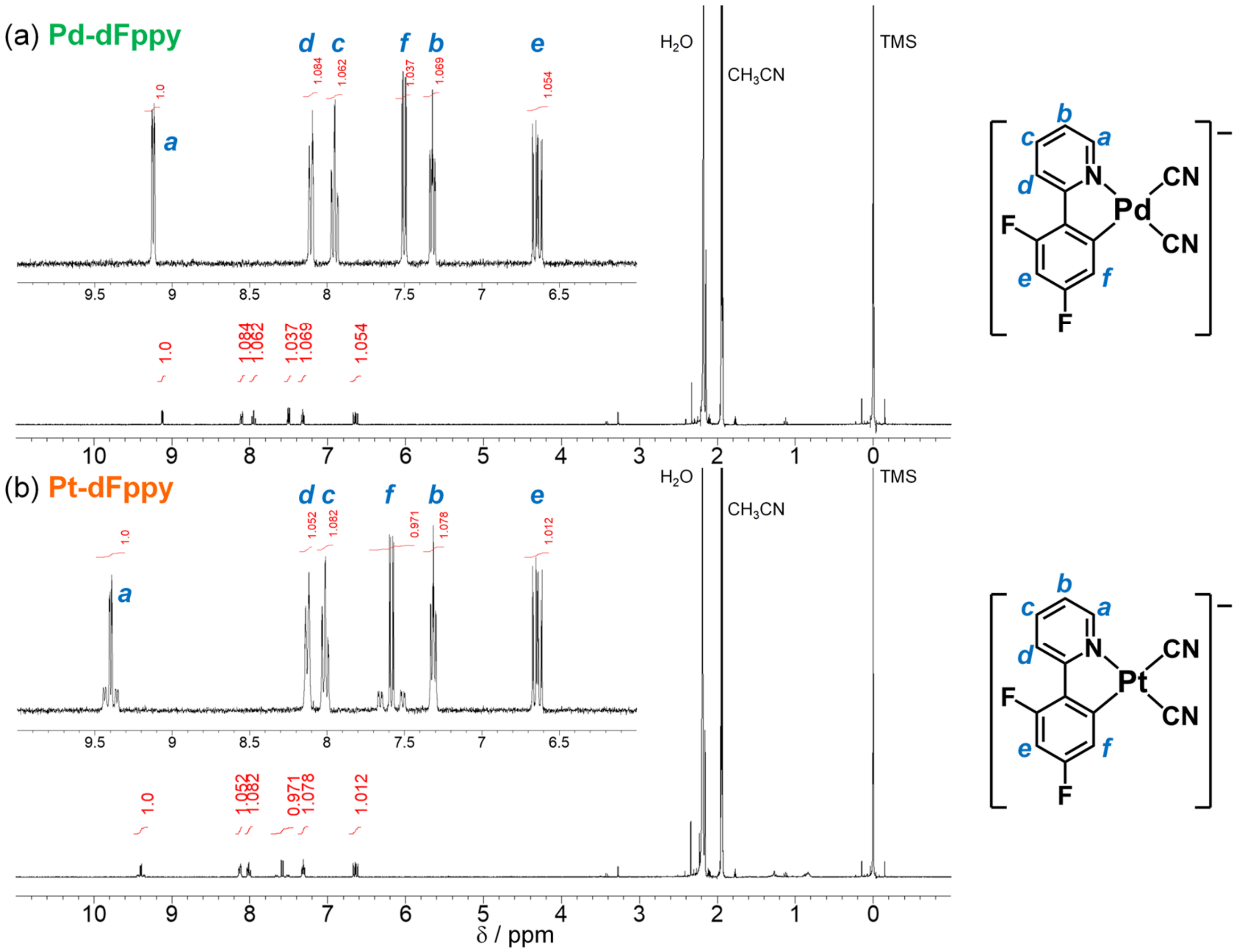

Fig. S17 ^{1}H NMR spectra (400 MHz) of (a) **Pd-dFppy** and (b) **Pt-dFppy** in CD_3CN at 298 K.

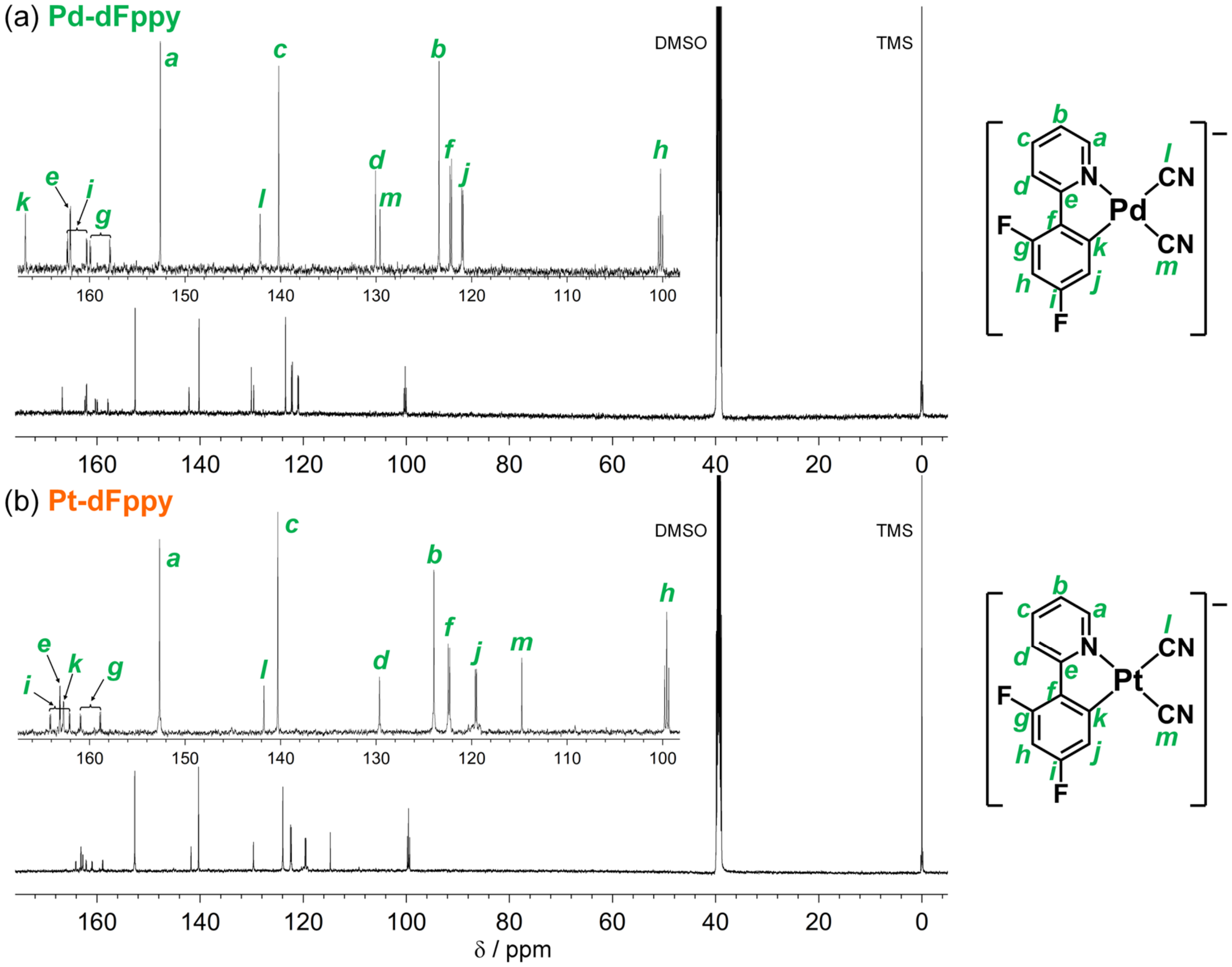

Fig. S18 ^{13}C NMR spectra (126 MHz) of (a) **Pd-dFppy** and (b) **Pt-dFppy** in DMSO-d_6 at 298 K.

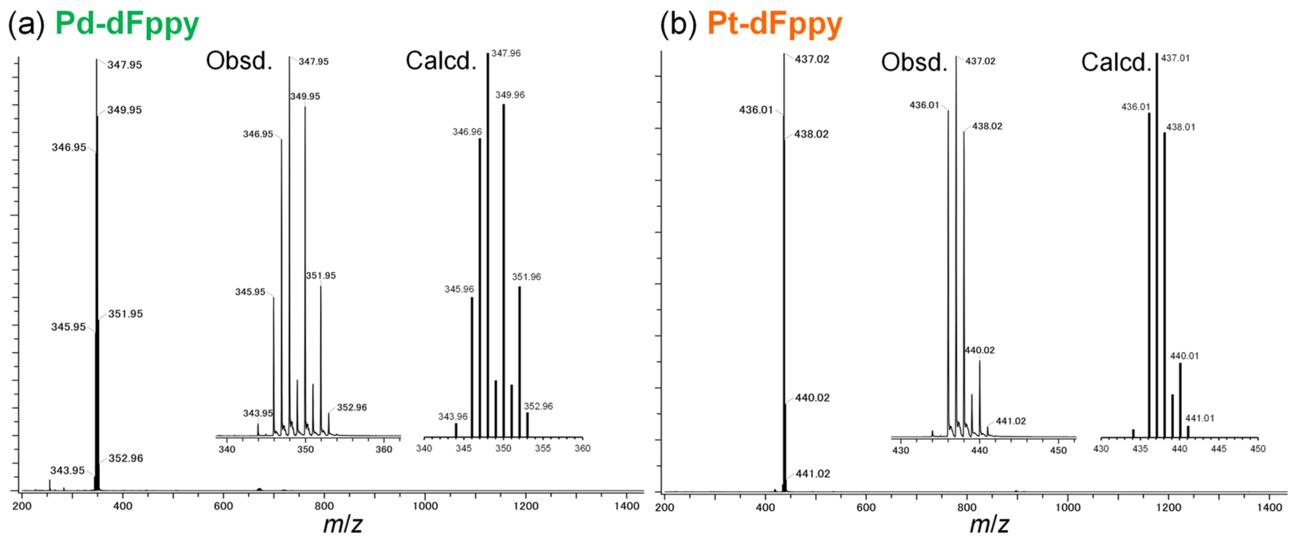

Fig. S19 ESI-TOF mass spectra of (a) **Pd-dFppy** and (b) **Pt-dFppy** in MeOH.

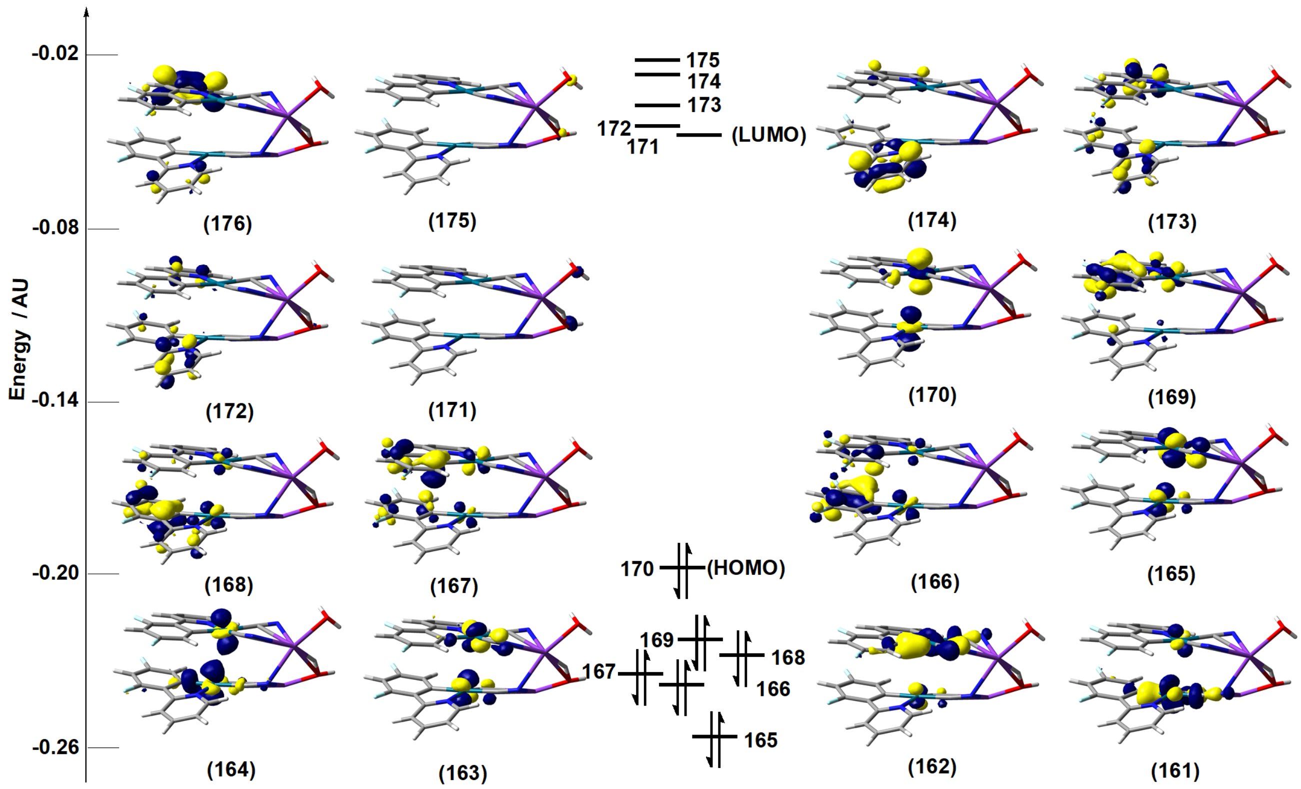

Fig. S20 Kohn-Sham frontier orbitals of the Pd2 system. (isovalue = 0.035)

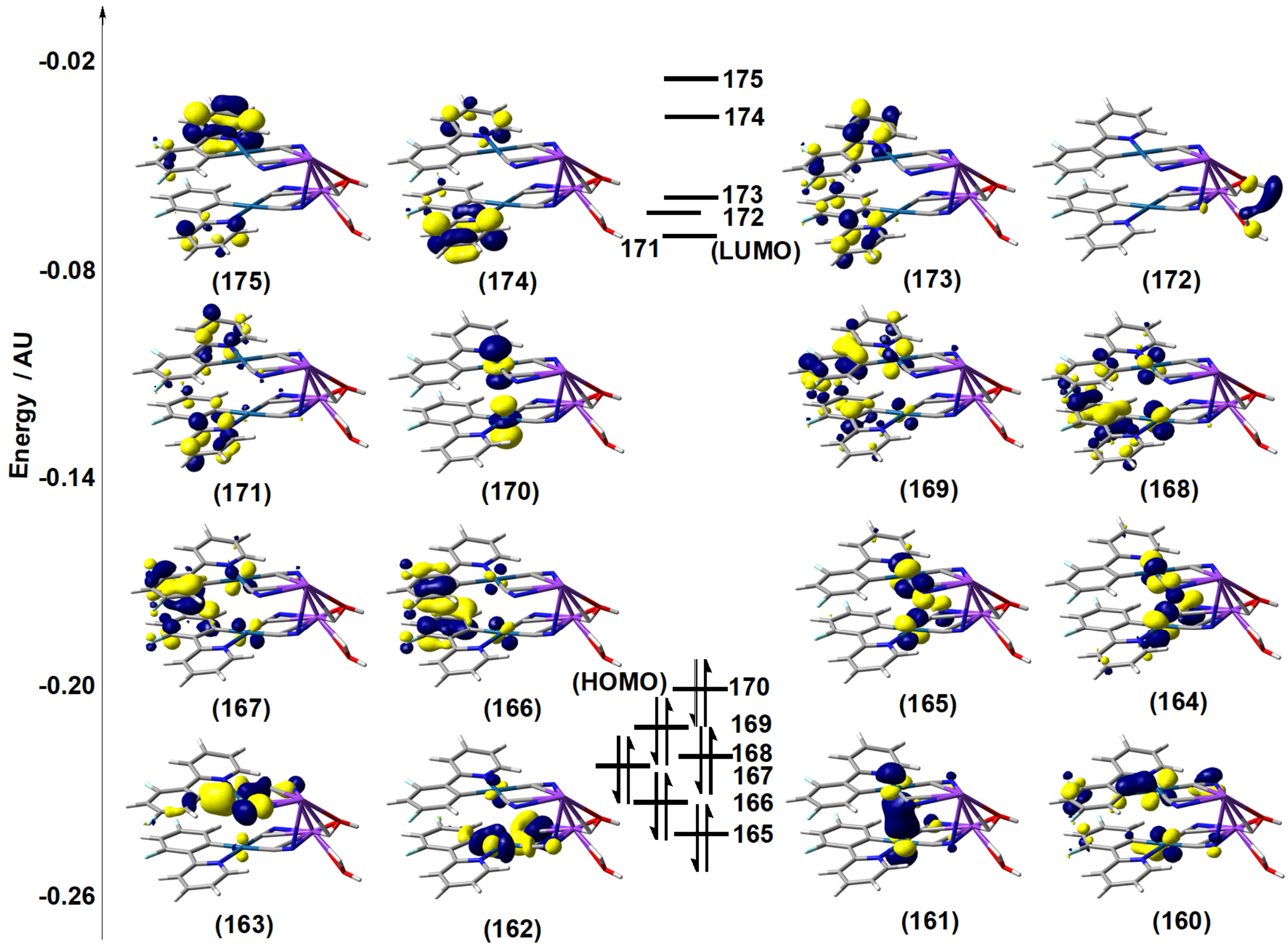

Fig. S21 Kohn-Sham frontier orbitals of the Pt2 system. (isovalue = 0.035)

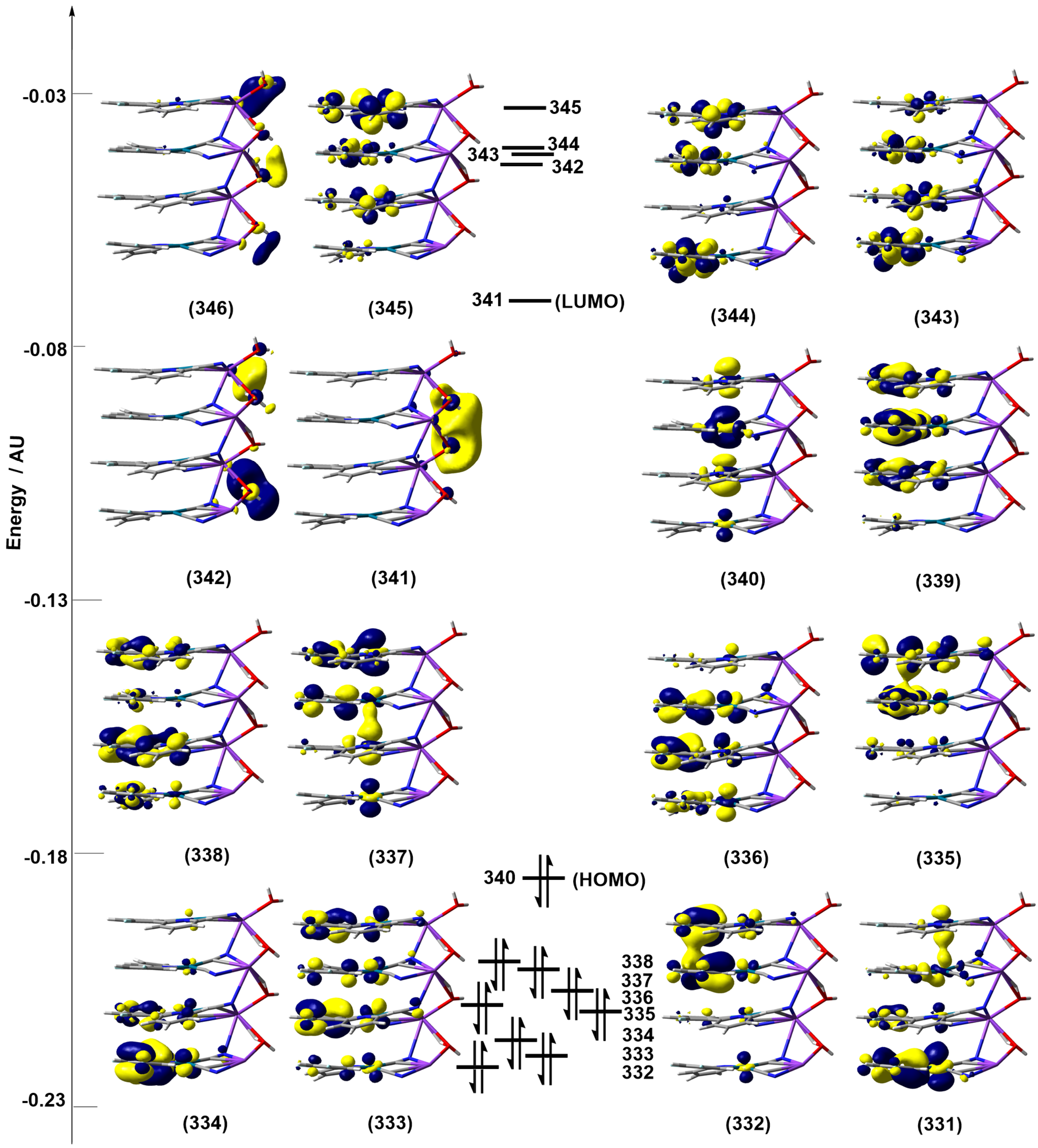

Fig. S22 Kohn-Sham frontier orbitals of the Pd4 system.

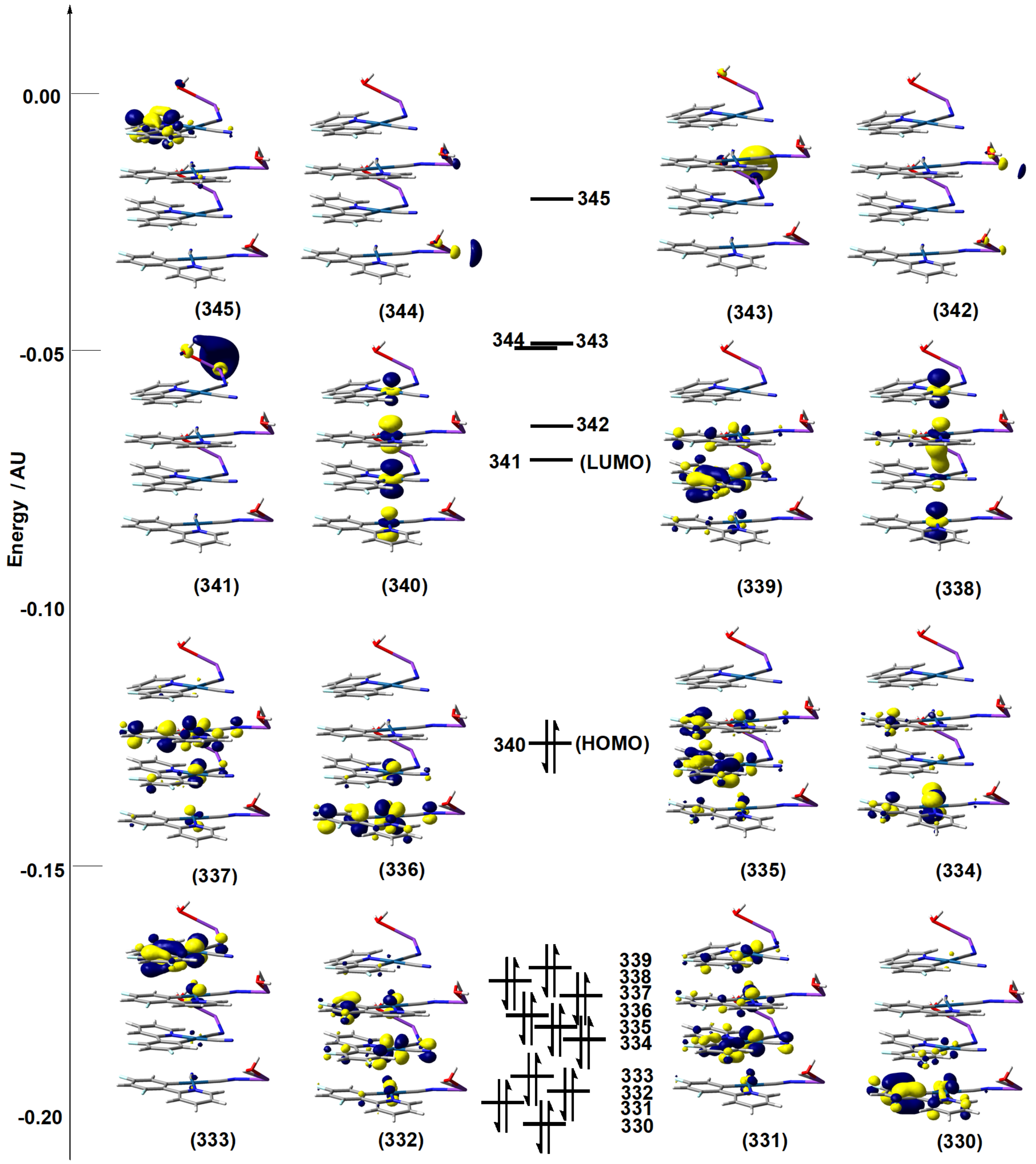

Fig. S23 Kohn-Sham frontier orbitals of the Pt4 system.

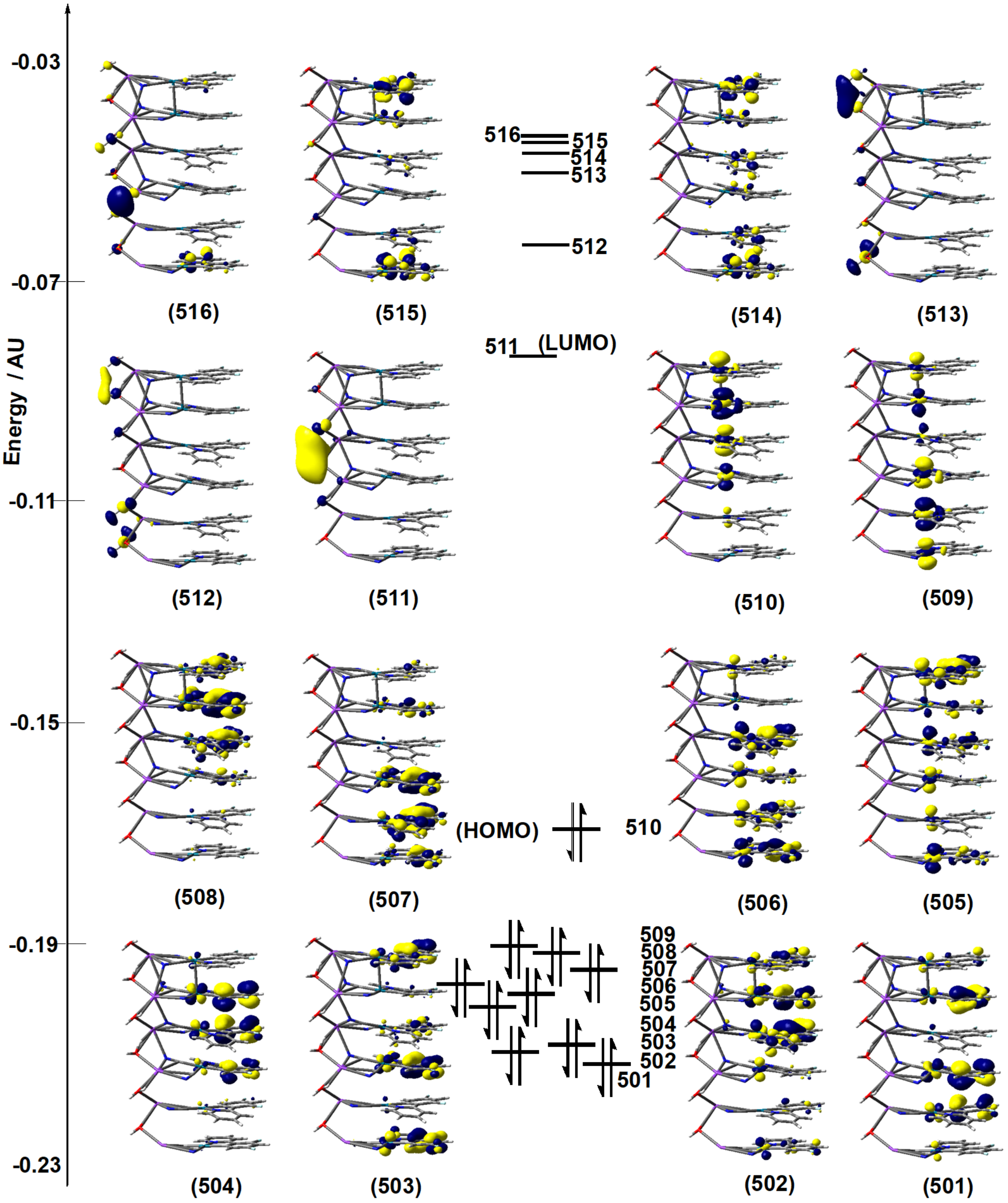

Fig. S24 Kohn-Sham frontier orbitals of the Pd6 system.

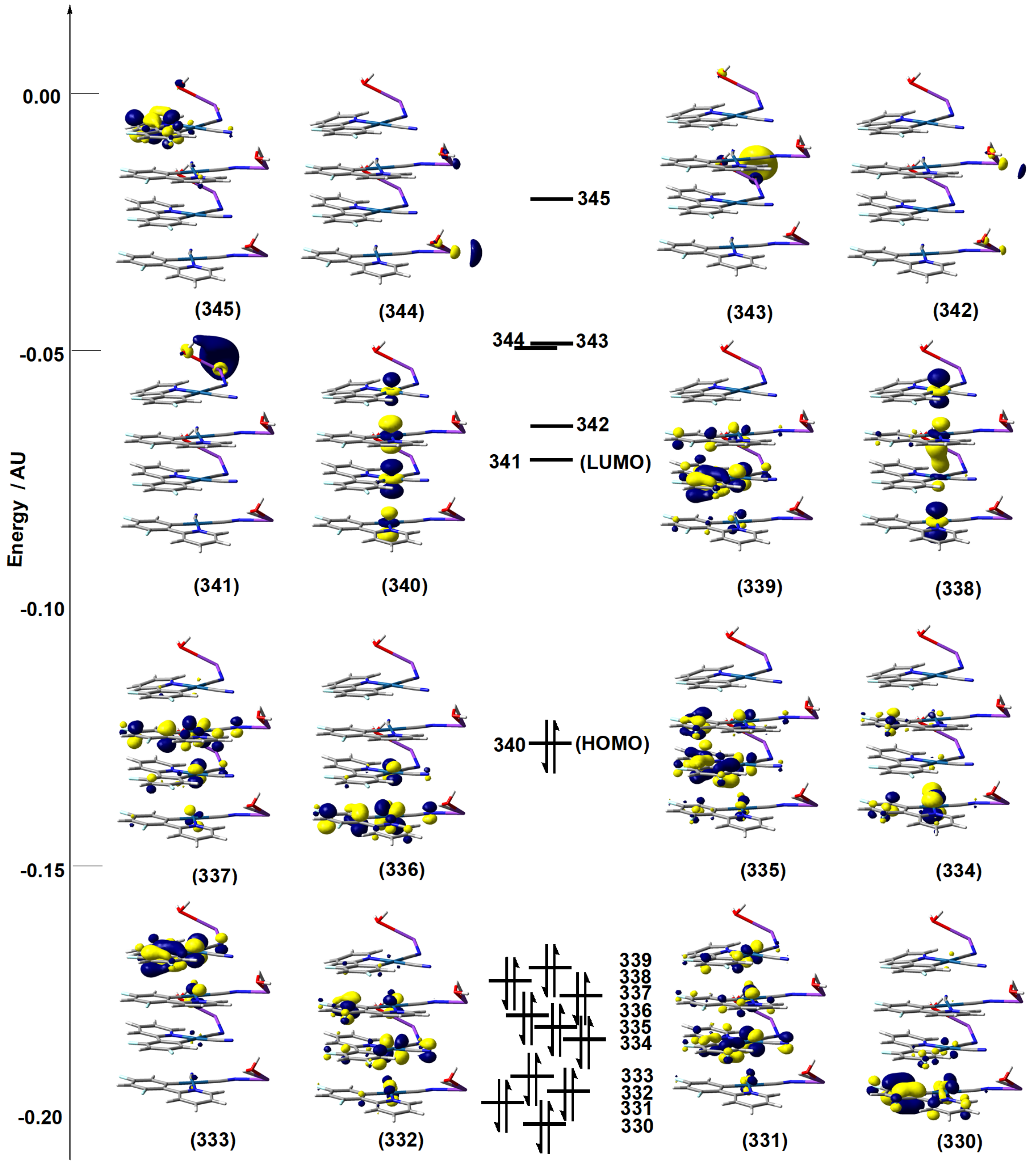

Fig. S25 Kohn-Sham frontier orbitals of the Pt6 system.

Table S1 The metal···metal distances of the optimized structure, computed HOMO-LUMO gaps and key vertical singlet excitations. The computed HOMO-LUMO gap of the Pd systems follow the order of is Pd2 (4.10 eV) > Pd4 (2.85 eV) > Pd6 (2.49 eV). Thus, the HOMO-LUMO gap decreases with the number of Pd complexes in the chain. Similar tendency can be found for the Pt systems; Pt2 (3.80 eV) > Pt4 (2.27 eV) > Pt6 (1.77 eV), where the HOMO-LUMO gap decreases rapidly with the number of Pt complexes in the chain. Kohn-Sham frontier orbitals of the M2, M4, and M6 are shown in Figs. S20-S25.

	**M···M length / Å**	**HOMO-LUMO gap / eV**	**Key vertical excitation / nm**
Pd2	3.22	4.10	389 ($f = 0.05$)
Pd4	3.37, 3.26, 3.66	2.85	419 ($f = 0.16$)
Pd6	3.48, 3.70, 3.38, 3.27, 3.02	2.49	458 ($f = 0.25$)
Pt2	3.22	3.80	426 ($f = 0.07$)
Pt4	3.28, 3.21, 3.31	2.27	468 ($f = 0.12$)
Pt6	3.27, 3.37, 3.27, 3.43, 3.34	1.77	568 ($f = 0.50$)

Table S2 Optimized fitting parameters for the temperature-dependence of emission lifetimes.

Complex	k_{I} [a] / s^{-1}	k_{II} [a] / s^{-1}	k_{III} [a] / s^{-1}	$\Delta E_{\mathrm{I\text{-}II}}$ [b] / cm^{-1}	$\Delta E_{\mathrm{I\text{-}III}}$ [b] / cm^{-1}
**Pd-dFppy**	1.35×10^4 (= $k_{\mathrm{I}} + k_{\mathrm{II}}$) [c]		2.35×10^5	0 (fixed)[c]	3.88
**Pt-dFppy**	1.79×10^5	9.83×10^5	4.35×10^6	14.3	79.7

[a] The decay rate constants of individual sublevels of the lowest excited triplet state (T_1). [b] The energy separations between the sublevels. [c] The $\Delta E_{\mathrm{I\text{-}II}}$ value for **Pd-dFppy** was found to be very small (< 1 cm^{-1}), thus k_{I} and k_{II} could not be separated. Therefore, $\Delta E_{\mathrm{I\text{-}II}}$ was approximated as 0 for the fitting.

Table S3 Fitting data for emission decay curves of **Pd-dFppy**.

Temperature / K	τ_1 [a] / μs (A_1 [b])	τ_2 [a] / μs (A_2 [b])	τ_{av} [c] / μs
3.8	2.08 (0.110)	32.9 (0.890)	32.6
5	2.18 (0.086)	26.0 (0.914)	25.8
6	2.42 (0.081)	22.6 (0.919)	22.4
8	2.84 (0.080)	18.8 (0.920)	18.6
10	3.53 (0.089)	17.1 (0.911)	16.9
15	3.78 (0.087)	15.2 (0.913)	15.0
20	4.01 (0.068)	14.2 (0.932)	14.0
30	9.57 (0.691)	18.8 (0.309)	13.9
50	8.68 (0.377)	14.6 (0.623)	13.0
77	8.61 (0.476)	16.5 (0.524)	14.0
100	7.41 (0.297)	14.6 (0.703)	13.3
130	7.45 (0.648)	17.8 (0.352)	11.3
150	4.71 (0.705)	10.0 (0.295)	7.22
180	0.516 (0.219)	2.24 (0.781)	2.13
210	0.108 (0.286)	0.942 (0.714)	0.906
240	0.0414 (0.341)	0.559 (0.659)	0.540
270	0.0149 (0.358)	0.280 (0.642)	0.272
300	0.00835 (0.442)	0.149 (0.558)	0.143

[a] Emission lifetimes. [b] Pre-exponential factors. [c] Averaged emission lifetimes.

Table S4 Fitting data for emission decay curves of **Pt-dFppy**.

Temperature / K	τ_1 [a] / µs (A_1 [b])	τ_2 [a] / µs (A_2 [b])	τ_{av} [c] / µs
4	3.35 (0.771)	8.27 (0.229)	5.43
10	1.40 (0.230)	3.93 (0.770)	3.69
20	0.668 (0.190)	2.61 (0.810)	2.50
30	0.608 (0.231)	2.07 (0.769)	1.95
40	0.324 (0.234)	1.74 (0.766)	1.67
50	0.237 (0.275)	1.44 (0.725)	1.37
60	0.210 (0.349)	1.26 (0.651)	1.17
70	0.201 (0.420)	1.12 (0.580)	1.02
77	0.166 (0.473)	1.03 (0.526)	0.916
100	0.122 (0.592)	0.756 (0.408)	0.636
130	0.104 (0.740)	0.527 (0.260)	0.375
150	0.104 (0.761)	0.335 (0.239)	0.220
180	0.0645 (0.859)	0.257 (0.141)	0.141
210	0.0420 (0.879)	0.170 (0.121)	0.0880
240	0.0341 (0.915)	0.157 (0.085)	0.0710
270	0.0138 (0.746)	0.0409 (0.254)	0.0274
300	0.00864 (0.755)	0.0240 (0.245)	0.0159

[a] Emission lifetimes. [b] Pre-exponential factors. [c] Averaged emission lifetimes.

Supplementary tables for X-ray crystallography and DFT calculation

Table S5 Crystal parameters and refinement data for **Pd-dFppy** and **Pt-dFppy** at 240 K.

	**Pd-dFppy**	**Pt-dFppy**
Formula	$C_{13}H_8F_2KN_3OPd$	$C_{13}H_8F_2KN_3OPt$
Formula weight	405.72	494.41
Crystal system	Monoclinic	Monoclinic
Space group	$P2_1/c$	$P2_1/c$
a / Å	19.6838(4)	19.8227(6)
b / Å	10.2976(2)	10.2337(3)
c / Å	6.7242(2)	6.6671(2)
β / deg	94.821(2)	94.869(3)
V / Å^3	1358.15(6)	1347.60(7)
T / K	240	240
Z	4	4
D_{calc} / g cm^{-3}	1.984	2.437
Reflections collected	24978	7394
Unique reflections	2477	2435
GOF on F^2	1.184	1.130
R_{int}	0.0467	0.0254
R_1 [a]	0.0765	0.0644
wR_2 [b] (all data)	0.1732	0.1462
CCDC No.	2357833	2357839

[a] $R_1 = \Sigma||F_o|-|F_c|| /\Sigma|F_o|$. [b] $wR_2 = [\Sigma w(F_o^2 - F_c^2)/ \Sigma w(F_o)^2]^{1/2}$, $w = [\sigma_c^2(F_o^2) + (xP)^2 + yP]^{-1}$, $P = (F_o^2 - 2F_c^2)/3$.

Table S6 Selected interatomic distances (Å) and angles (degree) of **Pd-dFppy** and **Pt-dFppy** at 240 K.

**Pd-dFppy**		**Pt-dFppy**	
Distances / Å			
Pd1-N1/C11	2.05(1)	Pt1-N1	2.055(7)
Pd1-C11/N1	2.03(1)	Pt1-C11	2.045(7)
Pd1-C12	2.00(1)	Pt1-C12	2.02(2)
Pd1-C13	1.99(1)	Pt1-C13	2.00(2)
Pd1···Pd1'	3.363(1)	Pt1···Pt1'	3.3341(9)
Angles / degree			
N1-Pd1-C11	81.2(5)	N1-Pt1-C11	80.1(4)
C11/N1A-Pd1-C13	93.3(5)	C11-Pt1-C13	94.2(5)
C13-Pd1-C12	91.8(5)	C13-Pt1-C12	91.8(6)
C12-Pd1-N1/C11A	93.7(5)	C12-Pt1-N1	93.8(5)
N1/C11A-Pd1-C13	174.5(5)	N1-Pt1-C13	174.4(5)
C11/N1A-Pd1-C12	174.9(5)	C11-Pt1-C12	173.9(5)
Pd1'···Pd1···Pd1''	177.41(3)	Pt1'···Pt1···Pt1''	177.96(3)

Table S7 Crystal parameters and refinement data for **Pd-dFppy** at various temperatures during 100-210 K.

T / K	100	130	150	180	210
Formula	$C_{13}H_8F_2KN_3OPd$	$C_{13}H_8F_2KN_3OPd$	$C_{13}H_8F_2KN_3OPd$	$C_{13}H_8F_2KN_3OPd$	$C_{13}H_8F_2KN_3OPd$
Formula weight	405.72	405.72	405.72	405.72	405.72
Crystal system	Monoclinic	Monoclinic	Monoclinic	Monoclinic	Monoclinic
Space group	$P2_1/c$	$P2_1/c$	$P2_1/c$	$P2_1/c$	$P2_1/c$
a / Å	19.690(1)	19.6867(9)	19.6881(9)	19.6862(9)	19.6749(7)
b / Å	10.2547(5)	10.2626(4)	10.2653(4)	10.2795(5)	10.2964(4)
c / Å	6.6369(3)	6.6569(3)	6.6697(3)	6.6908(3)	6.7069(2)
β / deg	94.829(5)	94.892(4)	94.868(4)	94.908(4)	94.967(3)
V / Å^3	1335.3(1)	1340.0(1)	1343.1(1)	1349.0(1)	1353.59(8)
Z	4	4	4	4	4
D_{calc} / g cm^{-3}	2.018	2.011	2.006	1.998	1.991
Reflections collected	6944	6829	7289	6890	7412
Unique reflections	2410	2427	2435	2435	2454
GOF on F^2	1.191	1.220	1.206	1.244	1.213
R_{int}	0.0496	0.0442	0.0524	0.0437	0.0385
R_1 ^{a}	0.1091	0.0982	0.0943	0.0883	0.0840
wR_2 ^{b} (all data)	0.2357	0.2189	0.2122	0.1976	0.1905
CCDC No.	2357838	2357837	2357836	2357835	2357834
Pd1···Pd1' / Å	3.319(2)	3.329(1)	3.335(1)	3.346(1)	3.355(1)
Pd1'···Pd1···Pd1" / deg	178.39(5)	177.94(4)	177.76(4)	177.18(4)	176.57(4)

^{a} $R_1 = \Sigma||F_o|-|F_c|| / \Sigma|F_o|$. ^{b} $wR_2 = [\Sigma w(F_o^2 - F_c^2) / \Sigma w(F_o)^2]^{1/2}$, $w = [\sigma_c^2(F_o^2) + (xP)^2 + yP]^{-1}$, $P = (F_o^2 - 2F_c^2)/3$.

Table S8 Crystal parameters and refinement data for **Pt-dFppy** at various temperatures during 100-210 K.

T / K	100	130	150	180	210
Formula	$C_{13}H_6F_2KN_3OPt$	$C_{13}H_6F_2KN_3OPt$	$C_{13}H_6F_2KN_3OPt$	$C_{13}H_6F_2KN_3OPt$	$C_{13}H_6F_2KN_3OPt$
Formula weight	494.41	494.41	494.41	494.41	494.41
Crystal system	Monoclinic	Monoclinic	Monoclinic	Monoclinic	Monoclinic
Space group	$P2_1/c$	$P2_1/c$	$P2_1/c$	$P2_1/c$	$P2_1/c$
a / Å	19.8515(6)	19.829(1)	19.813(1)	19.841(1)	19.830(1)
b / Å	10.2075(3)	10.1978(7)	10.2058(8)	10.2132(6)	10.2203(5)
c / Å	6.5701(2)	6.5967(4)	6.6070(4)	6.6271(4)	6.6472(4)
β / deg	94.710(3)	94.736(7)	94.742(7)	94.798(5)	94.829(6)
V / Å^3	1326.83(7)	1329.39(15)	1331.4(2)	1338.2(1)	1342.4(1)
Z	4	4	4	4	4
D_{calc} / g cm^{-3}	2.475	2.470	2.467	2.454	2.446
Reflections collected	34827	7061	6945	7017	6547
Unique reflections	2418	2407	2412	2428	2399
GOF on F^2	1.118	1.104	1.088	1.076	1.116
R_{int}	0.0717	0.0322	0.0464	0.0402	0.0271
R_1 ^{a}	0.0790	0.0852	0.0828	0.0740	0.0678
wR_2 ^{b} (all data)	0.1576	0.2080	0.2385	0.1722	0.1521
CCDC No.	2357844	2357843	2357842	2357841	2357840
Pt1···Pt1' / Å	3.2851(9)	3.298(1)	3.304(1)	3.314(1)	3.3239(9)
Pt1'···Pt1···Pt1" / deg	179.66(3)	179.06(3)	178.97(3)	178.64(3)	178.36(3)

^{a} $R_1 = \Sigma||F_o|-|F_c|| / \Sigma|F_o|$. ^{b} $wR_2 = [\Sigma w(F_o^2 - F_c^2) / \Sigma w(F_o)^2]^{1/2}$, $w = [\sigma_c^2(F_o^2) + (xP)^2 + yP]^{-1}$, $P = (F_o^2 - 2F_c^2)/3$.

Table S9 Cartesian coordinates for the optimized S_0 state structure of $[K(H_2O)][Pd(CN)_2(dFppy)]$.

Pd	1.147381	0.403304	1.633501	C	-2.980330	-0.479925	3.997485
O	6.574683	-0.009089	-1.037202	H	-3.928275	-0.626336	4.514595
N	3.845880	1.055064	0.043794	C	-2.001790	-1.466076	3.972959
N	2.718305	-2.239233	2.082710	C	-0.788048	-1.293444	3.317352
C	0.510115	3.215321	0.681909	H	-0.063073	-2.108577	3.336928
H	1.508396	3.118584	0.249815	C	2.825883	0.915220	0.598805
C	-0.243674	4.370828	0.526120	C	2.056158	-1.283737	1.960697
H	0.158148	5.212188	-0.039998	F	-2.247395	-2.612436	4.603379
C	-1.507009	4.409974	1.110731	F	-3.665330	1.645918	3.371665
H	-2.136578	5.298111	1.014947	N	0.066786	2.156147	1.365287
C	-1.969920	3.310787	1.821643	C	-0.519487	-0.095133	2.652821
H	-2.952845	3.323719	2.284700	H	6.055282	0.123848	-1.833466
C	-1.161881	2.169400	1.945596	H	7.365714	-0.509323	-1.250971
C	-1.502468	0.936167	2.659369	K	5.070896	-1.408601	0.825198
C	-2.709181	0.707228	3.336677				

Table S10 Cartesian coordinates for the optimized S_0 state structure of $[K(H_2O)][Pt(CN)_2(dFppy)]$.

Pt	1.523437	0.289440	-2.155194	C	-0.350216	-0.327804	-2.158564
F	-2.605858	-3.176932	-1.970911	C	-1.315741	0.763487	-2.297708
F	-3.616471	1.347765	-2.447171	C	-2.670446	0.414795	-2.319528
N	0.643384	2.105585	-2.349397	C	-3.102355	-0.899707	-2.210335
C	1.273130	3.309016	-2.434340	H	-4.169649	-1.124655	-2.231067
H	2.362718	3.279301	-2.390852	C	-2.154731	-1.931048	-2.073200
C	0.602090	4.499084	-2.566631	C	-0.806116	-1.664023	-2.045832
H	1.178263	5.423932	-2.628212	H	-0.098234	-2.486801	-1.938423
C	-0.817544	4.508155	-2.620512	C	3.449485	1.013017	-2.151554
H	-1.375442	5.438772	-2.725158	C	2.258065	-1.533071	-1.964033
C	-1.475491	3.287953	-2.534445	K	5.211884	-1.309658	-1.953477
H	-2.562202	3.246499	-2.570625	O	6.731287	-0.323619	0.203857
C	-0.764730	2.093261	-2.399810	H	7.581211	-0.060894	0.636135
N	4.584778	1.297771	-2.139265	H	6.740805	0.121142	-0.669093
N	2.762884	-2.583675	-1.853785				

Table S11 Cartesian coordinates for the optimized T_1 state structure of $[K(H_2O)][Pd(CN)_2(dFppy)]$.

Pd	1.146125	0.408067	1.637021	C	-3.003968	-0.441656	4.005921
O	6.574683	-0.009089	-1.037202	H	-3.948489	-0.597892	4.527937
N	3.84588	1.055064	0.043794	C	-2.011000	-1.448826	3.981609
N	2.718305	-2.239233	2.08271	C	-0.787842	-1.275015	3.318136
C	0.513224	3.201959	0.695354	H	-0.067377	-2.094606	3.340045
H	1.510403	3.123782	0.254076	C	2.825742	0.917946	0.599448
C	-0.251282	4.374184	0.539229	C	2.055288	-1.283918	1.963139
H	0.160554	5.209402	-0.028442	F	-2.268682	-2.589895	4.615804
C	-1.544552	4.434292	1.129907	F	-3.701906	1.683202	3.388481
H	-2.160057	5.329764	1.025248	N	0.092240	2.148365	1.365069
C	-2.003975	3.352369	1.831114	C	-0.505005	-0.093939	2.653892
H	-2.987342	3.365892	2.294937	H	6.055282	0.123848	-1.833466
C	-1.19209	2.167984	1.971007	H	7.365714	-0.509323	-1.250971
C	-1.514787	0.988038	2.653119	K	5.070896	-1.408601	0.825198
C	-2.755053	0.735489	3.359731				

Table S12 Cartesian coordinates for the optimized T_1 state structure of $[K(H_2O)][Pt(CN)_2(dFppy)]$.

Pt	1.548644	0.276124	-2.161219	C	-0.363581	-0.343815	-2.181448
F	-2.690772	-3.127191	-2.019079	C	-1.321951	0.775656	-2.324063
F	-3.633252	1.422789	-2.480693	C	-2.728097	0.444357	-2.352114
N	0.639020	2.104125	-2.363602	C	-3.179015	-0.840630	-2.251659
C	1.260803	3.270452	-2.441679	H	-4.247743	-1.056097	-2.276036
H	2.351738	3.237517	-2.392525	C	-2.230285	-1.883601	-2.115387
C	0.588493	4.492224	-2.579496	C	-0.855583	-1.638893	-2.081399
H	1.168591	5.413984	-2.637312	H	-0.178996	-2.488695	-1.974182
C	-0.834036	4.497725	-2.640248	C	3.451916	1.000218	-2.15253
H	-1.381469	5.435715	-2.747510	C	2.258875	-1.532774	-1.967709
C	-1.500930	3.305381	-2.561228	K	5.211884	-1.309658	-1.953477
H	-2.586909	3.271091	-2.603722	O	6.731287	-0.323619	0.203857
C	-0.783707	2.066901	-2.419519	H	7.581211	-0.060894	0.636135
N	4.584778	1.297771	-2.139265	H	6.740805	0.121142	-0.669093
N	2.762884	-2.583675	-1.853785				

Table S13 Cartesian coordinates for the optimized structure of Pd monomer system (only the ONIOM high-layer).

Pd	1.147381	0.403304	1.633501	C	-2.980330	-0.479925	3.997485
O	6.574683	-0.009089	-1.037202	H	-3.928275	-0.626336	4.514595
N	3.845880	1.055064	0.043794	C	-2.001790	-1.466076	3.972959
N	2.718305	-2.239233	2.082710	C	-0.788048	-1.293444	3.317352
C	0.510115	3.215321	0.681909	H	-0.063073	-2.108577	3.336928
H	1.508396	3.118584	0.249815	C	2.825883	0.915220	0.598805
C	-0.243674	4.370828	0.526120	C	2.056158	-1.283737	1.960697
H	0.158148	5.212188	-0.039998	F	-2.247395	-2.612436	4.603379
C	-1.507009	4.409974	1.110731	F	-3.665330	1.645918	3.371665
H	-2.136578	5.298111	1.014947	N	0.066786	2.156147	1.365287
C	-1.969920	3.310787	1.821643	C	-0.519487	-0.095133	2.652821
H	-2.952845	3.323719	2.284700	H	6.055282	0.123848	-1.833466
C	-1.161881	2.169400	1.945596	H	7.365714	-0.509323	-1.250971
C	-1.502468	0.936167	2.659369	K	5.070896	-1.408601	0.825198
C	-2.709181	0.707228	3.336677				

Table S14 Cartesian coordinates for the optimized structure of Pt monomer system (only the ONIOM high-layer).

Pt	1.553668	0.271829	-2.155134	C	-0.378454	-0.339759	-2.174061
F	-2.668393	-3.142017	-2.008588	C	-1.313809	0.727583	-2.307635
F	-3.596787	1.395553	-2.458029	C	-2.682779	0.424338	-2.333800
N	0.611239	2.115861	-2.356795	C	-3.160677	-0.871674	-2.234726
C	1.259094	3.285108	-2.434876	H	-4.230869	-1.075375	-2.258090
H	2.348160	3.231071	-2.386596	C	-2.225337	-1.891749	-2.105043
C	0.597053	4.496230	-2.568766	C	-0.857600	-1.648751	-2.073597
H	1.168148	5.423534	-2.628280	H	-0.178244	-2.496160	-1.969180
C	-0.794631	4.480084	-2.622667	C	3.452598	0.997918	-2.150267
H	-1.358411	5.410195	-2.727697	C	2.260062	-1.532017	-1.965711
C	-1.469246	3.270097	-2.541651	K	5.211884	-1.309658	-1.953477
H	-2.554550	3.236295	-2.581419	O	6.731287	-0.323619	0.203857
C	-0.749050	2.073084	-2.406418	H	7.581211	-0.060894	0.636135
N	4.584778	1.297771	-2.139265	H	6.740805	0.121142	-0.669093
N	2.762884	-2.583675	-1.853785				

Table S15 Cartesian coordinates for the optimized structure of Pd2 system (only the ONIOM high-layer).

Pd	0.618998	-0.386835	-1.695318	N	2.718305	-2.239233	2.082710
N	3.471795	-1.505289	-2.655103	C	0.753947	3.294540	1.388116
N	1.949854	2.120523	-2.920291	H	1.677400	3.233092	0.812508
C	0.483648	-3.258723	-0.894765	C	0.266231	4.493085	1.888105
H	1.544832	-3.195018	-1.130247	H	0.805683	5.423256	1.715115
C	-0.091747	-4.414334	-0.400091	C	-0.905391	4.454273	2.635945
H	0.518654	-5.296828	-0.218454	H	-1.289651	5.352194	3.107568
C	-1.447143	-4.379676	-0.104157	C	-1.582950	3.259105	2.815092
H	-1.902336	-5.232879	0.378549	H	-2.502574	3.215502	3.393370
C	-2.199087	-3.238574	-0.338328	C	-1.040149	2.087007	2.282689
H	-3.257754	-3.210738	-0.096442	C	-1.586627	0.743651	2.443831
C	-1.550516	-2.087862	-0.797482	C	-2.914274	0.464790	2.784852
C	-2.124357	-0.747154	-0.921166	C	-3.395444	-0.830766	2.858298
C	-3.457037	-0.413940	-0.651057	H	-4.446439	-1.028793	3.052217
C	-3.914777	0.894022	-0.674527	C	-2.493708	-1.861163	2.636214
H	-4.961055	1.121241	-0.477093	C	-1.157014	-1.640765	2.323123
C	-2.994954	1.893889	-0.964124	H	-0.502871	-2.499162	2.163379
C	-1.661075	1.622240	-1.249722	C	2.820796	0.896348	0.580196
H	-0.993182	2.450855	-1.490393	C	1.988737	-1.384390	1.769122
C	2.414867	-1.180794	-2.276806	F	-2.950282	-3.109643	2.697352
C	1.340003	1.268244	-2.411259	F	-3.779539	1.457737	3.010878
F	-3.415626	3.153937	-0.972143	N	0.132509	2.133043	1.604496
F	-4.346474	-1.364474	-0.347199	C	-0.702241	-0.332133	2.177099
N	-0.223910	-2.142304	-1.068756	H	5.477673	-1.479836	-4.397779
C	-1.220141	0.301379	-1.244631	H	6.815036	-0.822295	-3.911224
O	5.956058	-0.652719	-4.304679	H	6.055282	0.123848	-1.833466
O	6.574683	-0.009089	-1.037202	H	7.365714	-0.509323	-1.250971
Pd	1.065641	0.286210	1.421560	K	4.516818	1.068297	-2.674951
N	3.845880	1.055064	0.043794	K	5.070896	-1.408601	0.825198

Table S16 Cartesian coordinates for the optimized structure of Pd4 system (only the ONIOM high-layer).

Pd	0.576751	-0.238648	-1.660872	C	-1.002747	-2.591322	6.302545
N	3.241625	-1.226918	-3.150949	H	-2.020577	-2.475275	6.664547
N	1.952289	2.511862	-1.547800	C	-0.310311	-1.485412	5.797033
C	0.301228	-3.200971	-1.348640	C	-0.794403	-0.112345	5.774071
H	1.375055	-3.142799	-1.513693	C	-2.130350	0.250039	5.971794
C	-0.374226	-4.404905	-1.268465	C	-2.543826	1.569291	5.967696
H	0.148746	-5.353778	-1.370841	H	-3.593184	1.832764	6.087219
C	-1.751035	-4.349995	-1.110149	C	-1.567565	2.534499	5.778224
H	-2.327760	-5.255296	-1.202144	C	-0.223513	2.233012	5.578513
C	-2.397138	-3.144009	-0.909034	H	0.481362	3.052168	5.427787
H	-3.465944	-3.102853	-0.731293	C	3.757635	-0.67434	4.361942
C	-1.655848	-1.963170	-0.994382	C	2.939723	1.834457	5.137423
C	-2.169312	-0.601452	-0.882048	F	-1.968157	3.804953	5.759694
C	-3.500294	-0.266505	-0.614328	F	-3.072251	-0.682299	6.160048
C	-3.951868	1.043528	-0.616334	N	0.971308	-1.624716	5.385256
H	-5.004205	1.271075	-0.462949	C	0.183651	0.897921	5.552079
C	-3.013327	2.036892	-0.849658	K	4.516819	1.068374	-2.674943
C	-1.670216	1.766850	-1.071946	O	5.955983	-0.652685	-4.304592
H	-0.989039	2.600602	-1.247082	K	5.737339	1.535006	3.921054
C	2.291614	-0.957093	-2.517743	O	7.176503	-0.186053	2.291405
C	1.340996	1.532920	-1.713818	K	3.85037	-1.875234	-5.770805
F	-3.434241	3.303318	-0.896051	O	5.354231	-0.475629	-7.633196
F	-4.409678	-1.217602	-0.363816	K	5.07089	-1.408602	0.825192
N	-0.332888	-2.033061	-1.271790	O	6.574751	-0.008997	-1.037199
C	-1.233392	0.443737	-1.109101	Pd	-0.079052	-0.378284	-4.961416
Pd	1.995839	0.139493	5.066379	N	2.756035	0.538754	-6.121695
N	4.750679	-0.992693	3.825470	N	1.376256	-3.118483	-5.223722
N	3.704011	2.716544	5.199463	C	-0.315781	2.638389	-4.972938
C	1.573967	-2.812372	5.418688	H	0.678553	2.548603	-5.402525
H	2.596676	-2.816279	5.052704	C	-0.852299	3.863424	-4.611352
C	0.953271	-3.939959	5.930852	H	-0.264879	4.774966	-4.721651
H	1.468830	-4.896644	5.970634	C	-2.133164	3.869534	-4.070592
C	-0.339387	-3.799779	6.416861	H	-2.560914	4.783034	-3.670644
H	-0.812591	-4.62133	6.939262	C	-2.853353	2.690419	-3.977953

(Continued)

H	-3.859538	2.687524	-3.570209	C	-1.595732	3.274803	2.668686
C	-2.239206	1.482423	-4.330159	H	-2.571471	3.252804	3.144377
C	-2.811301	0.148313	-4.182374	C	-1.006348	2.087344	2.224912
C	-4.154269	-0.104944	-3.887126	C	-1.564058	0.746380	2.374949
C	-4.653020	-1.389463	-3.765879	C	-2.900891	0.471395	2.679828
H	-5.709803	-1.564642	-3.583840	C	-3.387477	-0.820386	2.782812
C	-3.756860	-2.436315	-3.915197	H	-4.441048	-1.009692	2.974376
C	-2.410807	-2.244095	-4.200699	C	-2.483424	-1.855936	2.596066
H	-1.769501	-3.118088	-4.325999	C	-1.141295	-1.641581	2.306390
C	1.713948	0.271816	-5.658886	H	-0.491445	-2.506801	2.164735
C	0.713200	-2.165556	-5.080098	C	2.950285	0.775741	0.689363
F	-4.227993	-3.675474	-3.789635	C	1.954579	-1.476312	1.749873
F	-5.027433	0.900122	-3.736178	F	-2.942265	-3.104680	2.678949
N	-0.968659	1.487731	-4.798609	F	-3.779322	1.467706	2.859636
C	-1.921418	-0.948591	-4.366609	N	0.226356	2.115382	1.665449
Pd	1.143868	0.256605	1.506475	C	-0.668355	-0.335326	2.164382
N	3.991027	0.896202	0.161001	H	4.834680	-0.342900	-8.429429
N	2.623300	-2.405653	1.974093	H	6.145192	-0.975960	-7.846888
C	0.865098	3.270810	1.481306	H	5.477754	-1.479901	-4.397846
H	1.809559	3.200057	0.945544	H	6.815053	-0.822301	-3.911264
C	0.355260	4.477077	1.939305	H	6.055199	0.123732	-1.833433
H	0.923642	5.398610	1.821935	H	7.365701	-0.509335	-1.250991
C	-0.881109	4.457044	2.573046	H	6.700256	-1.014674	2.201381
H	-1.281118	5.352385	3.035866	H	8.037175	-0.352541	2.682735

Table S17 Cartesian coordinates for the optimized structure of Pd6 system (only the ONIOM high-layer).

Pd	-0.483567	-0.543532	-7.915191	C	-2.201288	-3.219178	-0.277922
N	2.331964	-2.003739	-8.340894	H	-3.222483	-3.178800	0.090527
N	0.809971	2.305875	-8.363758	C	-1.601186	-2.071336	-0.808803
C	-0.865570	-3.524590	-7.647058	C	-2.172074	-0.727563	-0.840138
H	0.055676	-3.584711	-7.820542	C	-3.508961	-0.414101	-0.577469
C	-1.627463	-4.704343	-7.431662	C	-3.986985	0.886966	-0.597617
H	-1.220858	-5.551270	-7.471946	H	-5.043072	1.096048	-0.441278
C	-3.011624	-4.577313	-7.148694	C	-3.067291	1.895213	-0.847147
H	-3.526475	-5.345534	-6.981557	C	-1.724890	1.644687	-1.095170
C	-3.620168	-3.306273	-7.126232	H	-1.063121	2.488142	-1.295198
H	-4.541911	-3.214113	-6.967112	C	2.286745	-0.987651	-2.550084
C	-2.837400	-2.220701	-7.347945	C	1.296302	1.462690	-1.741261
C	-3.313776	-0.791694	-7.376220	F	-3.501747	3.157244	-0.887799
C	-4.608778	-0.452420	-7.167846	F	-4.394418	-1.384256	-0.314903
C	-5.098131	0.810224	-7.157582	N	-0.320724	-2.130391	-1.246185
H	-5.997224	1.023180	-6.986293	C	-1.265727	0.329573	-1.125177
C	-4.058415	1.808657	-7.440728	Pd	1.920401	0.157360	5.187803
C	-2.757345	1.487943	-7.654070	N	4.866663	-0.880941	4.461964
H	-2.130729	2.168318	-7.824892	N	3.604991	2.752412	5.079473
C	1.293080	-1.434148	-8.200775	C	1.503029	-2.800269	5.541782
C	0.305421	1.263527	-8.197909	H	2.537502	-2.818815	5.206619
F	-4.479874	3.045486	-7.448410	C	0.869403	-3.923892	6.047238
F	-5.553798	-1.453673	-6.923088	H	1.394330	-4.874088	6.122574
N	-1.468927	-2.313427	-7.605654	C	-0.432564	-3.777231	6.505830
C	-2.357001	0.189678	-7.628759	H	-0.915731	-4.580717	7.046082
Pd	0.542017	-0.319620	-1.718921	C	-1.094409	-2.571346	6.361297
N	3.269580	-1.216425	-3.149335	H	-2.116374	-2.453317	6.708377
N	1.899132	2.450324	-1.590476	C	-0.394306	-1.472113	5.856095
C	0.389250	-3.252357	-1.149618	C	-0.889390	-0.102237	5.791191
H	1.412859	-3.187163	-1.510588	C	-2.224446	0.262891	5.985801
C	-0.127114	-4.406010	-0.586791	C	-2.643944	1.581438	5.948366
H	0.494399	-5.291700	-0.473980	H	-3.693277	1.841676	6.071017
C	-1.441723	-4.369528	-0.139690	C	-1.674080	2.544979	5.726256
H	-1.854791	-5.227147	0.376805	C	-0.331347	2.242324	5.527907

(Continued)

H	0.370137	3.058294	5.346576	C	-2.316187	-2.188131	-4.187683
C	3.759988	-0.627827	4.760262	H	-1.678646	-3.066079	-4.305952
C	2.843013	1.866847	5.110905	C	1.824129	0.308581	-5.719374
F	-2.074168	3.818331	5.679471	C	0.762028	-2.152519	-4.996360
F	-3.165043	-0.663983	6.207522	F	-4.157137	-3.614666	-3.863359
N	0.900164	-1.612080	5.485474	F	-4.921667	0.967285	-3.750012
C	0.079134	0.908985	5.540599	N	-0.863668	1.531658	-4.801895
K	3.296300	0.601742	-9.270939	C	-1.816915	-0.893634	-4.338140
O	4.735464	-1.119317	-10.900588	Pd	1.202571	0.233310	1.552621
K	4.516819	1.068374	-2.674943	N	4.015011	0.908738	0.151385
O	5.955983	-0.652685	-4.304592	N	2.845413	-2.330896	2.066263
K	5.737339	1.535006	3.921054	C	0.899154	3.241281	1.425002
O	7.176503	-0.186053	2.291405	H	1.842196	3.163208	0.888697
K	3.850370	-1.875234	-5.770805	C	0.388896	4.453157	1.866893
O	5.354231	-0.475629	-7.633196	H	0.955451	5.374126	1.741044
K	5.070890	-1.408602	0.825192	C	-0.847405	4.443846	2.500676
O	6.574751	-0.008997	-1.037199	H	-1.240965	5.345805	2.956841
K	6.291419	-0.941962	7.421288	C	-1.563963	3.264276	2.607609
O	7.795370	0.457633	5.558788	H	-2.540233	3.248035	3.082552
Pd	0.030127	-0.335787	-4.943890	C	-0.970711	2.070476	2.185560
N	2.832547	0.592121	-6.247072	C	-1.518982	0.732716	2.375679
N	1.333403	-3.168368	-5.089380	C	-2.857812	0.463754	2.671349
C	-0.220106	2.675624	-5.029907	C	-3.334901	-0.825422	2.832120
H	0.748549	2.571700	-5.507686	H	-4.390040	-1.017432	3.012576
C	-0.749783	3.908699	-4.685249	C	-2.415623	-1.856599	2.723004
H	-0.173422	4.818616	-4.846901	C	-1.069684	-1.647684	2.442800
C	-2.012730	3.927214	-4.106729	H	-0.410834	-2.513342	2.358907
H	-2.429737	4.853588	-3.725915	C	2.984062	0.769155	0.694985
C	-2.731981	2.750660	-3.970654	C	2.090418	-1.462375	1.861846
H	-3.729988	2.758256	-3.542976	F	-2.870465	-3.103573	2.861116
C	-2.127459	1.537253	-4.318958	F	-3.745937	1.460260	2.781348
C	-2.703500	0.205913	-4.162637	N	0.262775	2.087326	1.629327
C	-4.051937	-0.042992	-3.889909	C	-0.607721	-0.347800	2.232556
C	-4.563200	-1.325198	-3.788529	Pd	2.566153	0.546484	8.580610
H	-5.624954	-1.496368	-3.626349	N	5.438156	1.810128	7.951760
C	-3.669873	-2.374514	-3.933867	N	3.739764	-2.388804	8.563472

(Continued)

C	2.308172	3.547531	8.402593	C	3.279117	-1.313487	8.571383
H	3.230575	3.543242	8.224945	F	-1.573232	-2.765852	9.562101
C	1.596217	4.776571	8.438338	F	-2.459182	1.801865	9.411207
H	2.037258	5.590675	8.274174	N	1.655537	2.381804	8.621268
C	0.208873	4.750061	8.732758	C	0.664980	-0.058304	8.966832
H	-0.273360	5.555133	8.782557	H	4.259352	-1.948112	-10.990210
C	-0.451471	3.522575	8.941563	H	5.596334	-1.285419	-10.509406
H	-1.375735	3.493098	9.109754	H	4.834680	-0.342900	-8.429429
C	0.285204	2.384798	8.885668	H	6.145192	-0.975960	-7.846888
C	-0.249752	0.988258	9.068126	H	5.477754	-1.479901	-4.397846
C	-1.556990	0.737298	9.320520	H	6.815053	-0.822301	-3.911264
C	-2.097936	-0.488613	9.516382	H	6.055199	0.123732	-1.833433
H	-3.004512	-0.636577	9.714589	H	7.365701	-0.509335	-1.250991
C	-1.101131	-1.559989	9.387605	H	6.700256	-1.014674	2.201381
C	0.211390	-1.328355	9.133068	H	8.037175	-0.352541	2.682735
H	0.808903	-2.051780	9.066943	H	7.351225	1.280660	5.342167
C	4.377113	1.310917	8.171591	H	8.661832	0.648456	5.925443

Table S18 Cartesian coordinates for the optimized structure of Pt2 system (only the ONIOM high-layer).

Pt	1.530599	0.263607	-2.075417	Pt	1.743991	0.270852	-5.288809
F	-2.781505	-3.056969	-2.707132	F	-2.448336	3.760320	-5.445549
F	-3.579030	1.495724	-2.591119	F	-3.374023	-0.758604	-5.978685
N	0.580624	2.105230	-2.047468	N	0.763116	-1.538715	-5.454671
C	1.223707	3.261514	-1.868284	C	1.392554	-2.718085	-5.426689
H	2.309531	3.193733	-1.846057	H	2.437355	-2.700033	-5.116116
C	0.547652	4.454809	-1.689042	C	0.763364	-3.888665	-5.809585
H	1.078138	5.389298	-1.546226	H	1.297699	-4.835867	-5.807843
C	-0.837910	4.427079	-1.678723	C	-0.557673	-3.805316	-6.225769
H	-1.387070	5.326368	-1.436233	H	-1.034031	-4.670644	-6.663330
C	-1.510506	3.247503	-1.928920	C	-1.247862	-2.609423	-6.140125
H	-2.595299	3.216350	-1.943534	H	-2.292946	-2.544857	-6.429745
C	-0.774859	2.071440	-2.113387	C	-0.559179	-1.455437	-5.750961
N	4.585061	1.297831	-2.139256	N	4.669674	-0.908133	-4.840197
N	2.763090	-2.583849	-1.853811	N	3.342738	2.920965	-5.262196
C	-0.412746	-0.343381	-2.247543	C	-0.160556	0.949052	-5.510748
C	-1.334045	0.742622	-2.300146	C	-1.102756	-0.101866	-5.701333
C	-2.695067	0.492369	-2.512740	C	-2.463024	0.208201	-5.808950
C	-3.196015	-0.786230	-2.650086	C	-2.934379	1.505738	-5.728681
H	-4.258758	-0.965641	-2.802646	H	-4.000338	1.719934	-5.773474
C	-2.286767	-1.830122	-2.578986	C	-1.996301	2.512741	-5.548491
C	-0.920675	-1.640725	-2.385169	C	-0.631636	2.264448	-5.454127
H	-0.270766	-2.516393	-2.336353	H	0.049600	3.107493	-5.324156
C	3.451538	0.999424	-2.052294	C	3.579226	-0.533764	-5.045610
C	2.240328	-1.541822	-1.945486	C	2.634319	1.991609	-5.275276
K	5.211384	-1.309553	-1.953435	H	7.581200	-0.060800	0.636100
O	7.232706	0.300373	-3.057948	H	6.740804	0.121162	-0.669114
K	5.747001	1.617370	-5.058688	H	8.078881	0.134156	-2.575613
O	6.731311	-0.323724	0.203888	H	6.639739	0.881017	-2.540090

Table S19 Cartesian coordinates for the optimized structure of Pt4 system (only the ONIOM high-layer).

Pt	1.422361	0.204239	-1.924634	H	-3.625807	2.775751	4.650845
F	-2.904524	-3.098068	-2.462104	C	-1.820615	1.613480	4.444729
F	-3.663685	1.471450	-2.566013	N	3.459248	0.942783	4.321481
N	0.489049	2.059274	-1.923015	N	1.796008	-2.998612	4.865461
C	1.131416	3.214215	-1.725228	C	-1.478726	-0.806994	4.340925
C	0.511154	4.436448	-1.914397	C	-2.393428	0.285815	4.281189
H	1.046407	5.366953	-1.752054	C	-3.756442	0.041534	4.081467
C	-0.807121	4.440080	-2.344393	C	-4.266542	-1.236764	3.961314
H	-1.278085	5.372642	-2.624248	H	-5.331429	-1.410055	3.817265
C	-1.502936	3.252239	-2.461842	C	-3.363534	-2.285463	4.042545
H	-2.543085	3.246185	-2.770932	C	-1.996490	-2.102148	4.226603
C	-0.832881	2.046401	-2.229811	H	-1.349314	-2.979671	4.276471
N	4.448915	1.232927	-1.594087	C	2.361092	0.552865	4.452243
N	2.726357	-2.620529	-2.046879	C	1.218511	-1.995225	4.691923
C	-0.510610	-0.380651	-2.219436	K	5.211307	-1.309513	-1.953398
C	-1.414309	0.714601	-2.329448	O	7.231598	0.301002	-3.057900
C	-2.782842	0.465091	-2.483744	K	4.223600	-1.742100	4.625800
C	-3.300879	-0.815489	-2.539563	O	6.243900	-0.131600	3.521400
H	-4.371799	-0.988951	-2.631694	K	5.747400	1.616900	-5.058600
C	-2.400483	-1.864727	-2.438512	K	4.759700	1.184200	1.520800
C	-1.029343	-1.677760	-2.294855	O	6.731299	-0.323700	0.203900
H	-0.385276	-2.555628	-2.214367	O	5.743600	-0.756400	6.783200
C	3.317499	0.925708	-1.695369	Pt	1.766531	0.258880	-5.215003
C	2.169268	-1.592667	-2.002468	F	-2.417847	3.737410	-5.553904
Pt	0.469136	-0.214815	4.474894	F	-3.337411	-0.795989	-5.973601
F	-3.862451	-3.515137	3.924315	N	0.794016	-1.555681	-5.400514
F	-4.634876	1.050191	3.997326	C	1.425980	-2.733312	-5.359457
N	-0.464225	1.636107	4.478044	H	2.461586	-2.714257	-5.020739
C	0.194917	2.787109	4.636188	C	0.807238	-3.905448	-5.754331
C	-0.464973	3.985691	4.841717	H	1.342753	-4.851124	-5.740794
H	0.083363	4.912268	4.978417	C	-0.506879	-3.830321	-6.193341
C	-1.851040	3.970292	4.884883	H	-0.972928	-4.703088	-6.629117
H	-2.387956	4.873435	5.143070	C	-1.200809	-2.635982	-6.124627
C	-2.540466	2.797409	4.644160	H	-2.241285	-2.573603	-6.430980

(Continued)

C	-0.522232	-1.479475	-5.723774	C	-2.273503	-3.029389	0.986673
N	4.711877	-0.858378	-4.746727	H	-3.357545	-2.986686	0.981558
N	3.392197	2.892408	-5.213884	C	-1.527234	-1.846291	0.956308
C	-0.130228	0.926827	-5.513248	N	3.686410	-1.360145	1.677373
C	-1.069720	-0.128308	-5.696195	N	2.289165	2.634983	1.438323
C	-2.429179	0.175670	-5.820288	C	-1.125534	0.570213	1.056838
C	-2.902598	1.474805	-5.772948	C	-2.066989	-0.491175	0.924019
H	-3.968817	1.686495	-5.824568	C	-3.427426	-0.186632	0.803817
C	-1.964731	2.485798	-5.622372	C	-3.906419	1.108931	0.863980
C	-0.601097	2.242437	-5.505459	H	-4.972578	1.318181	0.805683
H	0.076518	3.089083	-5.382412	C	-2.973200	2.121542	1.028287
C	3.605373	-0.521672	-4.937727	C	-1.605887	1.883392	1.105344
C	2.662887	1.977640	-5.212198	H	-0.928467	2.732241	1.217329
Pt	0.797791	-0.085568	1.209101	C	2.622389	-0.919169	1.461758
F	-3.436001	3.368061	1.120061	C	1.645528	1.660827	1.362860
F	-4.338501	-1.154053	0.626825	H	6.547400	-0.686000	7.354700
N	-0.173631	-1.907769	1.028174	H	5.434600	-1.689600	6.880500
C	0.460095	-3.084955	1.041633	H	7.043100	-0.304400	4.077200
H	1.548408	-3.045158	1.067385	H	5.962000	0.787700	3.748000
C	-0.226926	-4.286415	1.052947	H	7.581200	-0.060800	0.636100
H	0.307582	-5.233161	1.069703	H	6.741198	0.120501	-0.669399
C	-1.613529	-4.241481	1.073316	H	8.078899	0.134101	-2.575600
H	-2.180217	-5.154582	1.198915	H	6.985199	1.235301	-2.851700

Table S20 Cartesian coordinates for the optimized structure of Pt6 system (only the ONIOM high-layer).

Pt	2.277400	0.516700	-8.534900	H	-1.312501	5.330596	-2.618962
F	-1.940800	-2.803500	-9.375900	C	-1.530788	3.211181	-2.442334
F	-2.785700	1.793800	-9.228900	H	-2.569795	3.200706	-2.755624
N	1.362400	2.355000	-8.558400	C	-0.861790	2.007188	-2.194869
C	2.037700	3.554500	-8.367500	N	4.398678	1.168959	-1.368395
H	2.965300	3.551800	-8.217900	N	2.744737	-2.634545	-1.904724
C	1.342000	4.757300	-8.398500	C	-0.539838	-0.419439	-2.147118
H	1.798800	5.568200	-8.266700	C	-1.442846	0.674069	-2.283834
C	-0.032400	4.760400	-8.613700	C	-2.808909	0.420790	-2.448282
H	-0.500900	5.575100	-8.635200	C	-3.325198	-0.861638	-2.485841
C	-0.707500	3.562100	-8.806600	H	-4.395040	-1.037675	-2.584656
H	-1.635200	3.564600	-8.955100	C	-2.425581	-1.906727	-2.350488
C	-0.011700	2.359300	-8.777500	C	-1.056519	-1.717260	-2.198611
N	5.128400	1.771600	-8.037400	H	-0.411319	-2.591900	-2.098019
N	3.434300	-2.382400	-8.564100	C	3.271555	0.876546	-1.539367
C	0.342200	-0.060500	-8.859100	C	2.154523	-1.624463	-1.880313
C	-0.543100	1.009700	-8.930200	Pt	0.337176	-0.274338	4.703647
C	-1.897000	0.779000	-9.147700	F	-3.990703	-3.519068	3.997056
C	-2.363600	-0.522000	-9.294000	F	-4.720381	1.066574	4.010507
H	-3.279500	-0.677100	-9.441300	N	-0.563222	1.593953	4.700507
C	-1.479400	-1.591200	-9.222800	C	0.105883	2.734235	4.894359
C	-0.127500	-1.361400	-9.005500	H	1.148424	2.639106	5.192738
H	0.471500	-2.083800	-8.957400	C	-0.485120	3.969690	4.700838
C	4.118200	1.287700	-8.207800	H	0.075776	4.886875	4.853947
C	2.989100	-1.346800	-8.536100	C	-1.803603	4.001686	4.274293
Pt	1.385840	0.165310	-1.834674	H	-2.250049	4.945682	3.994009
F	-2.927832	-3.142028	-2.356653	C	-2.526301	2.828954	4.154215
F	-3.691077	1.423263	-2.559353	H	-3.564962	2.845689	3.839548
N	0.457765	2.023298	-1.877484	C	-1.882617	1.608431	4.383876
C	1.100673	3.181026	-1.696701	N	3.305607	0.743673	5.300472
H	2.144746	3.111619	-1.395194	N	1.717175	-3.065810	4.634963
C	0.482093	4.402302	-1.902162	C	-1.591656	-0.823043	4.360042
H	1.021911	5.333702	-1.754732	C	-2.482559	0.284606	4.262738
C	-0.837184	4.401295	-2.331877	C	-3.849233	0.050467	4.083888

(Continued)

C	-4.377121	-1.225874	3.991444	C	-0.128370	0.942742	-5.494487
H	-5.448164	-1.385313	3.882772	C	-1.072540	-0.114376	-5.634592
C	-3.486152	-2.284402	4.073308	C	-2.427242	0.193677	-5.791287
C	-2.118217	-2.112379	4.246312	C	-2.897524	1.495412	-5.775119
H	-1.478412	-2.994740	4.306524	H	-3.961499	1.709142	-5.852765
C	2.197879	0.423755	5.076471	C	-1.958764	2.503943	-5.625167
C	1.112138	-2.064237	4.653225	C	-0.596763	2.259145	-5.501216
F	-3.756900	6.539900	4.478100	H	0.082890	3.105028	-5.382210
K	6.199000	-0.876800	-8.532800	C	3.604698	-0.508325	-4.926308
O	8.219300	0.733700	-9.637300	C	2.629432	2.011559	-4.986033
K	5.211300	-1.309500	-1.953400	Pt	0.836632	-0.045719	1.380676
O	7.231600	0.301000	-3.057900	F	-3.411822	3.371151	1.018662
K	4.223600	-1.742100	4.625800	F	-4.274261	-1.167782	0.642226
O	6.243900	-0.131600	3.521400	N	-0.107724	-1.875877	1.116993
K	5.747400	1.616900	-5.058600	C	0.540454	-3.045068	1.103346
O	7.269300	0.113400	-6.047000	H	1.626049	-2.998780	1.176532
K	4.759700	1.184200	1.520800	C	-0.130734	-4.252548	1.021987
O	6.731300	-0.323700	0.203900	H	0.411961	-5.193715	1.007499
K	3.772000	0.751500	8.100100	C	-1.516864	-4.227109	0.988069
O	5.743600	-0.756400	6.783200	H	-2.068972	-5.156244	1.019501
F	-2.486200	-6.316700	-6.310800	C	-2.191420	-3.021002	0.952287
Pt	1.780471	0.284592	-5.243921	H	-3.275014	-2.990889	0.903962
F	-2.408887	3.760692	-5.583806	C	-1.459229	-1.829568	1.000136
F	-3.340169	-0.771853	-5.960900	N	3.712386	-1.291771	1.931597
N	0.817083	-1.531916	-5.498144	N	2.374848	2.637131	1.703372
C	1.449679	-2.709010	-5.483934	C	-1.082700	0.590800	1.113589
H	2.537117	-2.671111	-5.445649	C	-2.012065	-0.480628	0.967206
C	0.760926	-3.908736	-5.482293	C	-3.371114	-0.191261	0.809984
H	1.291087	-4.857262	-5.473466	C	-3.860831	1.102143	0.825641
C	-0.624788	-3.861570	-5.454904	H	-4.927370	1.300598	0.742596
H	-1.189223	-4.774003	-5.322716	C	-2.937560	2.124583	0.973138
C	-1.284930	-2.649344	-5.538164	C	-1.571786	1.900092	1.100386
H	-2.369041	-2.603262	-5.535963	H	-0.905240	2.756343	1.217156
C	-0.536307	-1.469496	-5.577256	C	2.644462	-0.865241	1.704605
N	4.680579	-0.894372	-4.665847	C	1.690940	1.693116	1.591027
N	3.305462	2.951581	-4.820883	Pt	-0.190900	-0.506200	7.917400

(Continued)

F	-4.302200	3.026700	7.543200	H	-5.706100	0.971500	7.193000
F	-5.289800	-1.519500	7.067500	C	-3.879000	1.791200	7.529500
N	-1.162600	-2.295000	7.646900	C	-2.535400	1.492200	7.710800
C	-0.525300	-3.529700	7.672100	H	-1.914400	2.182300	7.855100
H	0.401600	-3.576200	7.818600	C	1.624300	-1.371200	8.133100
C	-1.257900	-4.695000	7.479700	C	0.578300	1.317300	8.167300
H	-0.826800	-5.530100	7.499500	H	6.547400	-0.686000	7.354700
C	-2.631400	-4.626200	7.269000	H	5.434600	-1.689600	6.880500
H	-3.124900	-5.415500	7.138300	H	7.043100	-0.304400	4.077200
C	-3.268500	-3.392400	7.241800	H	5.962000	0.787700	3.748000
H	-4.195500	-3.346000	7.096200	H	7.581200	-0.060800	0.636100
C	-2.535800	-2.226900	7.432100	H	6.741200	0.120500	-0.669400
N	2.618600	-1.904900	8.234200	H	8.078900	0.134100	-2.575600
N	1.055500	2.332600	8.279000	H	6.985200	1.235300	-2.851700
C	-2.106700	0.169500	7.678600	H	8.105400	0.273100	-5.543400
C	-3.024600	-0.853200	7.464900	H	7.445600	0.549900	-6.914800
C	-4.370100	-0.553200	7.283500	H	9.042500	0.189600	-9.556400
C	-4.795900	0.769500	7.315900	H	8.023500	0.745300	-10.607800

References.

S1 A. F. Rausch, U. V. Monkowius, M. Zabel and H. Yersin, *Inorg. Chem.*, 2010, **49**, 7818–7825.

S2 *CrysAlisPro*, Rigaku Corporation, Tokyo, Japan, 2015.

S3 G. M. Sheldrick, *Acta Crystallogr. Sect. A*, 2015, **71**, 3–8.

S4 G. M. Sheldrick, *Acta Crystallogr. Sect. C*, 2015, **71**, 3–8.

S5 (a) W. R. Dawson, and M. W. Windsor, *J. Phys. Chem.*, 1968, **72**, 3251–3260; (b) W. H. Melhuish, *J. Phys. Chem.*, 1961, **65**, 229–235.

S6 J. R. Lakowicz, *Principles of Fluorescence Spectroscopy*, Springer, New York, 2006, pp. 141–142.

S7 Gaussian 16, Revision C.01, M. J. Frisch, G. W. Trucks, H. B. Schlegel, G. E. Scuseria, M. A. Robb, J. R. Cheeseman, G. Scalmani, V. Barone, G. A. Petersson, H. Nakatsuji, X. Li, M. Caricato, A. V. Marenich, J. Bloino, B. G. Janesko, R. Gomperts, B. Mennucci, H. P. Hratchian, J. V. Ortiz, A. F. Izmaylov, J. L. Sonnenberg, D. Williams-Young, F. Ding, F. Lipparini, F. Egidi, J. Goings, B. Peng, A. Petrone, T. Henderson, D. Ranasinghe, V. G. Zakrzewski, J. Gao, N. Rega, G. Zheng, W. Liang, M. Hada, M. Ehara, K. Toyota, R. Fukuda, J. Hasegawa, M. Ishida, T. Nakajima, Y. Honda, O. Kitao, H. Nakai, T. Vreven, K. Throssell, J. A. Montgomery, Jr., J. E. Peralta, F. Ogliaro, M. J. Bearpark, J. J. Heyd, E. N. Brothers, K. N. Kudin, V. N. Staroverov, T. A. Keith, R. Kobayashi, J. Normand, K. Raghavachari, A. P. Rendell, J. C. Burant, S. S. Iyengar, J. Tomasi, M. Cossi, J. M. Millam, M. Klene, C. Adamo, R. Cammi, J. W. Ochterski, R. L. Martin, K. Morokuma, O. Farkas, J. B. Foresman and D. J. Fox, Gaussian, Inc., Wallingford CT, 2019.

S8 C. Adamo and V. Barone, *J. Chem. Phys.*, 1999, **110**, 6158–6169.

S9 (a) D. Andrae, U. Häussermann, M. Dolg, H. Stoll and H. Preuss, *Theor. Chim. Acta*, 1990, **77**, 123–141; (b) P. Fuentealba, H. Preuss, H. Stoll and L. v. Szentpály, *Chem. Phys. Lett.*, 1982, **89**, 418-422; (c) T. H. Dunning Jr. and P. J. Hay, in *Modern Theoretical Chemistry, Vol. 3* (Ed.: H. F. Schaefer III), Plenum, New York, 1977, pp. 1–28.

S10 (a) F. Weigend and R. Ahlrichs, *Phys. Chem. Chem. Phys.*, 2005, **7**, 3297–3305; (b) F. Weigend, *Phys. Chem. Chem. Phys.*, 2006, **8**, 1057–1065.

S11 (a) L. W. Chung, W. M. C. Sameera, R. Ramozzi, A. J. Page, M. Hatanaka, G. P. Petrova, T. V. Harris, X. Li, Z. Ke, F. Liu, H-B. Li, L. Ding and K. Morokuma, *Chem. Rev.*, 2015, **115**, 5678–5796; (b) W. M. C. Sameera and F. Maseras, *J. Chem. Info. Model.*, 2018, **58**, 1828–1835.

S12 A. K. Rappé, C. J. Casewit, K. S. Colwell, W. A. Goddard III and W. M. Skiff, *J. Am. Chem. Soc.*, 1992, **114**, 10024–10035.

S13 T. Ziegler and A. Rauk, *Theor. Chim. Acta*, 1977, **46**, 1–10.

S14 (a) M. Mitoraj and A. Michalak, *Organometallics*, 2007, **26**, 6576–6580; (b) M. Mitoraj and A. Michalak, *J. Mol. Model.*, 2008, **14**, 681–687; (c) M. P. Mitoraj, A. Michalak and T. Ziegler, *J. Chem. Theory Comput.*, 2009, **5**, 962–975.

S15 (a) D. Becke, *Phys. Rev. A*, 1988, **38**, 3098–3100; (b) J. P. Perdew, *Phys. Rev. B*, 1986, **33**, 8822–8824.

S16 (a) S. Grimme, J. Antony, S. Ehrlich and H. Krieg, *J. Chem. Phys.*, 2010, **132**, 154104; (b) S. Grimme, S. Ehrlich and L. Goerigk, *J. Comp. Chem.*, 2011, **32**, 1456–1465.

S17 (a) P. Jerabek, P. Schwerdtfeger and G. Frenking, *J. Comput. Chem.*, 2019, **40**, 247–264; (b) L. Zhao, M. von Hopffgarten, D. M. Andrada and G. Frenking, *WIREs Comput. Mol. Sci.*, 2018, **8**, e1345.

S18 (a) E. van Lenthe, E. J. Baerends and J. G. Snijders, *J. Chem. Phys.*, 1993, **99**, 4597–4610; (b) E. van Lenthe, E. J. Baerends and J. G. Snijders, *J. Chem. Phys.*, 1994, **101**, 9783–9792; (c) C. van Wüllen, *J. Chem. Phys.*, 1998, **109**, 392–399.

S19 T. Theiss, S. Buss, I. Maisuls, R. López-Arteaga, D. Brünink, J. Kösters, A. Hepp, N. L. Doltsinis, E. A. Weiss and C. A. Strassert, *J. Am. Chem. Soc.*, 2023, **145**, 3937–3951.

S20 D. Saito, T. Ogawa, M. Yoshida, J. Takayama, S. Hiura, A. Murayama, A. Kobayashi and M. Kato, *Angew. Chem. Int. Ed.*, 2020, **59**, 18723–18730.

S21 M. Kato, C. Kosuge, K. Morii, J. S. Ahn, H. Kitagawa, T. Mitani, M. Matsushita, T. Kato, S. Yano and M. Kimura, *Inorg. Chem.*, 1999, **38**, 1638–1641.
